# Bioanalytical method validation and application to a phase 1, double-blind, randomized pharmacokinetic trial of a standardized *Centella asiatica* (L.) Urban water extract product in healthy older adults

**DOI:** 10.3389/fphar.2023.1228030

**Published:** 2023-08-23

**Authors:** Kirsten M. Wright, Melissa Bollen, Jason David, Bridgette Mepham, Armando Alcázar Magaña, Christine McClure, Claudia S. Maier, Joseph F. Quinn, Amala Soumyanath

**Affiliations:** ^1^ Department of Neurology, Oregon Health & Science University, Portland, OR, United States; ^2^ BENFRA Botanical Dietary Supplements Research Center, Oregon Health & Science University, Portland, OR, United States; ^3^ Department of Chemistry, Oregon State University, Corvallis, OR, United States; ^4^ Linus Pauling Institute, Oregon State University, Corvallis, OR, United States; ^5^ Veterans Affairs Portland Healthcare System Center, Department of Neurology, Portland, OR, United States

**Keywords:** *Centella asiatica*, pharmacokinetics, validation, pharmacodynamics, Alzheimer’s disease, caffeoylquinic acid, triterpene

## Abstract

**Introduction:** *Centella asiatica* is an herbaceous plant reputed in Eastern medicine to improve memory. Preclinical studies have shown that *C. asiatica* aqueous extract (CAW) improves neuronal health, reduces oxidative stress, and positively impacts learning and cognition. This study aimed to develop and validate bioanalytical methods for detecting known bioactive compounds from *C. asiatica* in human biological matrices and apply them to a human pharmacokinetic trial in healthy older adults.

**Methods:** High performance liquid chromatography-tandem mass spectrometry (HPLC-MS/MS) was used for detecting triterpenes and caffeoylquinic acids from *C. asiatica*, or their metabolites, in human plasma and urine. Validation parameters including linearity, precision, accuracy, recovery and thermal stability were evaluated. The method was applied to a Phase I, randomized, double-blind, crossover trial of two doses (2 or 4 g) of a standardized *C. asiatica* water extract product (CAP) in eight healthy older adults. Pharmacokinetic parameters were measured over a 12-h post administration period and acute safety was assessed.

**Results:** The method satisfied US Food & Drug Administration criteria for linearity and recovery of the analytes of interest in human plasma and urine. The method also satisfied criteria for precision and accuracy at medium and high concentrations. Single administration of 2 and 4 g of CAP was well tolerated and safe in healthy older adults. The parent triterpene glycosides, asiaticoside and madecassoside, were not detected in plasma and in minimal amounts in urinary excretion analyses, while the aglycones, asiatic acid and madecassic acid, showed readily detectable pharmacokinetic profiles. Similarly, the di-caffeoylquinic acids and mono-caffeoylquinic acids were detected in low quantities, while their putative metabolites showed readily detectable pharmacokinetic profiles and urinary excretion.

**Discussion:** This method was able to identify and calculate the concentration of triterpenes and caffeoylquinic acids from *C. asiatica*, or their metabolites, in human plasma and urine. The oral absorption of these key compounds from CAP, and its acute safety in healthy older adults, support the use of this *C. asiatica* product in future clinical trials.

## 1 Introduction

Dementia is a debilitating group of diseases that affect memory, thinking, and the ability to perform essential daily activities. More than 55 million adults worldwide have dementia, increasing by almost 10 million new cases annually (World Health Organization, 2023). Alzheimer’s disease (AD) is the most common type of dementia accounting for 60%–80% of cases (Alzheimer’s Association, 2023). In addition to debilitating memory loss, those with AD also experience costly comorbidities including heart and kidney disease, diabetes, stroke, depression, and anxiety (Bunn et al., 2014) making long-term care financially burdensome for individuals and their caregivers (Alzheimer’s Association, 2023).

AD has a complex etiology that includes genetics, environmental exposures, family history, and lifestyle behaviors (McKhann et al., 2011; Livingston et al., 2020). AD’s pathognomonic features are β-amyloid (Aβ) plaques and neurofibrillary tangles caused by abnormal adhesion of tau proteins in cerebral neurons (McKhann et al., 2011). It has been hypothesized that these plaques and tangles disrupt normal neuronal function and synaptic communication. However, interventional trials specifically targeting Aβ clearance have failed to produce substantial results on cognition warranting consideration of alternate etiologies. These include other forms of amyloid, such as small soluble neurotoxic oligomers of the protein (Tolar et al., 2021). Other key components of AD are decreased expression and activity of key mitochondrial enzymes and impaired oxidative phosphorylation, contributing to an increase in free radicals and oxidative damage (Manczak et al., 2004; Baloyannis, 2006; Yao et al., 2009).

Current Food and Drug Administration (FDA) approved AD treatments include cholinesterase inhibitors, N-methyl-D-aspartate (NMDA) receptor agonists, and two targeted immunotherapies (Alzheimer’s Association, 2023). Unfortunately, these therapies are not curative, have numerous side effects, are costly, and do not improve later stages of the disease (moderate or severe AD). Therefore, there is a need to identify additional safe and well-tolerated treatments for AD and its comorbidities, such as depression and sleep disturbances.

Botanical medicines are used by an estimated 80% of the global population and 34 World Health Organization member states list them on the registry of national essential medicines (World Health Organization, 2019). Due to increased interest in and use of botanical drugs, 75 member nations have developed national botanical research institutes, while 99 member nations report a lack of research validation, standardization, safety, and tolerability data on botanical drugs, preventing their integration into national healthcare practices (World Health Organization, 2019). Botanical drugs are chemically complex and offer a diversity of small molecules, which make them intrinsically difficult to study. However, the multiplicity of metabolically active compounds makes botanical drugs good candidates for the treatment of complex disease states (i.e., AD), as these diseases have multiple interventional targets to slow or reverse disease progression (Livingston et al., 2020; Zhang et al., 2021). Rigorous study of botanical drugs using validated methodology and product standardization is needed to increase adoption and integration into global medical practice.

Ethnobotanical, preclinical, and limited clinical trial data indicate that the botanical *Centella asiatica* (L.) Urban is a viable candidate for development as a rational phytotherapeutic for AD (Gray et al., 2018a). *C. asiatica*, also known as gotu kola, is a small herbaceous perennial creeping plant that thrives in moist and swampy terrains of Southeast Asia, sub-equatorial Africa, and New South Wales, and has been naturalized in Madagascar and the Americas (James and Dubery, 2009; Gray et al., 2018a). It has a long history of use in traditional and folk medicine in the Indian sub-continent, South East Asia, parts of China, South Africa and, more recently, the American tropics (Brinkhaus et al., 2000). In traditional and folk medicines, *C. asiatica* is consumed as juice, infusions, and extracts from fresh or dried leaf and stem material for improving mental weakness and memory (Gray et al., 2018a). Today, *C. asiatica* can be found over the counter as a dietary supplement in capsule, tea, powder, or tincture form, or as a standardized product (total triterpenic fraction of *C. asiatica* (TTFCA) and titrated extract of *C. asiatica* (TECA)) (Puttarak et al., 2017).

Preclinical research has shown that an aqueous extract of *C. asiatica* (CAW) improves memory and learning (Rao et al., 2005) in aging and AD without impairing locomotion (Ceremuga et al., 2015) through the modulation of mitochondrial biogenesis, activation of antioxidant response genes, and increasing neuronal dendritic arborization (Gupta et al., 2003; Rao et al., 2006; Gadahad et al., 2008; Gray et al., 2016; Gray et al., 2017a; Matthews et al., 2019). This suggests that CAW may protect neurons from Aβ induced cytotoxicity and apoptosis, thus limiting AD progression without significant side effects (Gray et al., 2014; Gray et al., 2017b). The limited clinical research performed on *C. asiatica* in neurological and neuropsychiatric disorders suggests it improves cognition, stress, anxiety, depression, and quality of life (Brinkhaus et al., 2000; Jana et al., 2010; Gray et al., 2018a) without drowsiness, dizziness, or nausea, which are often experienced with pharmacologic treatment options (Jana et al., 2010).

At least 57 phytochemicals have been identified in the aerial parts, roots, and rhizomes of *C. asiatica* using targeted and non-targeted methods (Alcázar Magaña et al., 2020; Yang et al., 2023). There are two pentacyclic triterpenoid saponins (asiaticoside and madecassoside) and their aglycones (asiatic acid and madecassic acid) that are specific to *C. asiatica* (Azerad, 2016) accounting for up to 8% of its dried mass (Gray et al., 2018a). These triterpenoid compounds (TTs), also known as centelloids, have been found to be responsible for much of *C. asiatica’s* neurotrophic and neuroprotective effects (Gray et al., 2018a); however, caffeoylquinic acids (CQAs; active polyphenolic compounds) accounting for 0.01%–0.47% of the extract have more recently demonstrated similar effects (Gray et al., 2014; Matthews et al., 2020; Alcázar Magaña et al., 2021).

Pharmacokinetic (PK) studies are challenging to perform in botanical medicine due to the variety of therapeutically active compounds in a given plant and their interaction with the complex human plasma matrix (Sorkin et al., 2020). However, they are essential for determining the acute safety, oral absorption, bioavailability, metabolism, and excretion of active compounds thereby informing decisions on dose and dose intervals for future efficacy trials. Valid methodology for identifying the many active compounds in different matrices is the first step towards rigorous PK study. *C. asiatica* PK studies have predominantly focused on isolated triterpene and aglycone compounds or very purified extracts (He et al., 2023), with one recent study using an aqueous extract product and evaluating the triterpenes and CQAs from *C. asiatica* in cognitively impaired older adults receiving cholinesterase inhibitors (Wright et al., 2022). Here we present a validated method for detecting both groups of bioactives in human plasma and urine, and the application of this methodology to a human PK study in healthy older adults.

## 2 Materials and methods

### 2.1 Chemicals

For preparation of calibration standards, plasma and urine work up, and high-performance liquid-chromatography-tandem mass spectrometry analysis (HPLC-MS/MS), asiatic acid (AA; CAS 464-92-6), 1,5-dicaffeoylquinic acid (1,5-DiCQA; CAS 30964-13-7), ferulic acid (FA; CAS 1135-24-6), caffeic acid (CA; CAS 331-39-5), cryptochlorogenic acid (Crypto; CAS 905-99-7), ferulic acid-^13^C_3_ (FA-^13^C_3_; CAS 1261170-81-3), methanol HPLC-MS grade, *Aerobacter aerogenes* sulfatase (10-20 units/mL), *Escherichia coli* glucuronidase (5,00-50,000 units/mL), dextran coated charcoal, and Ultrafree MC-GV spinfilters (durapore-PVDF, 0.22 µm; Millipore Sigma) were purchased from Sigma Aldrich (Darmstadt, Germany). Asiaticoside (AS; CAS 16830-15-2), madecassic acid (MA; CAS 18449-41-7), madecassoside (MS; CAS 34540-22-2), 1,4-dicaffeoylquinic acid (1,4-DiCQA; CAS 1182-34-9), neochlorogenic acid (Neo; CAS 906-33-2), isochlorogenic acid A (IsoA; CAS 2450-53-5), and isochlorogenic acid B (IsoB; CAS 14534-61-3) were purchased from TransMIT (Gießen, Germany). Dihydroferulic acid (DHFA; CAS 1135-23-5) and 3-(3-hydroxyphenyl) propionic acid (HPP; CAS 621-54-5) were purchased from Toronto Research Chemicals (Toronto, ON, Canada). Dihydrocaffeic acid (DHCA; CAS 1078-61-1), 1,3-dicaffeoylquinic acid (1,3-DiCQA; CAS 19870-46-3), chlorogenic acid (CHLA; CAS 327-97-9), isoferulic acid (IFA; CAS 537-73-5), and isochlorogenic acid C (IsoC; CAS 32451-88-0) were purchased from Chromadex (Irvine, CA, United States). Internal standards isoferulic acid-d_3_ (IFA-d_3_; CAS 1028203-97-5) and dihydroisoferulic acid-d_3_ (DHIFA-d_3_; CAS 1258842-21-5) were purchased from Santa Cruz Biotechnology (Dallas, TX, United States). Internal standard chrysin (CAS 207-549-7) and HPLC-MS grade formic acid were purchased from Fluka-Honeywell (Muskegon, MI, United States). Compound identity was confirmed using HPLC-MS/MS and comparison against expected m/z values and retention time standards with a purity of all being 94.8%–99.4%. Acetonitrile (HPLC-MS grade), L-ascorbic acid, and glycerol were purchased from Fisher Scientific (Fairlawn, NJ, United States). HPLC grade de-ionized water was purchased from Macron Fine Chemicals (Radnor, PA, United States).

### 2.2 Sample preparation

An adapted protein precipitation method was used for sample preparation (Cheng et al., 2003). To each sample of participant plasma or urine, *A. aerogenes* sulfatase (2.5 µL; 10–20 units/mL), and *E. coli* glucuronidase (2.5 µL; ∼5,000–50,000 units/mL) were used to release the analytes from their sulfate and β-D-glucuronic acid esters (Phase II metabolites). For calibration and validation samples, aqueous glycerol (5 µL) was added to mimic the enzymatic hydrolysis. For all analyses, 1% ascorbic acid (10 µL) was added prior to sample work up to limit oxidation. Samples were incubated (37°C) for 20 min and placed on ice to halt enzymatic activity. To precipitate matrix proteins and salts, a solution containing internal standards, 75% acetonitrile, and 25% methanol (200 µL) was added, the samples were vortexed and placed at 4°C for 30 min, and the proteins and salts were removed via centrifugation (10,000 g for 5 min at 4°C). The supernatant was filtered (0.22 µm spinfilter) at 10,000 g for 5 min at 4°C and used for analysis. To optimize peak shape during analysis, the concentration of organic solvent (sample filtrate) was adjusted to a 60:40 ratio with HPLC grade water for TT analysis and a 90:10 ratio with 1% aqueous formic acid for CQA analysis.

### 2.3 Instrumentation and HPLC-MS/MS methodology

HPLC-MS/MS was performed at the Oregon Health & Science University’s (OHSU) Bioanalytical Shared Resource/Pharmacokinetics Core (Portland, OR, United States). The analytical method for *C. asiatica*’s triterpenes (TTs) was a modification of that described by Nair et al., 2012. For the detection of TTs and their associated aglycones, HPLC-MS/MS using selected reaction monitoring was performed on an Applied Biosystems Q-Trap 4000 LC-MS (Framingham, MA, United States). Chromatographic separation was achieved using a Poroshell 120 EC18 column (3 mm id × 50 mm; 2.7 µ) and a Poroshell ultra high-performance liquid chromatography (UHPLC) guard column (3.0 mm i.d. Å∼ 5 mm, 2.7* *μ) (Agilent, Santa Clara, CA, United States). The injection volume was 20 μL and flow rate was 0.42 mL/min. Gradient elution was performed using a mobile phase of solvent A (water containing 10 mM ammonium acetate and 0.02% ammonium hydroxide; pH 8.5) and solvent B (methanol). The chromatographic method had a duration of 9 min, and the gradient design was: an initial 2 min increase from 40% to 60% B, followed by 60%–95% B from 2 to 3.5* *min, hold at 95% B from 3.5–6* *min, return to 40% B by 6.1* *min, and re-equilibrate at 40% B from 6.1–9 min. TTs were detected as their ammonium adducts with positive ion mode electrospray ionization using the following MS/MS transitions (m/z): AA (506/453), MA (522/451), AS (976/453; 976/635), and MS (992/487; 992/451). The internal standard chrysin was detected as the molecular ion (255/255). For the detection of CQAs and their associated metabolites, the HPLC-MS/MS method developed by the authors was performed on an Applied Biosystems 5500 QTRAP HPLC-MS instrument (Framingham, MA, United States). Chromatographic separation was achieved using a Zorbax Eclipse plus C8 Rapid resolution (4.6 i.d. Å∼ 150 mm, 3.5 μ) column with a Zorbax Eclipse plus C8 Rapid resolution (4.6 i.d. Å∼ 12.5 mm, 5 μ) guard column (Agilent, Santa Clara, CA, United States). The injection volume was 5 μL and flow rate of 0.8 mL/min. Gradient elution was performed using a mobile phase of solvent A (water containing 0.05% v/v acetic acid) and solvent B (acetonitrile with 0.05% v/v acetic acid). The chromatographic method had a duration of 21 min, and the gradient design was as follows: an initial 0.1 min at 10% B, an increase to 25% B by 4.5 min, 25%–40% B from 4.5 to 10 min, 40%–95% B from 10 to 11 min, hold at 95% B from 11 to 16 min, return to 10% B by 16.2 min, and re-equilibrate at 10% from 16.2 to 21 min. All CQAs, metabolites, and internal standards were detected using negative ion mode electrospray ionization and the following MS/MS transitions (m/z): mono-CQAs (353/191); di-CQAs (515/353; 515/191); CA (179/135); FA and IFA (193/134); DHCA (181/109); DHFA (195/136), HPP (165/106), ferulic acid-^13^C_3_ (196/136), IFA-d_3_ (196/134), and DHIFA-d_3_ (198/136).

### 2.4 Data analysis

Analyst software from Sciex technologies was used to identify and integrate peaks for analytes and internal standards in each sample. Peak area, peak height, and retention time for each analyte and internal standard were recorded. For analytes with multiple transitions, the most appropriate transition was selected on an experiment-by-experiment basis, based on peak height, peak shape, and amount of baseline noise. Those transitions with the greatest peak height, smoothest peak shape, and minimal baseline noise were used in the analysis. In each experiment, the same transitions were used for both calibration and unknown samples.

### 2.5 Method validation

Method validation parameters (linearity, accuracy, precision, stability, and recovery) were evaluated following FDA guidelines (Food and Drug Administration, 2022). Analyses were performed in two matrices, human plasma and urine, to be applied to oral absorption and urinary excretion analyses. To account for matrix effects, the analytes of interest were assessed in pooled human plasma (apheresis derived, with sodium heparin) purchased from Innovative Research (St. Louis, MO, United States). The plasma was charcoal treated to remove any TTs or CQAs resulting from the plasma donors’ diet that may artificially inflate plasma analyte concentrations. The plasma was mixed with dextran treated charcoal (1 mL:20 mg) and placed on a shaker at 4°C overnight to allow for binding to hormones, steroids, xenobiotics, and proteins. Samples were centrifuged for 5 min at 10,000 g at 4°C to remove the charcoal and the supernatant was used for future experiments. Urine validation was performed in pooled baseline urine from healthy older adults consuming a 48-h low phytochemical diet void of plant-based foods (nuts, seeds, coffee, tea, chocolate, fruits, vegetables, spices, and whole grains) to remove the analytes of interest.

*Calibration, linearity, accuracy, and precision:* Six analyte concentrations were added to 50 µL aliquots (*n* = 5) of charcoal treated plasma on ice (Table 1). Six analyte concentrations were added to 50 µL aliquots (*n* = 5) of urine on ice (Table 2). See Section 2.2 for sample preparation. Separate calibration curves were constructed by plotting the analyte peak area and the analyte peak area ratio respective to the internal standard (IS) to the known analyte concentration. Total rather than individual mono- or di-CQA concentrations and areas were used for calibration curves and quantitation of test samples to allow for the possibility of interconversion of isomers that is known to occur for these compounds (Xue et al., 2016; Dawidowicz and Typek, 2017). The values were fitted with a linear regression best fit line and the coefficient of determination (R^2^) was used to assess variability. The slope of the linear regression best fit line and the standard error of the intercept of the line were used to calculate the limit of detection (LOD) [LOD = 3.3 × (standard error of the intercept/slope of the calibration curve)]. The lower limit of quantitation (LLoQ) was determined by identifying the lowest concentration tested that satisfied FDA guidelines for both precision and accuracy (Food and Drug Administration, 2022). Precision (%, relative standard deviation (RSD) and accuracy (% of nominal amount) were determined by analyzing the replicates of each concentration for each analyte. Intraday precision (RSD) and accuracy (%, percent relative error) were determined by analyzing the same replicate injected at different timepoints throughout the run (*n* = 6). Per the FDA guidelines, precision should be within 15% and no greater than 20% deviation at the LLoQ. Accuracy acceptance criteria is 100 ± 15% of the known value and 100 ± 20% at the LLoQ.

**TABLE 1:** Final analyte concentration of *C. asiatica* triterpenes, caffeoylquinic acids, and their metabolites added to human plasma for assessment of linearity, accuracy, precision, and recovery.

Condition	1	2	3	4	5	6
Analyte	Concentration (ng/mL)					
AA	0	30	120	240	480	600
AS	0	2.5	10	20	40	50
CQA metabolites^a^	0	1	4	8	16	20
CQAs^b^	0	1	4	8	16	20
HPP	0	5	20	40	80	100
MA	0	15	60	120	240	300
MS	0	2.5	10	20	40	50

*C. asiatica* triterpene and caffeoylquinic acid analytes were prepared in charcoal treated blank human plasma. AA, asiatic acid; AS, asiaticoside; HPP, 3-(3-hydroxyphenyl)propionic acid; MA, madecassic acid; MS, madecassoside.

^a^ Caffeoylquinic acid putative metabolites include caffeic acid (CA), ferulic acid (FA), dihydrocaffeic acid (DHCA), dihydroferulic acid (DHFA), dihydroisoferulic acid (DHIFA), isoferulic acid (IFA).

^b^ Independent caffeoylquinic acids include chlorogenic acid (CHLA), cryptochlorogenic acid (Crypto), neochlorogenic acid (Neo), 1,3-dicaffeoylquinic acid (1,3-diCQA), 1,4-dicaffeoylquinic acid (1,4-diCQA), 1,5-dicaffeoylquinic acid (1,5-diCQA), isochlorogenic acid A (IsoA), isochlorogenic acid B (IsoB), isochlorogenic acid C (IsoC).

**TABLE 2:** Final analyte concentration of *C. asiatica* triterpenes, caffeoylquinic acids, and their metabolites added to human urine for assessment of linearity, accuracy, precision, and recovery.

Condition	1	2	3	4	5	6
Analyte	Concentration (ng/mL)					
AA	0	3	7.5	15	22.5	30
AS	0	6	15	30	45	60
CQA metabolites^a^	0	5	12.5	25	37.5	50
MA	0	3	7.5	15	22.5	30
MS	0	6	15	30	45	60
Total di-CQAs^b^	0	3	7.5	15	22.5	30
Total mono-CQAs^c^	0	1.5	3.75	7.5	11.25	15

*C. asiatica* triterpene and caffeoylquinic acid analytes were prepared in pooled blank human urine from humans following a low phytochemical diet for 48-h prior to collection. AA, asiatic acid; AS, asiaticoside; MA, madecassic acid; MS, madecassoside.

^a^ Caffeoylquinic acid putative metabolites include: caffeic acid (CA), ferulic acid (FA), DHFA (dihydroferulic acid), 3-(3-hydroxyphenyl)propionic acid (HPP), dihydrocaffeic acid (DHCA), dihydroisoferulic acid (DHIFA), isoferulic acid (IFA).

^b^ Di-caffeoylquinic acids include: 1,3-dicaffeoylquinic acid (1,3-diCQA), 1,4-dicaffeoylquinic acid (1,4-diCQA), 1,5-dicaffeoylquinic acid (1,5-diCQA), isochlorogenic acid A (IsoA), isochlorogenic acid B (IsoB), isochlorogenic acid C (IsoC).

^c^ Mono-caffeoylquinic acids include: chlorogenic acid (CHLA), cryptochlorogenic acid (Crypto), neochlorogenic acid (Neo).

*Recovery:* Aliquots (50 µL) of charcoal treated plasma (*n* = 5) without added analytes were processed in parallel with the above samples using the same processing method (Section 2.2). Immediately following protein precipitation but prior to filtration, analytes were added to the plasma at low (concentration 2 in Table 1), medium (concentration 4 in Table 1), and high (concentration 6 in Table 1) concentrations. These samples represented 100% recovery for analysis. Calibration curves were used to calculate the amount (ng/mL) of analytes present in the recovery samples. Recovery was calculated as the percent difference in mean concentration between the samples with analytes added prior to protein precipitation and the samples where they were added after precipitation.

*Stability:* The same concentrations used in the recovery experiments (low, medium, high) were used to assess analyte stability in plasma (Table 1) and urine (Table 2). Analytes were mixed with 50 µL of charcoal treated plasma (*n* = 5) or urine (*n* = 5) and placed in each of the following storage conditions: 1) long-term storage (two weeks at −80°C); 2) three freeze-thaw cycles during a two-week period stored at −80°C; 3) 4°C for 24 h; and 4) fresh preparation for same day analysis. All samples were processed together for analysis. Calibration curves generated from storage condition 4 samples were used to determine the concentration of analyte present in each storage condition. The percent difference from samples in condition 4 was used to determine stability. Interday stability was not measured directly as calibration curves were to be prepared for each participant using their baseline samples and analyzed concurrently with test samples. Comparisons between calibration curves were performed using changes in the slope of the line as an indicator of stability.

### 2.6 Application to a pharmacokinetic study in healthy older adults

*Institutional Review Board Statement:* The study was conducted in accordance with the Declaration of Helsinki and approved by the Oregon Health & Science University (OHSU) Institutional Review Board (IRB number: 17697, date of approval: 21 March 2019). This study was registered with the United States National Library of Medicine Clinical Trials Registry (NCT03929250, date of registration: 26 April 2019).

*Informed Consent Statement:* Written informed consent was obtained from all subjects involved in the study prior to the initiation of any study activities.

*C. asiatica water extract product (CAP):* Allometric scaling (Nair and Jacob, 2016) from preclinical mouse studies (200-1,000 mg/kg/day) (Gray et al., 2014; Gray et al., 2015; Gray et al., 2016; Gray et al., 2018b; Matthews et al., 2020; Zweig et al., 2021) was used to identify a dose range of 2–10 g of *C. asiatica* water extract (CAW) daily for a 70 kg human. Two doses (2 and 4 g CAW) were selected from within the calculated dose range and prepared as described in Wright et al. (2021). Briefly, aqueous extracts of *C. asiatica* dry herb aerial parts were prepared by Ashland Laboratories (Paterson, NJ, United States), spray dried onto a carrier matrix, and blended with inert flavor and color agents (excipients) making a powdered formula called CAW product (CAP). Doses were packaged into individual opaque sachets for dispensing and analyzed using HPLC-MS/MS mass spectral fingerprinting to confirm dosage (Alcázar Magaña et al., 2020). The product was stored in the dark at −20°C during the study.

*Sample size:* A minimum sample size of six participants was calculated using changes in the response values of two dose-dependent PK parameters [peak concentration (C_max_) and area under-the-curve (AUC)] at two treatment doses found in the literature for AA (Grimaldi et al., 1990). Calculations are presented in Wright et al., 2022. Briefly, a sample size of six was determined to be sufficient to detect the established criterion difference (critical t = 2.57) with both the mean and median test statistics being greater than the critical t. Target recruitment was eight participants, four males and four females, allowing for a 25% dropout. Sample size was not calculated using the additional pharmacokinetic parameters of time of maximum concentration (T_max_) and elimination half-life (t_½_) because these were determined to not be dose dependent parameters.

**FIGURE 1:**
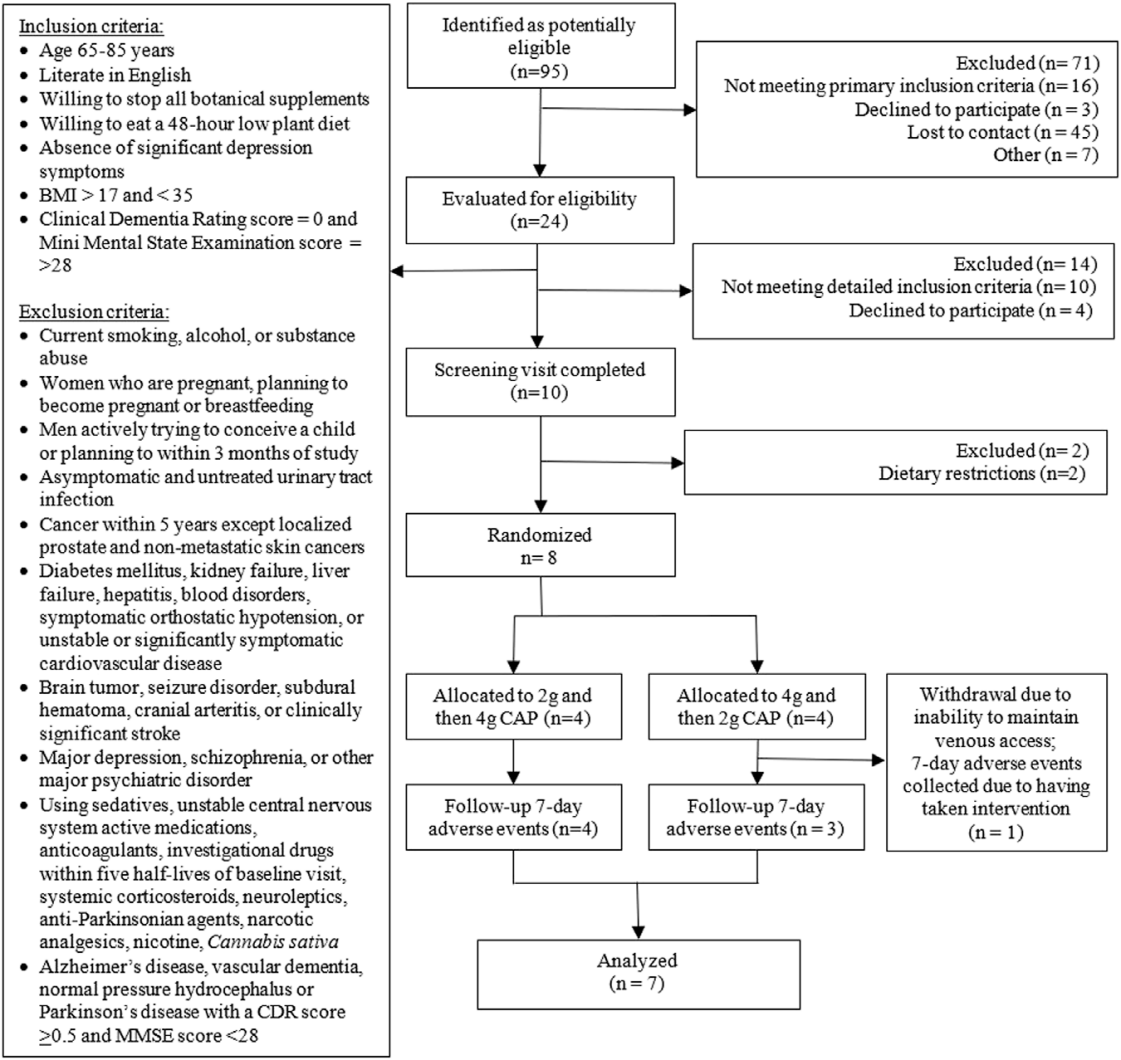
Consort flow diagram.

*Participant eligibility criteria:* Healthy older adults aged 65–85 years were recruited from the Portland, Oregon metropolitan area between July and November 2019. Potential participants (*n* = 95) were identified using the Oregon Clinical and Translational Research Institute’s (OCTRI) research volunteer repository, the National Institute of Neurological Disorders and Stroke (NINDS) NeuroNext database, the National Institute of Aging’s Oregon Alzheimer’s Disease Research Center’s participant registry ACTNOW, the OHSU March Wellness monthly advertisement, and fliers posted in OHSU academic buildings and clinics. A total of 10 potential participants were identified by examination of electronic medical records and telephone screening and brought in for a screening visit (Figure 1). Potential participants were excluded if they had: memory impairment (Clinical Dementia Rating Score ≥0.5) or diseases associated with dementia (i.e., AD or vascular dementia); major psychiatric disorders including schizophrenia, substantial depressive symptoms, or substance use disorder; smoked tobacco; a body mass index <17 or >35; an active urinary tract infection; or a cancer history within five years. Persons taking the following medication classes were excluded: sedatives, unstable central nervous system active medications (<2 months), anticoagulants, investigational drugs used within five half-lives, systemic corticosteroids, neuroleptics, anti-Parkinsonian agents, narcotic analgesics, nicotine, or *Cannabis sativa*. Persons were also excluded if they had: diabetes mellitus, kidney and/or liver failure, hepatitis, blood disorders, orthostatic hypotension, unstable or significantly symptomatic cardiovascular disease, or a disease of the central nervous system (i.e., brain tumor, seizure disorder, subdural hematoma, cranial arteritis, normal pressure hydrocephalus, Parkinson’s disease, or a stroke). Eight volunteers, four males and four females, were enrolled.

*Study design:* This single-center, randomized, double-blind, crossover study occurred at the OCTRI’s Clinical Trials Research Center (CTRC) at OHSU under a US Food and Drug Investigational New Drug assignment (#13066) using the study design of a prior PK trial in cognitively impaired older adults receiving cholinesterase inhibitors (Wright et al., 2022). Briefly, two in-person study visits occurred a minimum of two weeks and a maximum of four weeks apart in which participants orally consumed 2 or 4 g of CAP (in a randomized order) on an empty stomach. Total study duration was eight weeks including follow-up adverse event monitoring. All study procedures were identical for each visit except for the CAP dose administered. Randomization (to prevent tolerance effects) was performed using an arm equivalence design by the OHSU Research Pharmacy. All study personnel were blind to randomization allocation until all data analysis had been finalized. Participants were placed on a low-phytochemical diet for 48 h prior to each study visit to minimize dietary interference and compliance verified via a diet diary. Participants fasted for 10 h, excluding water, prior to each study visit and for 2 h after consuming the study intervention to standardize gastrointestinal transit time and minimize food effects. During study visits, participants were provided low-phytochemical snacks and meals by the OCTRI CTRC’s Bionutrition Unit devoid of the foods outlined in Section 2.5. At each study visit, a peripheral intravenous catheter was placed in the arm to allow for repeat blood sampling. Prior to CAP intake, 20 mL of baseline blood and 50 mL of baseline urine were collected. To maintain blinding, study personnel dissolved the allocated CAP dose in 10 ounces of warm water, and the participants orally consumed the solution as one bolus. Blood samples (10 mL) were collected over 12 h (0.25, 0.5, 0.75, 1, 1.5, 2, 3, 4, 6, 8, 9, 10, and 12 h), transferred to a BD Vacutainer™ heparinized tube (Fisher Scientific, NJ, United States), and centrifuged for 10 min at 10,000 g and 4°C to isolate plasma. Plasma aliquots (2 mL) were frozen and stored at -80°C until analysis. Total urine output was collected in a 2 L collection container for the 12-h post-administration period and stored at 4°C. A 50 mL aliquot was retained and frozen at −80°C for renal excretion analysis.

*Safety assessments:* Using a multisystem questionnaire, acute tolerability and safety assessments were completed at each study visit (baseline and 12 h), at 24 h, and 7 days post-administration to identify delayed adverse events. Each event was ranked on a 0–5 scale based upon the following criteria: 0 = absent, 1 = mild, 2 = moderate, 3 = severe, 4 = life-threatening, and 5 = fatal. A resting electrocardiogram (ECG) was collected at baseline and 6 h post-administration to identify asymptomatic cardiovascular changes.

*Sample preparation and analysis:* Plasma and urine sample preparation and analyte quantification by LC-MS/MS were performed in duplicate for each time point and visit (Sections 2.2–2.4). Due to the possibility of residual interference from the participant’s diet and unique, individual matrix effects, calibration curves were generated for each participant using their baseline plasma collected at the corresponding visit. The calibration curves showed good linearity for the TTs (R^2^ = 0.97–0.99), the CQAs (R^2^ = 0.97–0.99), and the putative CQA metabolites (R^2^ = 0.95–0.99). For renal excretion analyses, calibration curves were generated in pooled participant baseline urine and samples were prepared using the plasma preparation method involving enzymatic hydrolysis, as it was previously reported the analytes are predominantly found in the conjugated form (Wright et al., 2022). The PK parameters peak concentration (C_max_), area under-the-curve (AUC_0-12_), elimination half-life (t_1/2_), and time to peak concentration (T_max_), and the concentration *versus* time curves, were calculated using a non-compartmental analysis of average plasma concentration (from all participants) *versus* time using Excel software PK-solver (version 2.0). Two-sided paired t-tests were used to compare the PK and clearance parameters between the doses, and to compare between the male and female participants to assess for the effect of sex on oral absorption. A *p*-value of 0.05 or less was considered statistically significant.

## 3 Results

### 3.1 Validation

*Plasma calibration and linearity* **:** Each of the analytes was detected in human plasma using the HPLC-MS/MS parameters outlined above (Supplementary Figures S1–S6). Compared with blank plasma, there is a small chance of interference from other matrix components detected at the retention time of the respective peaks of interest; however, the amounts found in the blank plasma are very low (Supplementary Figures S3, S4). For each analyte, the linearity of peak area of analyte to concentration or peak area ratio (analyte/internal standard) to concentration was calculated (Table 3) over the concentration ranges given in Tables 1, 2. TTs showed good linearity for the aglycones AA and MA (area R^2^ = 0.94–0.99, area ratio R^2^ = 0.95–0.96) with parent glycoside MS showing good linearity for peak area (R^2^ = 0.94) but not for area ratio (R^2^ = 0.82) and AS showing less linearity (area R^2^ = 0.82, area ratio R^2^ = 0.75) (Table 3). The CQAs and metabolites showed good linearity for both peak area (R^2^ = 0.91–0.99) and area ratio (R^2^ = 0.89–0.99), except for total mono-CQAs (area R^2^ = 0.60, area ratio R^2^ = 0.56) and DHCA (area R^2^ = 0.82, area ratio R^2^ = 0.82).

**TABLE 3:** Linearity of TTs and CQAs from *C. asiatica* in blank human plasma and urine.

Analyte	Average peak area (R^2^)		Average area ratio (R^2^)		Internal standard
	Plasma	Urine	Plasma	Urine	Plasma
AA	0.94	0.99	0.95	0.99	Chrysin
AS	0.82	0.92	0.75	0.99	Chrysin
CA	0.99	0.99	0.99	0.92	FA^13^
DHCA	0.82	0.99	0.82	0.99	FA^13^
DHFA	0.99	0.96	0.99	0.96	DHIFA_d3_
Di-CQAs^a^	0.91	0.99	0.89	0.99	FA^13^
FA	0.99	0.99	0.99	0.99	FA^13^
HPP	0.99	0.99	0.99	0.99	FA^13^
IFA	0.99	0.99	0.99	0.99	IFA_d3_
MA	0.99	0.98	0.96	0.99	Chrysin
Mono-CQAs^b^	0.60	0.92	0.56	0.92	FA^13^
MS	0.94	0.98	0.82	0.98	Chrysin

*C. asiatica* triterpene and caffeoylquinic acid analytes prepared in charcoal treated blank human plasma and blank human urine from humans following a low phytochemical diet for 48-h prior to collection. AA, asiatic acid; AS, asiaticoside; CA, caffeic acid; DHCA, dihydrocaffeic acid; DHFA, dihydroferulic acid; FA, ferulic acid; HPP, 3-(3-hydroxyphenyl)propionic acid; IFA, isoferulic acid; MA, madecassic acid; MS, madecassoside; FA^13^ = ferulic acid-^13^C_3_; DHIFA_d3_ = dihydroisoferulic acidd_3_; IFA_d3_ = isoferulic acid-d_3_.

^a^ Total di-caffeoylquinic acids [1,3-dicaffeoylquinic acid (1,3-diCQA), 1,4-dicaffeoylquinic acid (1,4-diCQA), 1,5-dicaffeoylquinic acid (1,5-diCQA), isochlorogenic acid A (IsoA), isochlorogenic acid B (IsoB), isochlorogenic acid C (IsoC)].

^b^ Total mono-caffeoylquinic acids [chlorogenic acid (CHLA), cryptochlorogenic acid (Crypto), neochlorogenic acid (Neo)].

*Urine calibration and linearity:* Each of the analytes were detected in human urine using the HPLC-MS/MS parameters outlined above (Supplementary Figures S7–S12). Compared with blank urine, no interference from other matrix components was detected at the retention time of the respective peaks of interest (Supplementary Figures S9, S10). The TTs showed good linearity (area R^2^ = 0.92–0.99, area ratio R^2^ = 0.98–0.99) (Table 3). All CQA samples showed good linearity (R^2^ area and area ratio = 0.92–0.99).

*Plasma accuracy and precision:* At high and medium concentrations, accuracy remained within the specified limits (100% ± 15%) for all analytes except DHCA (medium concentration) using either area or area ratio for quantitation (Table 4). At the lowest concentration, several analytes (AS, MS, CA, DHCA, DHFA, and mono-CQAs) lost accuracy using area and/or area ratio for quantification. The majority of compounds retained acceptable precision (RSD ≤15%) across the entire concentration range using both peak area or area ratio (Table 4). Exceptions included AS, IFA, mono-CQAs, di-CQAs, and MS which lost precision only at the lowest concentration, and DHCA which was outside the range of acceptable precision for all concentrations. MA showed a slight deviation (RSD 17% and 16% for area and area ratio, respectively) at the medium concentration, while MS had poor precision (RSD >30%) at the lowest concentration but close to acceptable precision (RSD 16%–18%) at the medium and high concentrations. The LLoQ based on the concentration range tested and the FDA guidance for limits of accuracy at the LLoQ (100 ± 20%) and precision (RSD ≤20%) ranged from 2.5–30 ng/mL for TTs and 1–48 ng/mL for CQAs (Table 4). Intraday analyses showed good precision and accuracy at medium and higher concentrations for all analytes (Supplementary Table S1).

**TABLE 4:** Accuracy and precision for triterpenes and caffeoylquinic acids from *C. asiatica* at three quality control concentrations, calculated both without and with internal standard for each compound added to blank charcoal treated human plasma.

Analyte	LoD ng/mL	LLoQ ng/mL	Accuracy (%)						Precision (%)					
			QC1		QC2		QC3		QC1		QC2		QC3	
			No IS	With IS	No IS	With IS	No IS	With IS	No IS	With IS	No IS	With IS	No IS	With IS
AA	0.31	≤30	107	96	105	97	99	101	6	13	11	11	4	5
AS	1.62	2.5–20	125	101	105	97	99	101	27	29	13	14	5	7
CA	2.0 × e-6	1–8	122	122	91	91	102	102	15	15	5	5	2	2
DHCA	2 × e-3	8–20	141	141	77	77	105	105	47	47	47	47	19	19
DHFA	2.0 × e-5	1–8	155	154	89	89	102	102	8	8	5	5	8	8
Di-CQAs^a^	0.013	6–48	99	99	90	90	102	102	57	57	4	4	5	5
FA	3 × e-3	≤1	105	105	89	89	103	103	14	14	6	6	7	7
HPP	5.0 × e-4	≤5	109	109	87	87	103	103	8	8	6	6	5	5
IFA	3.0 × e-5	1–8	113	108	95	91	101	102	29	26	13	15	10	10
MA	0.11	≤15	99	90	107	99	98	100	7	9	17	16	4	4
Mono-CQAs^b^	5 × e-3	3–24	71	71	94	94	102	102	25	25	14	14	9	9
MS	4.30	2.5–20	193	137	92	86	102	103	32	37	12	16	18	18

LOD, Limit of detection = 3.3 × (standard error of the intercept/slope of the calibration curve); LLoQ = Lower limit of quantification = smallest tested concentration at which accuracy and precision were ±20%; QC1: AA 30 ng/mL, MA 15 ng/mL, AS 2.5 ng/mL, MS 2.5 ng/mL, HPP 5 ng/mL, CQAs and metabolites 1 ng/mL; QC2: AA 240 ng/mL, MA 120 ng/mL, AS 20 ng/mL, MS 20 ng/mL, HPP 40 ng/mL, CQAs and metabolites 8 ng/mL; QC3: AA 600 ng/mL, MA 300 ng/mL, AS 50 ng/mL, MS 50 ng/mL, HPP 100 ng/mL, CQAs and metabolites 20 ng/mL. No IS = results obtained with calibration curves constructed by linear regression analysis of the analyte area vs. the concentration of analytes injected; With IS = results obtained with calibration curves constructed by linear regression analysis of the ratio of the analyte area to the internal standard (IS) area vs. the concentration of analytes injected. Precision is expressed as RSD = (standard deviation/mean concentration measured) × 100. Accuracy was determined as (mean measured concentration/known added concentration) × 100. AA, asiatic acid; AS, asiaticoside; CA, caffeic acid; DHCA, dihydrocaffeic acid; DHFA, dihydroferulic acid; FA, ferulic acid; HPP, 3-(3-hydroxyphenyl)propionic acid; IFA, isoferulic acid; MA, madecassic acid; MS, madecassoside.

^a^ Di-caffeoylquinic acids [1,3-dicaffeoylquinic acid (1,3-diCQA), 1,4-dicaffeoylquinic acid (1,4-diCQA), 1,5-dicaffeoylquinic acid (1,5-diCQA), isochlorogenic acid A (IsoA), isochlorogenic acid B (IsoB), isochlorogenic acid C (IsoC)].

^b^ Mono-caffeoylquinic acids [chlorogenic acid (CHLA), cryptochlorogenic acid (Crypto), neochlorogenic acid (Neo)].

*Urine accuracy and precision:* All compounds were quantified with acceptable accuracy (100% ± 15%) and precision (RSD ≤15%) at the highest concentrations analyzed (Table 5). Several analytes (DHCA, di-CQAs, FA, HPP, DHFA, and IFA) retained accuracy and precision across the concentration range using peak area and/or area ratio. The mono-CQAs lost accuracy at the lowest concentration. AA and CA maintained accuracy and precision using the criteria of ±20% at the LLoQ at the lowest concentration. AS, MA, and MS lost precision at the lowest concentration. The LLoQ based on the concentration range tested and the FDA guidance for limits of accuracy at the LLoQ (100% ± 20%) and precision (RSD ≤20%) ranged from 3–30 ng/mL for TTs and ≤2.5–7.5 ng/mL for CQAs (Table 5).

**TABLE 5:** Accuracy and precision for triterpenes and caffeoylquinic acids from *C. asiatica* at three quality control concentrations, calculated both without and with internal standard for each compound added to blank human urine.

Compound	LoD ng/mL	LLoQ ng/mL	Accuracy (%)						Precision (%)					
			QC1		QC2		QC3		QC1		QC2		QC3	
			No IS	With IS	No IS	With IS	No IS	With IS	No IS	With IS	No IS	With IS	No IS	With IS
AA	1.3 × e-2	3	80	152	89	89	103	102	29	19	16	11	6	12
AS	6	18	115	98	101	99	105	103	36	41	6	13	10	11
CA	5 × e-2	≤5	117	355	89	107	113	105	8	7	1	4	4	6
DHCA	2 × e-3	≤5	105	127	94	99	102	101	14	14	5	6	1	4
DHFA	3 × e-3	≤5	110	91	85	100	100	96	15	9	3	4	8	8
Di-CQAs^a^	2 × e-4	≤2.5	110	100	111	114	101	97	11	10	9	9	11	4
FA	2 × e-4	≤5	100	102	94	106	102	98	8	14	5	5	7	4
HPP	6 × e-4	≤5	130	95	94	98	101	103	12	5	3	5	3	4
IFA	5 × e-4	≤5	99	106	92	98	103	99	10	2	9	3	5	5
MA	8	15	95	48	85	83	107	105	24	23	11	9	6	12
Mono-CQAs^b^	1 × e-3	7.5	152	136	110	109	107	105	30	25	7	7	6	7
MS	19	30	93	67	86	91	107	102	40	50	12	16	8	8

LOD, limit of detection, = 3.3 × (standard error of the intercept/slope of the calibration curve); LLoQ = Lower limit of quantification = smallest tested concentration at which accuracy and precision were ±20%; QC1: AA 3 ng/mL, MA 3 ng/mL, AS 6 ng/mL, MS 6 ng/mL, HPP, DHFA, and CQA metabolites 5 ng/mL, CQAs (each) 0.5 ng/mL; QC2: AA 15 ng/mL, MA 15 ng/mL, AS 30 ng/mL, MS 30 ng/mL, HPP, DHFA, and CQA metabolites 25 ng/mL, CQAs (each) 2.5 ng/mL; QC3: AA 30 ng/mL, MA 30 ng/mL, AS 60 ng/mL, MS 60 ng/mL, HPP, DHFA, and CQA metabolites 50 ng/mL, CQAs (each) 5 ng/mL. No IS = results obtained with calibration curves constructed by linear regression analysis of the analyte area vs. the concentration of analytes injected; With IS = results obtained with calibration curves constructed by linear regression analysis of the ratio of the analyte area to the internal standard (IS) area vs. the concentration of analytes injected. Precision is expressed as RSD = (standard deviation/mean concentration measured) × 100. Accuracy was determined as (mean measured concentration/known added concentration) x 100. AA, asiatic acid; AS, asiaticoside; CA, caffeic acid; DHCA, dihydrocaffeic acid; DHFA, dihydroferulic acid; FA, ferulic acid; HPP, 3-(3-hydroxyphenyl)propionic acid; IFA, isoferulic acid; MA, madecassic acid; MS, madecassoside.

^a^ Di-caffeoylquinic acids [1,3-dicaffeoylquinic acid (1,3-diCQA), 1,4-dicaffeoylquinic acid (1,4-diCQA), 1,5-dicaffeoylquinic acid (1,5-diCQA), isochlorogenic acid A (IsoA), isochlorogenic acid B (IsoB), isochlorogenic acid C (IsoC)].

^b^ Mono-caffeoylquinic acids [chlorogenic acid (CHLA), cryptochlorogenic acid (Crypto), neochlorogenic acid (Neo)].

*Recovery:* Recovery was assessed as the percent difference from the nominal amount added prior to sample processing. The observed recovery of the analytes from plasma ranged from 96% to 246% of the added amount. At high concentrations, AS, IFA, MA, mono-CQAs, and MS had a >100% recovery. At medium concentrations, all analytes except IFA had a >100% recovery. At low concentrations, AA, CA, DHFA, and HPP had a >100% recovery. At low concentrations, the mono-CQAs, MS, AS, and di-CQAs had low recovery being undetected in the sample.

**FIGURE 2:**
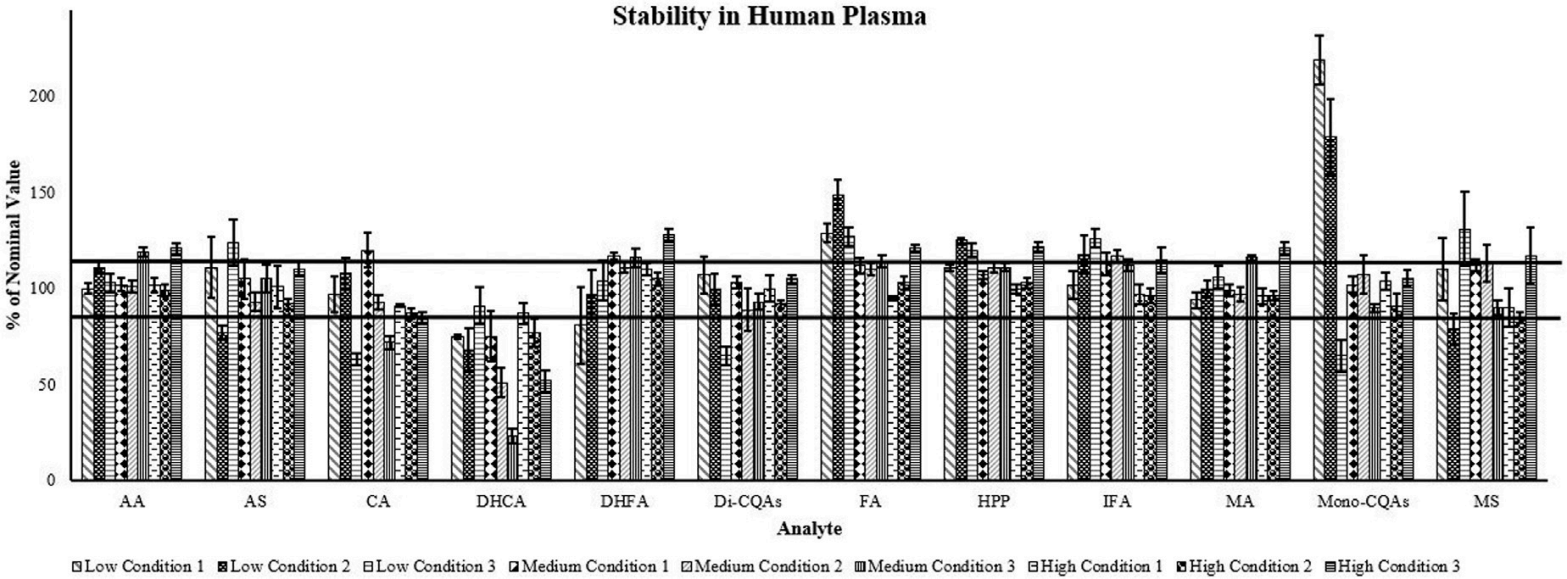
Triterpene and caffeoylquinic acid stability of three different concentrations in human plasma. Data are presented as percent of nominal concentration ± SEM (*n* = 5). Low: AA 30 ng/mL, MA 15 ng/mL, AS 2.5 ng/mL, MS 2.5 ng/mL, HPP 5 ng/mL, CQAs and metabolites 1 ng/mL; Medium: AA 240 ng/mL, MA 120 ng/mL, AS 20 ng/mL, MS 20 ng/mL, HPP 40 ng/mL, CQAs and metabolites 8 ng/mL; High: AA 600 ng/mL, MA 300 ng/mL, AS 50 ng/mL, MS 50 ng/mL, HPP 100 ng/mL, CQAs and metabolites 20 ng/mL. Condition 1: Long-term storage at −80°C. Condition 2: Three rounds of freeze thaw and storage at −80°C. Condition 3: Overnight storage at 4°C. AA, asiatic acid; AS, asiaticoside; CA, caffeic acid; DHCA, dihydrocaffeic acid; DHFA, dihydroferulic acid; Di-CQAs, dicaffeoylquinic acids (1,3-dicaffeoylquinic acid, 1,4-dicaffeoylquinic acid, 1,5-dicaffeoylquinic acid, isochlorogenic acid A, isochlorogenic acid B, isochlorogenic acid C); FA, ferulic acid; HPP, 3-(3-hydroxyphenyl)propionic acid; IFA, isoferulic acid; MA, madecassic acid; Mono-CQAs, monocaffeoylquinic acids (chlorogenic acid, cryptochlorogenic acid, neochlorogenic acid); MS, madecassoside. Horizontal lines show ±15% of the nominal concentration.

*Plasma stability:* Stability is presented as the percentage of the nominal amount added prior to the sample being subjected to three different storage conditions at three concentrations (Figure 2). In human plasma, all analytes showed good stability (±15% of nominal concentration) during long-term storage at -80°C (condition 1) for high (87%–110%) and medium concentrations (99%–113%), except DHCA (medium concentration 75%) (Figure 2). At low concentrations, all analytes except DHCA (75%), DHFA (81%), FA (129%), and the mono-CQAs (219%) showed good stability (94%–111%). The mono-CQAs showed the largest increase at the low-concentration during long-term storage potentially arising from the breakdown of di-CQAs. During freeze-thaw (condition 2), all but DHCA (75%) showed good stability at high concentrations (85%–105%) (Figure 2). At medium concentrations, all analytes but DHCA (51%) and IFA (117%) showed good stability (89%–113%). At low concentrations, only MA (100%), the di-CQAs (100%), DHFA (97%), CA (108%), and AA (111%) showed good stability during freeze-thaw. When stored at 4°C (condition 3), most analytes were stable (85%–115% of nominal concentration) except for DHCA (at medium and high concentrations), mono- and di-CQAs (at low concentrations) and CA at low and medium concentrations (Figure 2). At medium but not low concentration, DHCA showed substantial degradation to 23% of the initial analyte, as did other compounds at low concentrations: mono-CQAs (65%), the di-CQAs (65%), and CA (63%). CA also showed degradation at the medium concentration (75%). Where analytes showed apparent increases in amount (>115%), this could have been due to degradation of precursors (e.g., di-CQAs) breaking down to mono-CQAs or small volume changes due to evaporation during refrigeration.

Intraday stability in the autosampler was assessed by analyzing the linear equations for participant calibration curves from the PK study (Supplementary Table S2). The slope for a given participant changes only slightly for calibration curves for visit 1 and visit 2 (visit 2 slope 85%–116% of visit 1 slope). Calibration curves for the two visits were analyzed in the same chromatographic run; visit 2 samples were run later in the analysis sequence.

**FIGURE 3:**
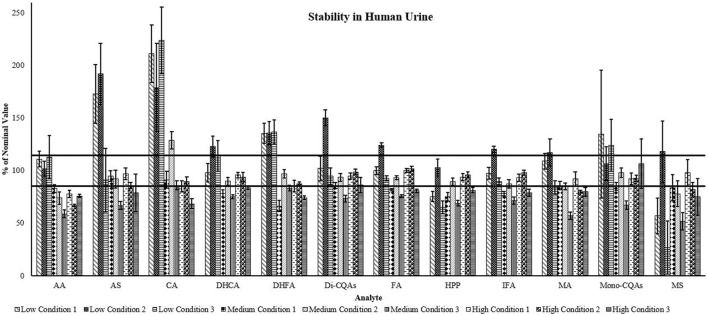
Triterpene and caffeoylquinic acid stability of three different concentrations in human urine. Data are presented as percent of nominal concentration ±SEM (*n* = 5). Low: AA 3 ng/mL, MA 3 ng/mL, AS 6 ng/mL, MS 6 ng/mL, HPP, DHFA, and CQA metabolites 5 ng/mL, CQAs (each) 0.5 ng/mL; Medium: AA 15 ng/mL, MA 15 ng/mL, AS 30 ng/mL, MS 30 ng/mL, HPP, DHFA, and CQA metabolites 25 ng/mL, CQAs (each) 2.5 ng/mL; High: AA 30 ng/mL, MA 30 ng/mL, AS 60 ng/mL, MS 60 ng/mL, HPP, DHFA, and CQA metabolites 50 ng/mL, CQAs (each) 5 ng/mL. Condition 1: Long-term storage at −80°C. Condition 2: Three rounds of freeze thaw and storage at −80°C. Condition 3: Overnight storage at 4°C. AA, asiatic acid; AS, asiaticoside; CA, caffeic acid; DHCA, dihydrocaffeic acid; DHFA, dihydroferulic acid; Di-CQAs, dicaffeoylquinic acids (1,3-dicaffeoylquinic acid, 1,4-dicaffeoylquinic acid, 1,5-dicaffeoylquinic acid, isochlorogenic acid A, isochlorogenic acid B, isochlorogenic acid C); FA, ferulic acid; HPP, 3-(3-hydroxyphenyl)propionic acid; IFA, isoferulic acid; MA, madecassic acid; Mono-CQAs, monocaffeoylquinic acids (chlorogenic acid, cryptochlorogenic acid, neochlorogenic acid); MS, madecassoside. Horizontal lines show ±15% of the nominal concentration.

*Urine stability:* In human urine, all analytes showed good stability at high concentrations (85%–115%) during long-term storage at −80°C (condition 1), except for AA (78%) and IFA (84%) (Figure 3). At medium concentrations, only AA (83%), DHFA (67%), HPP (81%), and MS (84%) showed degradation with the remaining analytes showing stability (85%–95%). However, at low concentrations AS (173%), CA (211%), and the mono-CQAs (157%) showed increases in concentration, while the remaining analytes remained within the acceptable range or decreased (DHCA (70%), DHFA (79%), FA (81%), HPP (48%), and MS (57%)) during storage. When subjected to freeze-thaw (condition 2), AA (67%), MA (80%), and MS (82%) showed degradation, while the remaining analytes were stable at high concentrations (86%–102%) (Figure 3). At medium concentrations, AA (74%), DHFA (84%), IFA (80%), and MS (78%) showed degradation, CA showed an increase to 129%, and the remining analytes showed stability (85%–92%). At low concentrations, all analytes had an increase from the starting amount when freeze-thawed (except HPP (96%)), of which only AA, IFA, and the mono-CQAs were less than 115% of the nominal concentration. For high concentration samples, overnight storage at 4°C (condition 3) resulted in a decrease in stability for all analytes except the mono-CQAs (101%), while at medium concentrations all analytes showed degradation except CA (86%) (Figure 3). At low concentrations, CA (224%) showed an increase potentially due to the breakdown of CQAs. AA (113%), AS (91%), DHCA (106%), and the mono-CQAs (100%) remained stable, and the remining analytes showed degradation.

### 3.2 Pharmacokinetic study

*Participant baseline characteristics:* The demographics and characteristics of the participants are summarized in Table 6. Participants were Caucasian, an average of 71 ± 5.5 years of age, and had a body mass index of 28.5 ± 1.5 kg/m^2^. All of the participants (four male and four female) received both doses of CAP. One participant was unable to complete the second study visit due to difficulties with maintaining venous access for sample collection (Figure 1), therefore only seven subjects were included in the analyses to reduce the potential for variation from an incomplete data set.

**TABLE 6:** Subject demographic and baseline characteristics.

Demographic data	Baseline	2 g CAW	4 g CAW
Gender, % (n)			
Female	50 (*n* = 4)	50 (*n* = 4)	50 (*n* = 4)
Male	50 (*n* = 4)	(*n* = 3)	50 (*n* = 4)
Age (years)^a^	71 [5.5]		
Body mass index (kg/m^2^)^b^	28.5 ± 1.5	28.4 ± 1.3	28 ± 1.4
Systolic blood pressure (mmHg)^b^	125 ± 4	119 ± 5	119 ± 4
Diastolic blood pressure (mmHg)^b^	78 ± 2	74 ± 3	69 ± 1
Body temperature (°C)^b^	98.3 ± 0.2	98.15 ± 0.1	98.1 ± 0.1
Heart rate (bpm)^b^	67 ± 4	69 ± 4	64 ± 3
Race, % (n)			
American Indian/Alaska Native	0	0	0
Asian	0	0	0
Black or African American	0	0	0
Native Hawaiian or Other Pacific Islander	0	0	0
Caucasian/white	100 (*n* = 8)	100 (*n* = 8)	100 (*n* = 8)
Clinical laboratory screening^b^			
White blood cell (10^3^/L)	6.7 ± 0.4	NM	NM
Red blood cell (10^6^/L)	5.0 ± 0.2	NM	NM
Hemoglobin (g/dL)	15.3 ± 0.5	NM	NM
Hematocrit (%)	46.4 ± 1.4ˆ	NM	NM
Platelet (10^3^/L)	233.9 ± 7.5	NM	NM
Blood glucose (mg/dL)	90.6 ± 4.3	NM	NM
Blood urea nitrogen (mg/dL)	15.8 ± 1	NM	NM
Creatinine (mg/dL)	0.7 ± 0.6	NM	NM
Total bilirubin (mg/dL)	0.5 ± 0.03	NM	NM
Aspartate aminotransferase (U/L)	24.6 ± 0.7	NM	NM
Alanine aminotransferase (U/L)	30.1 ± 2.5	NM	NM
Total protein (g/dL)	7.4 ± 0.1	NM	NM
Albumin (g/dL)	4.0 ± 0.1	NM	NM
Sodium (mmol/L)	140.5 ± 0.5	NM	NM
Chloride (mmol/L)	106.5 ± 0.5	NM	NM
Potassium (mmol/L)	4.0 ± 0.1	NM	NM
Total CO2 (mmol/L)	28.9 ± 0.5	NM	NM
Calcium (mg/dL)	9.2 ± 0.1	NM	NM
Anion gap	5.3 ± 0.5ˆ	NM	NM

^a^ Data are expressed as median [IQR].

^b^ Data are expressed as mean ± SEM, (*n* = 8). ˆ = outside of reference range but deemed not clinically significant. NM, not measured.

**FIGURE 4:**
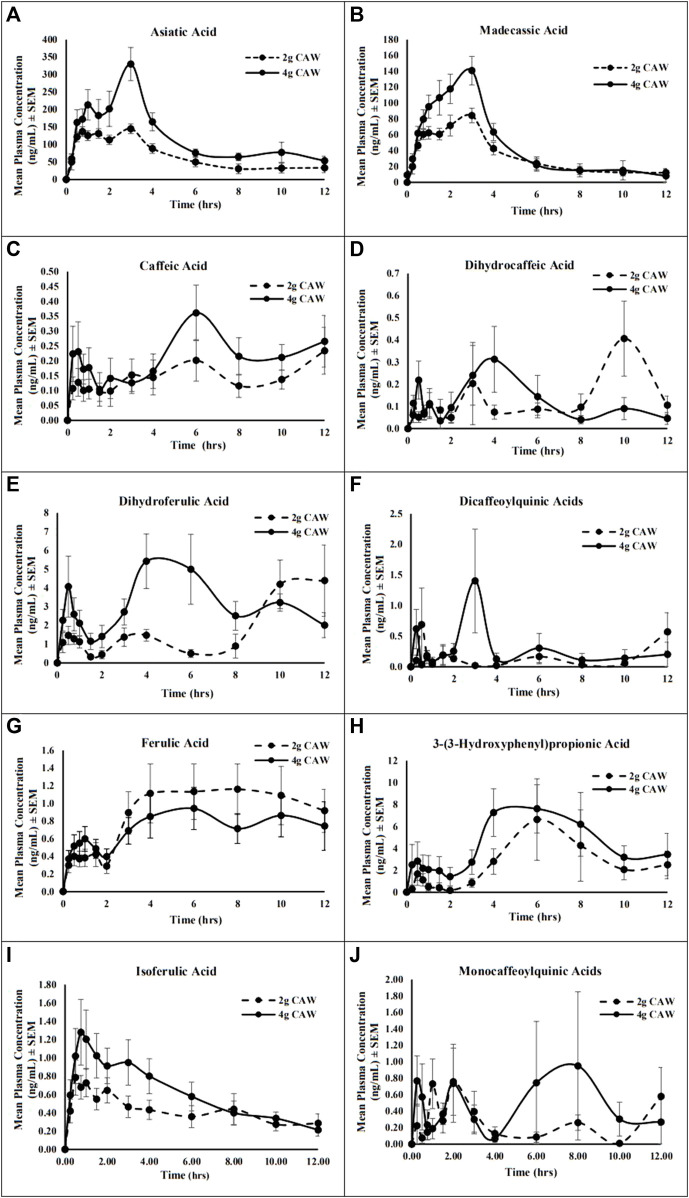
Mean plasma concentration-time profile of the triterpene aglycones, caffeoylquinic acids (CQAs), and metabolites from a *C. asiatica* water extract product (CAP) after a single oral administration of 2 or 4 g doses in healthy older adults. **(A)** asiatic acid; **(B)** madecassic acid; **(C)** caffeic acid; **(D)** dihydrocaffeic acid; **(E)** dihydroferulic acid; **(F)** dicaffeoylquinic acids; **(G)** ferulic acid; **(H)** 3-(3-hydroxyphenyl)propionic acid, **(I)** isoferulic acid; **(J)** monocaffeoylquinic acids. Data are presented as mean ± SEM (*n* = 7).

*Pharmacokinetic profiles:* For AA and MA, the PK parameters calculated using Excel PK-Solver software are presented in Table 7 and the mean PK profiles are presented as plasma concentration-time curves (Figure 4). AS and MS were not detected during analysis. Maximum plasma concentrations (C_max_) of AA (174–372 ng/mL) and MA (107–197 ng/mL) occurred at 2.2–2.8 h (T_max_) after CAP administration (Table 7). There was a significant two-fold difference between the C_max_ of the two doses for both AA (T = −4.76, *p* = 0.003) and MA (T = −3.07, *p* = 0.02). There was an approximate 2-fold difference in oral absorption (AUC_0-12_) between the doses; however, it only reached significance for AA (T = −4.01, *p* = 0.01). To account for the possibility of body mass index (BMI) or biological sex impacting maximum plasma concentrations, we assessed for correlations between BMI and C_max_ and statistical differences between biological sexes. There was no observed correlation for body mass (data not shown), and no significant difference in C_max_ between male and female participants for both AA and MA and both doses (2 g CAP: AA: T = −0.91, *p* = 0.21, MA: T = 1.94, *p* = 0.06; 4 g CAP: AA: T = 4.01, *p* = 0.2, MA: T = 1.8, *p* = 0.07). The PK curves demonstrate a distinct bimodal distribution with an initial spike in plasma analyte concentration around 1 h before reaching the T_max_ (Figure 4). For both analytes, there is a flattening of the curve at approximately six hours indicating the transition from tissue distribution to elimination. This transition is slightly less pronounced for AA.

**TABLE 7:** Pharmacokinetic parameters of orally administered *C. asiatica* water extract product (CAP) in plasma from healthy older adults.

Pharmacokinetic parameter	Analyte	2 g CAW (*n* = 7)	4 g CAW (*n* = 7)	*p*-value
C_max_ (ng/mL)	AA	174 (22)	372 (53)	0.003*
	CA	0.3 (0.1)^	0.5 (0.1)^	0.14
	DHCA	0.4 (0.1)^	0.5 (0.1)^	0.47
	DHFA	9.3 (4.4)	8.1 (2.9)	0.80
	Di-CQAs	1.2 (0.6)^	1.9 (0.8)^	0.47
	FA	1.5 (0.3)	1.5 (0.6)	0.42
	HPP	5.8 (3.0)	10.6 (2.9)	0.14
	IFA	1.5 (0.8)	1.5 (0.4)	0.96
	MA	107 (18)	197 (43)	0.02*
	Mono-CQAs	1.6 (0.5)^	4.2 (1.4)^	0.15
T_max_ (hr)	AA	2.2 (0.4)	2.8 (0.2)	0.11
	CA	5.3 (1.9)	4.5 (1.6)	0.79
	DHCA	6.1 (1.7)	3.2 (1.3)	0.30
	DHFA	5.9 (1.6)	5.7 (1.5)	0.95
	Di-CQAs	3.7 (1.9)	4.3 (1.7)	0.85
	FA	5.9 (1.3)	7.1 (1.2)	0.55
	HPP	7.4 (1.5)	5.7 (0.7)	0.37
	IFA	2.4 (1.0)	1.0 (0.32)	0.38
	MA	2.4 (0.3)	2.7 (0.2)	0.59
	Mono-CQAs	1.2 (0.4)	3.42 (1.2)	0.12
T_1/2_ (hr)	AA	4.1 (0.7)	4.9 (1.3)	0.64
	CA	8.4 (3.0)	10.3 (3.7)	0.46
	DHCA	1.8 (1.2)	1.9 (0.8)	0.98
	DHFA	7.1 (1.7)	6.6 (3.8)	0.48
	Di-CQAs	2.0 (1.2)	2.6 (1.8)	0.42
	FA	14.1 (3.9)	2.9 (1.3)	0.23
	HPP	5.0 (2.0)	2.6 (0.5)	0.32
	IFA	7.0 (2.4)	3.5 (0.5)	0.35
	MA	9.2 (3.7)	6.7 (0.8)	0.26
	Mono-CQAs	3.4 (1.1)	1.7 (1.1)	0.47
AUC_(0-12)_ (ng/mL*hr)	AA	48,755 (7,152)	91,330 (17,723)	0.01*
	CA	1.8 (0.5)	2.4 (0.6)	0.30
	DHCA	1.7 (0.6)	1.9 (0.5)	0.82
	DHFA	35.6 (18.6)	41.0 (12.7)	0.77
	Di-CQAs	3.4 (1.8)	5.0 (1.5)	0.36
	FA	11.3 (2.9)	9.9 (2.8)	0.18
	HPP	56.8 (31.7)	50.2 (15.1)	0.12
	IFA	5.3 (1.6)	7.4 (1.6)	0.79
	MA	26,771 (3,585)	48,373 (12,476)	0.11
	Mono-CQAs	6.2 (2.4)	10.1 (3.9)	0.34
Total urinary excretion (µg; 12 hr)	AA	Not detected	Not detected	
	AS	20.68 (6.7)	18.6 (4.5)	1.78
	CA	42.8 (11.6)	48.7 (9.3)	0.44
	DHCA	26.8 (10.0)	24.8 (6.1)	0.4
	DHFA	225.5 (83.5)	225.1 (94.8)	0.4
	Di-CQAs	Not detected	Not detected	
	FA	146.8 (33.5)	137.4 (24.6)	0.4
	HPP	223.4 (76.7)	247.9 (73.6)	0.4
	IFA	65.9 (23.1)	225.1 (15.9)	0.4
	MA	Not detected	Not detected	
	MS	Not detected	Not detected	
	Mono-CQAs	21.3 (8.6)	19.3 (2.6)	0.32

Pharmacokinetic parameters were calculated using a non-compartmental analysis of plasma concentration *versus* time data using Excel software PK-solver (version 2.0). Two-sided paired t-tests were used to compare pharmacokinetic and clearance parameters between the 2 and 4 g doses. CAW = *C. asiatica* water extract; C_max_ = maximum plasma concentration; T_max_ = time to reach C_max_; AUC_0–12_ = area under the plasma concentration–time curve from time zero to time 12 h; T_1/2_ = elimination half-life; AA, asiatic acid; AS, asiaticoside; CA, caffeic acid; DHCA, dihydrocaffeic acid; DHFA, dihydroferulic acid; di-CQAs, di-caffeoylquinic acids; FA, ferulic acid; HPP, 3-(3-hydroxyphenyl)propionic acid; IFA, isoferulic acid; MA, madecassic acid; MS, madecassoside; mono-CQAs, mono-caffeoylquinic acids. Data are expressed as mean (SEM); *p < 0.05 for significant differences. ^ = values falling below the lower limit of quantitation.

For the mono-CQAs and di-CQAS, as well as their putative metabolites, the PK parameters calculated are presented in Table 7 and the mean PK profiles are represented as plasma concentration-time curves in Figure 4. C_max_ for all CQAs and metabolites (0.3–10.6 ng/mL) occurred over a wide range of time (T_max_ = 1.2–7.4 h) after CAP administration (Table 7). There was no significant difference between the C_max_ of the 2 and 4 g doses for any of the analytes. There was a notable difference in the oral absorption (AUC_0-12_) between the doses for all CQAs and metabolites; however, none reached significance. When assessed for correlations between BMI and C_max_, and for statistical differences between biological sexes, there was no observed correlation for BMI (data not shown). DHCA (T = 2.25, *p* = 0.03), HPP (T = −2.04, *p* = 0.05), and the mono-CQAs (T = −0.04, *p* = 0.05) demonstrated a significant difference in C_max_ between male and female participants for the 2 g dose. However, the difference did not reach significance for the 4 g dose (DHCA: T = 0.35, *p* = 0.37, HPP: T = −1.33, *p* = 0.12, mono-CQAS: 4 g T = −1.19, *p* = 0.14). All other CQA analytes did not reach significance for biological sex differences for either dose (2 g CAP: CA: T = 0.81, *p* = 0.22, DHFA: T = 1.36, *p* = 0.12, di-CQAs: T = −0.29, *p* = 0.39, FA: T = −0.67, *p* = 0.27, IFA: T = −0.87, *p* = 0.21; 4 g CAP: CA: T = 0.13, *p* = 0.45, DHFA: T = 1.29, *p* = 0.13, di-CQAs: T = −0.25, *p* = 0.41, FA: T = −0.96, *p* = 0.2, IFA: T = 1.75, *p* = 0.07). The PK curves demonstrate more variability than those observed for the TTs, with a smaller initial peak and a delayed larger peak (Figure 4).

*Renal excretion:* Total amounts of analyte for the TTs, CQAs, and CQA metabolites excreted over the 12-h post-administration period are presented in Table 7. AS was the only TT detected in the urine; however, there was no significant difference in the amount of analyte excreted between the two doses (T = 0.35, *p* = 1.74). The remaining TTs were not detected. All of the CQAs and metabolites, with the exception of the di-CQAs, were detected in the urine at both doses, but none were found to have a significant dose difference.

*Safety and tolerability:* CAP was largely well tolerated at both doses with no immediate or delayed significant adverse events reported or identified by ECG (Table 8). No-one withdrew from the study due to the study intervention. The only reported adverse events were mild to moderate gastrointestinal symptoms (i.e., diarrhea, constipation, nausea, or belching), increased thirst, decreased appetite, headache, and changes in urine color that resolved within 24 h and may have been related to the study intervention or the low phytochemical diet.

**TABLE 8:** Summary of adverse events of *C. asiatica* water extract product (CAP) after acute administration.

Adverse event	2 g CAW*	4 g CAW*	Relation to intervention
Psychological/General			
Excessive Sleep	1/8 (12.5%) Mild		Not Related
Disappointment	1/8 (12.5%) Mild		Not Related
Anxiety	1/8 (12.5%) Mild		Not Related
Boredom		1/7 (14.3%) Mild	Not Related
Weakness/fatigue		1/7 (14.3%) Mild	Not Related
Neurological/Muscle			
Musculoskeletal pain or stiffness	3/8 (37.5%) Mild	1/7 (14.3%) Mild	Not Related
Tremor	1/8 (12.5%) Mild		Not Related
Headache	1/8 (12.5%) Mild	2/7 (28.6%) Mild	Possibly Related
Head, Eyes, Ears, Nose, and Throat			
Vertigo		1/7 (14.3%) Mild	Not Related
Ringing in the ears/tinnitus		1/7 (14.3%) Mild	Not Related
Blurred vision		1/7 (14.3%) Mild	Not Related
Cardiopulmonary			
Hypotension	1/8 (12.5%) Moderate		Not Related
Fainting/dizziness	3/8 (37.5%) Moderate		Not Related
Coughing	1/8 (12.5%) Mild		Not Related
Peripheral edema or swelling		1/7 (14.3%) Moderate	Not Related
Gastrointestinal			
Diarrhea	1/8 (12.5%) Moderate	1/7 (14.3%) Mild	Possibly Related
Gas/indigestion	2/8 (25%) Mild	1/7 (14.3%) Mild	Possibly Related
Constipation	1/8 (12.5%) Mild	1/7 (14.3%) Moderate	Possibly Related
Increased thirst	2/8 (25%) Mild		Possibly Related
Dry throat	1/8 (12.5%) Mild		Not Related
Dry mouth	1/8 (12.5%) Mild		Possibly Related
Abnormal or metallic taste in mouth	2/8 (25%) Mild	4/7 (57%) Mild	Related
Abdominal pain/cramps	1/8 (12.5%) Mild	1/7 (14.3%) Mild	Possibly Related
Decreased appetite		1/7 (14.3%) Mild	Possibly Related
Nausea		1/7 (14.3%) Mild	Possibly Related
Wet burps with grassy flavor	1/8 (12.5%) Mild		Related
Genitourinary			
Increased urination		1/7 (14.3%) Mild	Not Related
Urine color change	1/8 (12.5%) Mild	2/7 (28.6%) Mild	Possibly Related
Skin			
Itchiness/dryness	1/8 (12.5%) Mild	1/7 (14.3%) Mild	Not Related
Color changes, paling, yellow, other	3/8 (37.5%) Mild		Not Related
Redness	4/8 (50%) Mild	1/7 (14.3%) Mild	Not Related
Rash/hives	1/8 (12.5%) Mild	1/7 (14.3%) Mild	Not Related
Bruising		1/7 (14.3%) Mild	Not Related
Skin puffiness/tissue fluid	1/8 (12.5%) Mild		Not Related
Ecchymosis, indurate	1/8 (12.5%) Mild		Not Related
Whole Body			
Weight gain	1/8 Mild	1/7 (14.3%) Mild	Not Related
Weight loss	1/8 Mild		Not Related

Each event was ranked on a scale of 0–5 based upon the following criteria: 0, absent; 1, mild; 2, moderate; 3, severe; 4, life-threatening; and 5, fatal.

* *Centella asiatica* water extract = CAW.

## 4 Discussion

Validation plays an important role in determining the reproducibility of a method, suitability of an analytical procedure, and application to clinical trials. It is especially important to validate methods for all analytes of interest in different matrices, as not all may react the same in each matrix under different conditions. In this study, we followed FDA guidance (Food and Drug Administration, 2022) to assess the linearity, precision, accuracy, thermal stability, and recovery of key *C. asiatica* analytes in both human plasma and urine. In the development of this method, we used HPLC-MS/MS technology similar to its prior use for the quantification of TTs from *C. asiatica* in human matrices (Songvut et al., 2019) and CQAs in *C. asiatica* mixtures (Yang et al., 2023). Using this technology allows for more accurate, sensitive, and selective quantification than methodologies used in earlier *C. asiatica* PK studies including liquid chromatography coupled to UV/vis spectrophotometry (Grimaldi et al., 1990) and gas chromatography-mass spectrometry (Rush et al., 1993). We also expanded the PK study to examine CQA compounds from *C. asiatica* and their putative metabolites. While the concentrations of these compounds are much lower than those of the TTs, we evaluated their oral absorption and PK parameters due to promising preclinical *in vitro* and *in vivo* studies demonstrating neurological bioactivity of these compounds relevant to aging and cognition at concentrations equivalent to their presence in active *C. asiatica* extracts (Gray et al., 2014; Matthews et al., 2020).

### 4.1 Validation

The goal of this study was to validate a clean-up and analytical method that would be able to capture both TTs and CQAs in human biological matrices. Our method exhibited good linearity, with the exception of the TT glycosides (Table 3). However, these compounds were not detected in human plasma in the PK study. The analytes of interest exhibited high accuracy and high precision in human plasma and urine at medium and high concentrations according to the FDA guidelines (Tables 4, 5). There was decreased accuracy and precision for some compounds at lower concentrations in both matrices (di-CQAs, mono-CQAs, AS, and MS) necessitating the development of more sensitive methodologies. Part of the challenge with developing a methodology to detect both metabolites and parent compounds is the inherent degradation and *in-vivo* bioconversion that occurs. This could explain why the linearity, accuracy, and precision of the parent glycosides decreased and the recovery of the aglycones increased to greater than 100% when subjected to sample work-up and analysis. Further analysis and adjustment of the quantitation range is needed to develop a validated method for the parent compounds that meets the FDA’s requirements.

The thermal stability of the analytes in human plasma subjected to different conditions was decreased when stored at 4°C compared to −80°C for CA, the di-CQAs, and the mono-CQAs. It was ≥100% of the original amount for many of the metabolites potentially due to degradation of parent compounds (Figure 2). At higher concentrations, there was greater stability at -80°C and more degradation noted during repeat freeze-thaw and storage at 4°C. This suggests that the analytes are most stable at higher concentrations, and when frozen and stored at −80°C; however, samples should be processed and analyzed after the first thaw, otherwise there is the risk of decreased levels upon refreezing. Time spent processing samples at 4°C should be minimized (typically this would be 4–6 h).

The stability of the analytes in human urine was decreased when compared to the plasma (Figure 3). At 4°C, there was degradation of all analytes at medium concentrations, and degradation of many except for MA, AA, CA, DHCA, and the mono-CQAs at low concentrations. There was a substantial increase in mono-CQAs, AS, and CA with long-term storage, and a large increase in most analytes during repeat freeze-thaw at lower concentrations. This suggests that these analytes are more accurately detected when present in higher concentrations and stored at −80°C when using urine as the biological matrix.

### 4.2 Pharmacokinetic trial

This study provides valuable information about the oral absorption and pharmacokinetics of *C. asiatica* TTs and CQAs in human plasma following the administration of a product (CAP) made from an aqueous extract of the aerial parts of the plant. The use of an aqueous extract product is more consistent with ethnopharmacological prescribing and accounts for potential interactions and synergy between compounds within the plant during the extraction processes. Additional components in the plant matrix not assessed in this study may also have a bio-enhancing effect. The potentially unknown interaction of compounds within the plant matrix, further complicated by *in vivo* variations in gut microbiomes and metabolism, underscores the complexity of clinical botanical drug research. The use of a whole herb product moves away from constituent isolation, the norm in pharmaceutical development, and accounts for the potential therapeutic benefit of multiple compounds. Previous studies used either isolated AA and AS compounds (Rush et al., 1993), only TTs like the total triterpenic fraction of *C. asiatica* (TTFCA: AS, AA and MA) (Grimaldi et al., 1990), or a product enriched with triterpenes ECa 233 (AA, AS, MA, and MS), and do not consider the potential impact of using isolated constituents in place of a whole herb product on the PK in humans. Additionally, these studies (excluding the ECa 233 study) only look at the PK of AS and AA and do not consider MS and MA at all. This is the first study in healthy older adults to consider the potential additional active compounds from *C. asiatica* (the CQAs) shown to have bioactivity in preclinical *in vitro* and *in vivo* studies (Gray et al., 2014; Matthews et al., 2019; Matthews et al., 2020).

The participants for this study are also distinct in that they are older adults (65–85 years) and are more representative of the age group that experiences cognitive decline. Prior studies have used participants that were healthy younger individuals (18–50 years) (Grimaldi et al., 1990; Rush et al., 1993; Songvut et al., 2021). Age is an important factor in PK studies as altered gastrointestinal transit time and absorption due to enzymatic changes, phase I metabolism in the liver, and changes in the excretory rate due to renal impairment are all factors that impact older adults (Mangoni and Jackson, 2004). Polypharmacy (the prescription of many different pharmaceutical or natural product agents simultaneously) is another factor to consider, as populations of advanced age are also more likely to have chronic conditions that are treated with pharmaceuticals (Hughes, 1998). This could lead to a potential interaction between other drugs and the metabolites of interest, thereby delaying or preventing absorption and/or slowing the rate of excretion influencing the overall PK profile (Shi and Klotz, 2011).

The parent TT compounds, AS and MS, were below the LoD in the plasma of PK trial participants (Table 7). Their corresponding aglycones, AA and MA, were readily detected even though they were much less abundant in CAP (Wright et al., 2022). This is similar to prior human PK studies in which pure glycosidic TT compounds and purified TT mixtures were administered and only the aglycones were detected (Grimaldi et al., 1990; Rush et al., 1993; Lou et al., 2018; Songvut et al., 2019). In a more recent formulation of a *C. asiatica* commercial extract (ECa 233), a solubilizing agent improved the absorption of the glycosides, but the amount detected remained substantially less than the aglycones (Songvut et al., 2021). This supports that the aglycones are the predominant TT absorbed by humans most likely due to *in vivo* bioconversion via hydrolysis by stomach acid, digestive enzymes, or β-glycosidase-producing bacteria in the human gastrointestinal microbiome (Grimaldi et al., 1990; Kobashi and Akao, 1997). AA is generally considered the most biologically active compound in *C. asiatica* due to its protective effects against amyloid induced neurotoxicity and free radical scavenging ability (Gray et al., 2018a; Songvut et al., 2019). Yet, MA has also shown neuroprotective anti-inflammatory effects in ischemic conditions, improved synaptic plasticity, and activity in the D-galactose model of brain aging (Gray et al., 2018a). Since, MA was detected in plasma, it should not be discounted as a potentially important bioactive.

The C_max_ for AA (174, 372 ng/mL) was greater than MA (107, 197 ng/mL) at both doses, likely due to a greater starting amount of AS than MS in CAP (Wright et al., 2021). These C_max_ values are higher than those observed in a recent study by our group of 2 and 4 g CAP in older adults on cholinesterase inhibitors (C_max_ of AA: 123, 259 ng/mL, MA: 38, 63 ng/mL, respectively) (Wright et al., 2022) and much higher than those observed in participants given 250 mg and 500 mg doses of ECa 233 (C_max_ of AA: 38, 84 ng/mL; MA: 40, 52 ng/mL, respectively) (Songvut et al., 2019; Songvut et al., 2021). Due to the lower levels of these compounds found in older adults on cholinesterase inhibitors, it could be proposed that these medications may prevent absorption or increase metabolism of CAP TTs. Alternatively, persons with cognitive impairment may have an altered gastrointestinal flora or delayed absorption due to the state of their disease (Nagpal et al., 2020; Doifode et al., 2021; Yıldırım et al., 2022). To compare the results in this study to those using ECa 233, we calculated the moles per dose of each analyte using values for AS and MS in ECa 233 from a rat PK study (80% triterpenoid glycosides; 53.1% MS, 32.3% AS, and less than 1% AA and MA) (Anukunwithaya et al., 2017). Although the doses for AA were similar in CAP (2 g: 73.6 μmol, 4 g: 134.3 μmol) and ECa 233 (250 mg: 84.0 μmol, 500 mg: 168.4 μmol), there was a large difference between the C_max_ observed for AA in each study (CAP 2 g: 0.36 μM, 4 g: 0.76 μM; ECa 233 250 mg: 0.078 μM, ECa 233 500 mg 0.17 μM). We also see the same pattern for MA (CAP 2 g: 49.4 μmol, CAP 4 g: 97.7 μmol; ECa 233 250 mg: 136.0 μmol, ECa 233 500 mg: 272.0 μmol), with C_max_ values from CAP (2 g: 0.21 μM, 4 g: 0.39 μM) being much greater than those of ECa 233 (250 mg: 0.08 μM, 500 mg 0.10 μM), despite CAP containing less MA equivalents in each dose. This could be due to different degrees of breakdown of TT glycosides (MS and AS) to TT aglycones (AA and MA) in CAP and ECa 233 leading to different levels of AA and MA in the plasma. Also, Songvut et al., 2019 did not include a digestion step in their sample work up method to liberate the metabolites from their glucuronide and sulfate conjugates. Higher concentrations of AA and MA may have been present as glucuronides or sulfates in the plasma of participants who took ECa 233 but were not detected for this reason. MA has an additional hydroxyl group compared to AA giving a greater possibility for the formation of conjugates. The mode of administration of CAP as a drink compared to ECa 223 in capsule form may also have contributed to the difference.

We observed a near 2-fold increase in C_max_ and AUC_(0-12)_ between the doses, reaching significance for AA, and slight variability in T_max_ and T_1/2_ from 2 to 4 g, although the difference was not significant (Table 7). The slight delay in T_max_ could be due to the rate of biotransformation of AS to AA being dependent on plasma AA concentration levels (Grimaldi et al., 1990). The observed dose dependent response agrees with existing human PK studies of AA and MA (Grimaldi et al., 1990; Songvut et al., 2019; Songvut et al., 2021; Wright et al., 2022).

The plasma-concentration time curves for AA and MA show a distinct bimodal distribution with the second peak being the T_max_ (2.2–2.8 h) (Figure 4). The standard error bars indicate variability among the seven participants likely due to differences in gastrointestinal transit time, gastrointestinal microflora, absorption, metabolism, and excretion. The observed curves for AA are higher in concentration than those of MA, which is consistent with existing data (Songvut et al., 2021). This “sawtooth-like” pattern has been seen previously for both AA (Grimaldi et al., 1990; Wright et al., 2022) and MA (Leng et al., 2013; Wright et al., 2022); however, those studies in which they administered a capsular form found a more delayed T_max_ (4–4.5 h). This could be due to the need for capsule dissolution and metabolism prior to absorption. However, a more recent study of encapsulated ECa 233 in healthy adults showed that AS and MS reached T_max_ within 1–2 h, while AA and MA reached T_max_ at 1.5 h (Songvut et al., 2019). These times are more consistent with the initial peak found in this study, suggestive that the first peak represents AA or MA directly from CAP, while the second peak could be attributed to the bioconversion of AS to AA or MS to MA by gut microflora, enterohepatic circulation *in vivo*, or a combination of these factors. This is further supported by the lack of a bimodal peak distribution noted by Songvut et al., 2019, and the miniscule quantities of AA and MA present in ECa 233. It also suggests that absorption time could potentially be influenced by other components present in the CAP, as opposed to purified triterpene extracts. Another potential factor is the age difference between the participants in our study (65–85 years) and the ECa 233 study (31.3 ± 8.79 years). Older individuals are likely to have slower gastrointestinal transit time and potentially impaired renal function due to aging and/or other chronic diseases (Mangoni and Jackson, 2004). Both groups were fasted before the trial, however, only our group put the participants on a low phytochemical diet before the fasting period. Not being placed on a low phytochemical diet may not be of consequence, as the participants in the ECa 233 study had overall lower analyte plasma concentrations, which indicates that the analytes were not likely from food sources. The last factor to consider is the difference in race/ethnicity of the two groups (Thai vs. Caucasian participants) and how that may or may not influence the digestion and metabolism of the respective products.

As previously mentioned, CQAs may potentially contribute to the improvement of cognitive function based on *in-vitro* and preclinical studies using a *C. asiatica* water extract (Gray et al., 2018b; Matthews et al., 2020). Prior PK studies have focused on the *C. asiatica*-specific TTs with one human trial from our group investigating the absorption, distribution, metabolism, and excretion (ADME) of CQAs from *C. asiatica* (Wright et al., 2022)*.* Others have evaluated CQA ADME from other plant sources, such as coffee (*Coffea arabica*) (Clifford et al., 2007; Williamson et al., 2011). Our data show that the parent compounds (di-CQAs and mono-CQAs), and many of their putative metabolites (CA, DHCA, DHFA, FA, HPP, and IFA) can be detected in human plasma after oral administration of CAP (C_max_ 2 g: 0.3–9.3 ng/mL; C_max_ 4 g: 0.5–10.6 ng/mL) (Table 7) and demonstrate a time-dependent curve (Figure 4). However, many of these compounds were found to be above the LoD, but at or below the LLoQ. Therefore, these values should be interpreted with caution until more sensitive methodologies can be developed. Many of the metabolites are found in greater quantities than the parent compounds due to bioconversion. It is well documented in the literature that CQAs are metabolized into caffeic and ferulic acid derivatives by gastrointestinal microflora and Phase I and II metabolism (Wong et al., 2010; Williamson et al., 2011; Erk et al., 2012). Many of the compounds trend towards a two-fold increase between doses, with some having decreased amounts in the higher doses (Table 7). This decrease may be due to enzymatic saturation resulting in greater concentrations of parent compounds and decreased concentration of some of the metabolites when the dose reaches a certain threshold (Erk et al., 2012).

The plasma-concentration time curves for the CQA related compounds do not show a distinct bimodal pattern like the TT aglycones (Figure 4). Instead, they show a smaller initial peak, a delayed larger second peak ranging from 1–7 h and not returning back to baseline within the 12-h collection period. These findings match those seen in older adults on cholinesterase inhibitors taking *C. asiatica,* as well as prior PK studies on coffee-derived CQAs (Williamson et al., 2011; Wright et al., 2022). These previous studies also identified significant variability between subjects, as was seen in this study. This could be due to decreased stomach acidity due to age or variability in gastrointestinal transit time between subjects, as dihydrometabolites have been proposed to be formed in the more distal parts of the digestive tract (jejunum and colon) (Su et al., 2014). In addition, the level at which these compounds are being detected is at or below the LLoQ for this method making it difficult to accurately detect changes in concentration until more sensitive methods are developed and validated.

The detection of AS in the urine, and MS in a prior trial (Wright et al., 2022), suggests that minor amounts of the glycosides may be absorbed and rapidly excreted (Table 7). With the levels of the aglycones being below the LoD, it suggests that they are predominantly excreted via bile and feces. For the CQAs, the di-CQAs were not detected and the mono-CQAs were detected in small quantities; however, most of the other CQA-related analytes were detected in substantial amounts (Table 7). This result was anticipated based upon prior coffee trials in which urinary excretion analyses did not identify CQAs but did find CQA metabolites (Stalmach et al., 2009).

*C. asiatica* has widespread use as a dietary supplement and is classified as a Class 1 herb by The Botanical Safety Handbook (Botanical Safety Handbook, 1997). In this study, minor adverse events were reported, predominantly gastrointestinal upset and headache (Table 8). While these symptoms were noted in a prior trial of CAP, and could be attributed to the study intervention, they also may be due to caffeine withdrawal from the low phytochemical diet used in both trials (Wright et al., 2022). This observed tolerability, and no prior studies identifying any significant changes in clinical or laboratory measures, suggest that CAP is safe (Songvut et al., 2019; Songvut et al., 2021; Wright et al., 2022). Additional studies of repeat dosing over longer durations remain needed to determine the safety of chronic CAP use.

### 4.3 Limitations

The pharmacokinetic data obtained for the CQAs and their putative metabolites was limited by the decreased accuracy and precision at the low concentrations. While the absolute concentration of many of the CQA metabolites found in the PK study were below the LLoQ, time-dependent changes in these metabolites were observed. Values reported that fall within these lower ranges should be interpreted with caution until more sensitive methodology to accurately and precisely quantify the CQAs and metabolites at these lower concentrations are developed. The effect of hemolysis on the detection and quantification of analytes in plasma samples was not evaluated in the validation of the method. Additional investigation is needed to determine the impact of hemolysis on accurate quantification of TTs and CQAs in human plasma samples. Lastly, this study did not validate methodology to detect all compounds present within *C. asiatica* aqueous extracts (Alcázar Magaña et al., 2020), nor did it determine the PK profile of each of these compounds in humans. Future studies are needed to validate methods for detecting these compounds in human matrices if they are observed to have bioactivity through further preclinical and metabolomic study. Stored plasma and urine samples could be used to analyze the PK of these additional compounds later.

### 4.4 Conclusion

Here we present a validated method for the isolation and detection of triterpenes and caffeoylquinic acids from *C. asiatica* in human plasma and human urine and its application to a human pharmacokinetic trial in older adults. Key active compounds from an aqueous extract of *C. asiatica* are orally absorbed demonstrating dose-dependent pharmacokinetics. This study serves as the foundation for future clinical trials and the development of a new treatment option for cognitive decline in normal aging or cognitive impairment in Alzheimer’s disease that is consistent with ethnopharmacological prescribing and with broader therapeutic potential than formulas containing triterpenes alone.

## Data Availability

The raw data supporting the conclusion of this article will be made available by the authors, without undue reservation. The data are not publicly available due to HIPAA.
