# Supplementary material for: Bioanalytical method validation and application to a phase 1, double-blind, randomized pharmacokinetic trial of a standardized Centella asiatica (L.) Urban water extract product in healthy older adults
Source: Front Pharmacol. 2023 Aug 23;14:1228030. doi: 10.3389/fphar.2023.1228030 (PMC10481538; doi:10.3389/fphar.2023.1228030)
Supplement: Supplementary file 1 [file DataSheet1.docx]

Supplementary Material Bioanalytical Method Validation and Application to a Phase 1, Double-Blind, Randomized Pharmacokinetic Trial of a Standardized Centella asiatica (L.) Urban Water Extract Product in Healthy Older Adults

Kirsten M. Wright^*^, Melissa Bollen, Jason David, Bridgette Mepham, Armando Alcazar Magana, Christine McClure, Claudia S. Maier, Joseph E. Quinn, and Amala Soumyanath

*** Correspondence:**Kirsten Wright
[wrigkir@ohsu.edu](mailto:wrigkir@ohsu.edu)

# Supplementary Figures


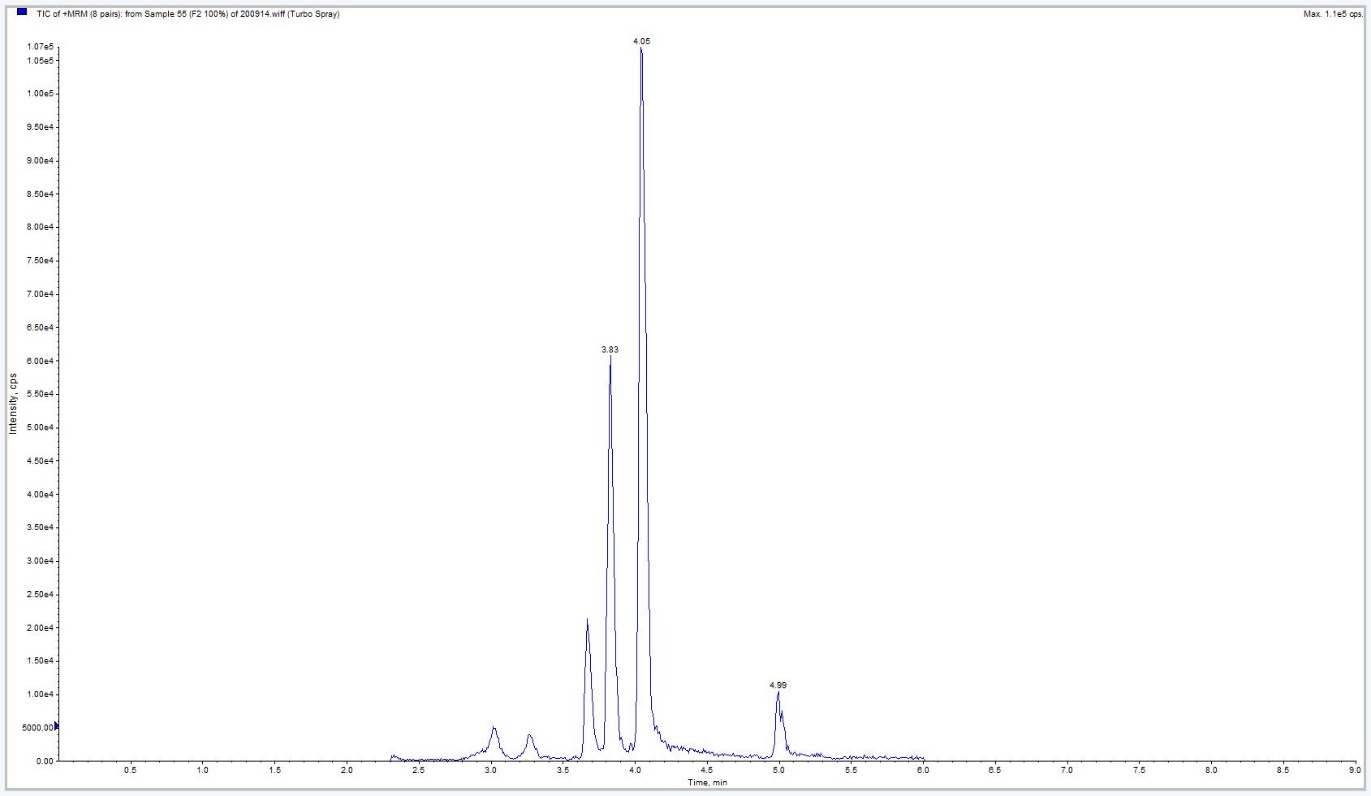


**Asiatic acid**

**Asiaticoside**

**Madecassoside**

**Madecassic acid**

1.07e5

**Supplementary Figure S1.** Total ion chromatogram (TIC) of triterpenes from *Centella asiatica* in spiked human plasma. The TIC was obtained in positive ion mode electrospray ionization using the following transitions (m/z): asiatic acid (506/453), madecassic acid (522/451), asiaticoside (976/453; 976/635), and madecassoside (992/487; 992/451).

**
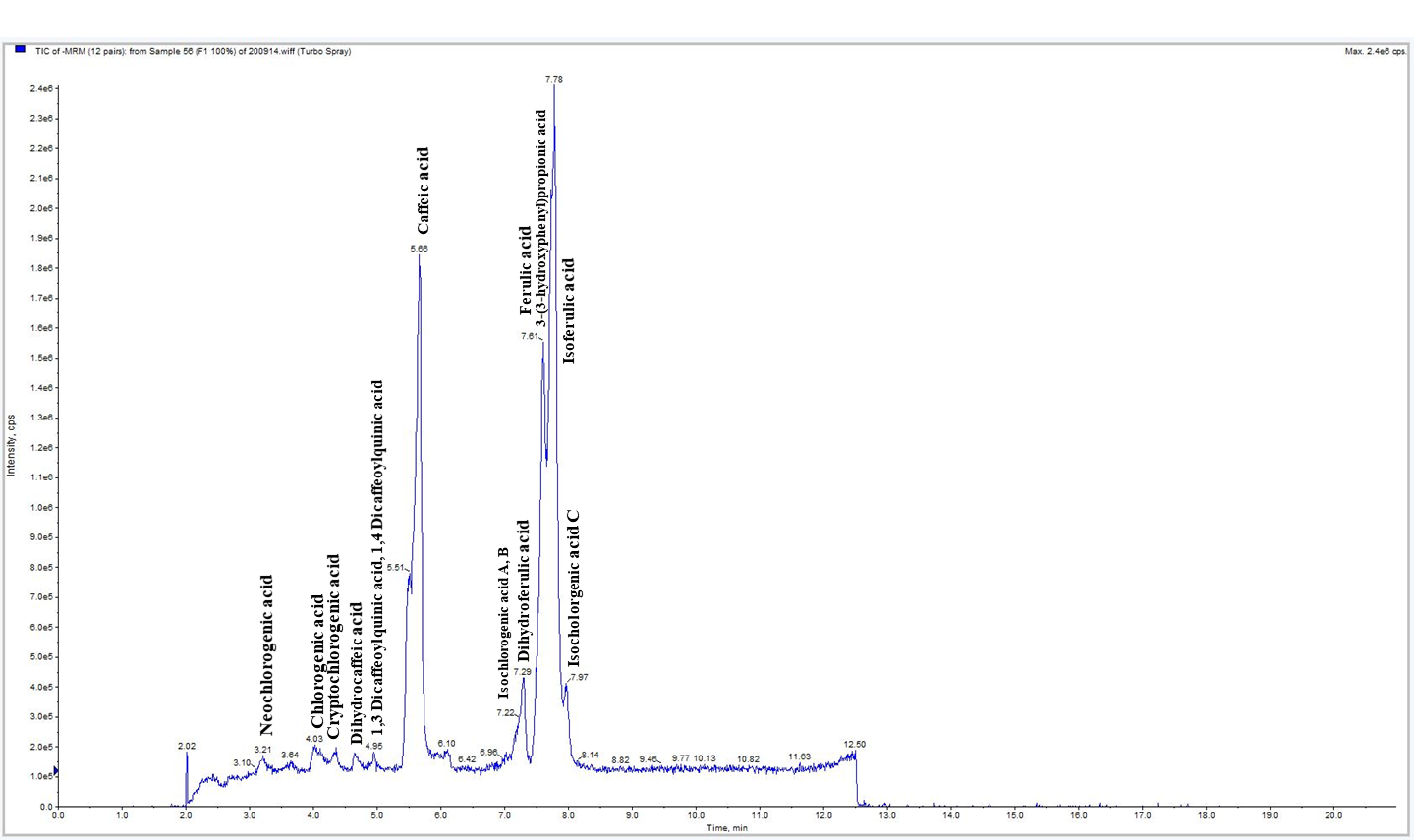
 Supplementary Figure S2.** Total ion chromatogram (TIC) of caffeoylquinic acids and metabolites from *Centella asiatica* in spiked human plasma. The TIC was obtained in negative ion mode using the following MS/MS transitions (m/z): mono-caffeoylquinic acids (353/191); di-caffeoylquinic acids (515/353; 515/191); caffeic acid (179/135); ferulic acid and isoferulic acid (193/134); dihydrocaffeic acid (181/109); dihydroferulic acid (195/136), 3-(3-hydroxyphenyl)propionic acid (165/106).

2.4e6


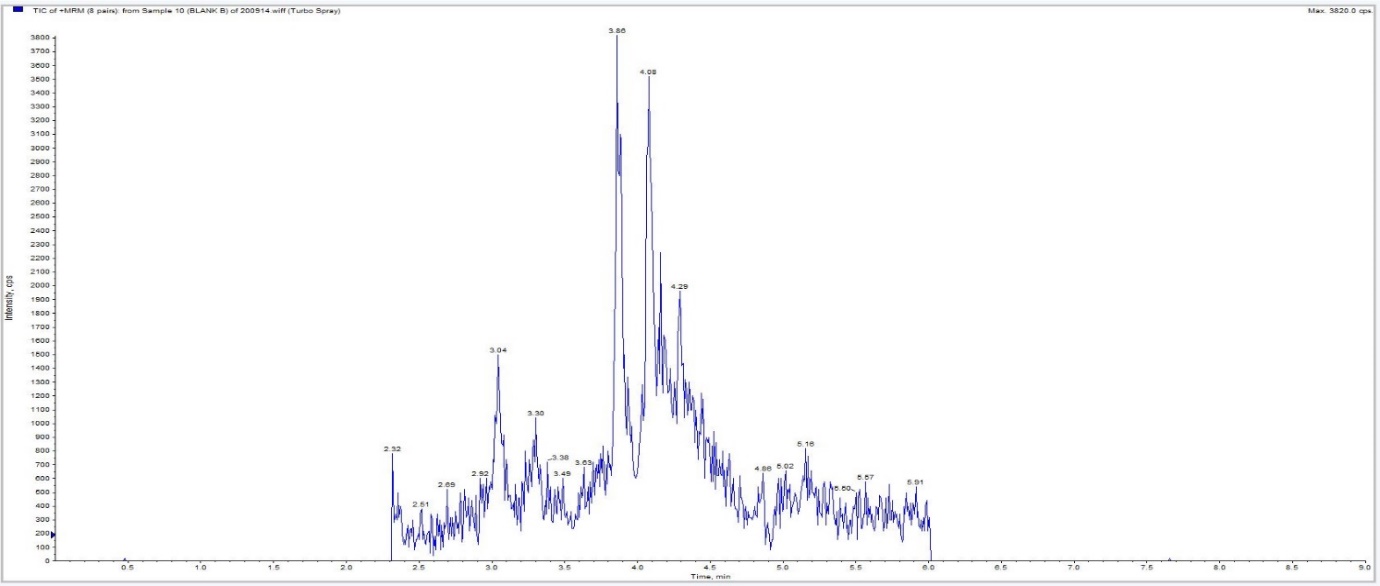


3800

**Supplementary Figure S3.** Total ion chromatogram (TIC) of blank human plasma evaluated for triterpenes. The TIC was obtained in positive ion mode.


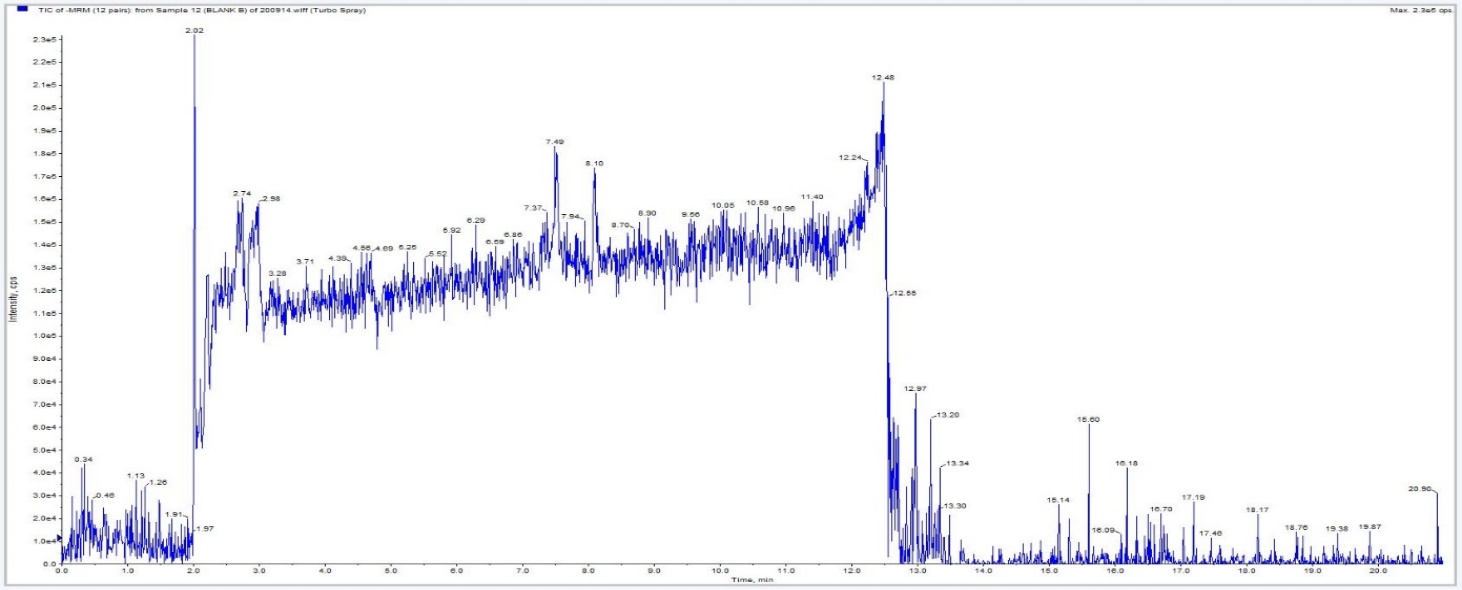


2.3e5

**Supplementary Figure S4.** Total ion chromatogram (TIC) of blank human plasma evaluated for caffeoylquinic acids. The TIC was obtained in negative ion mode.


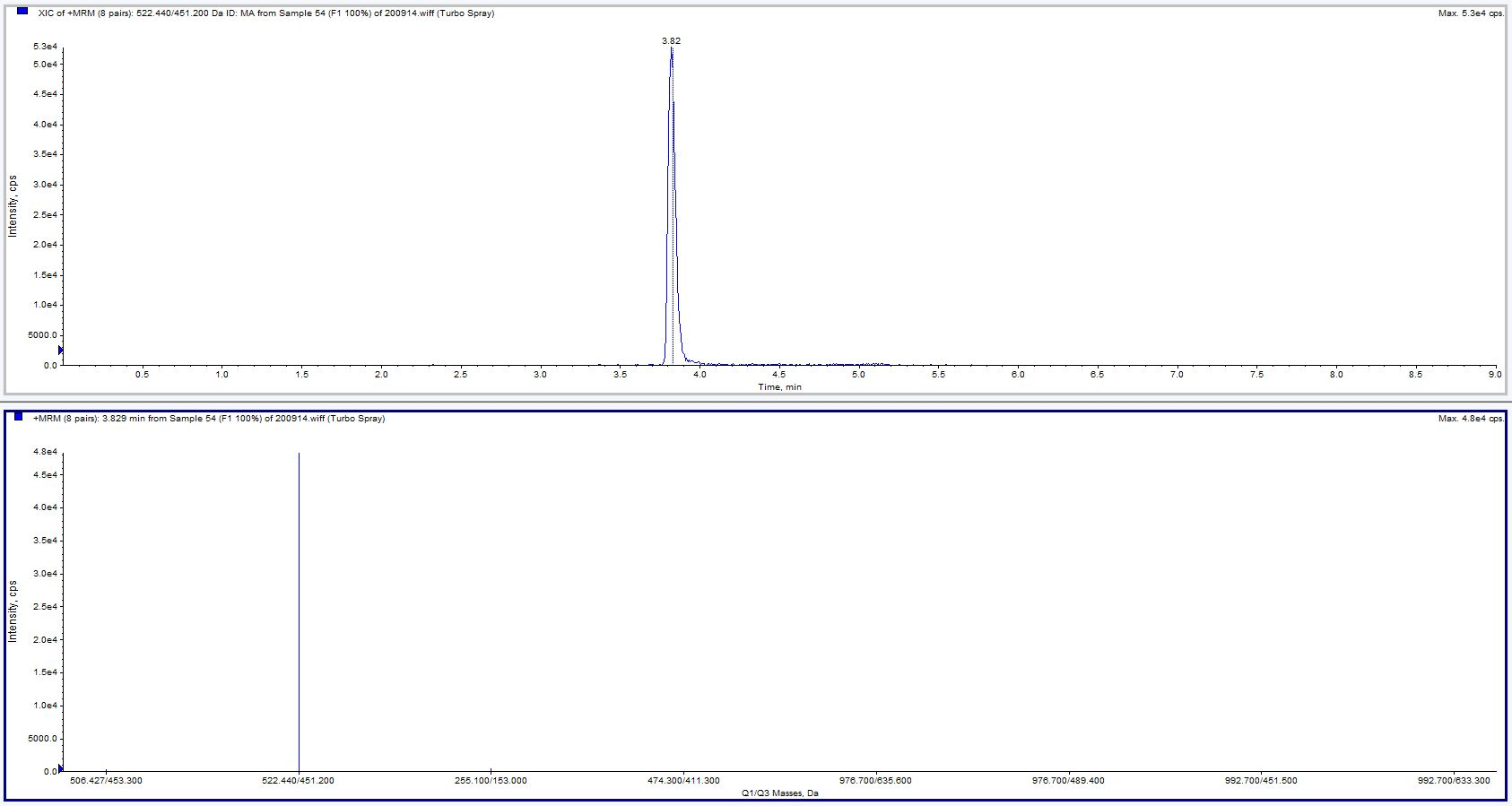

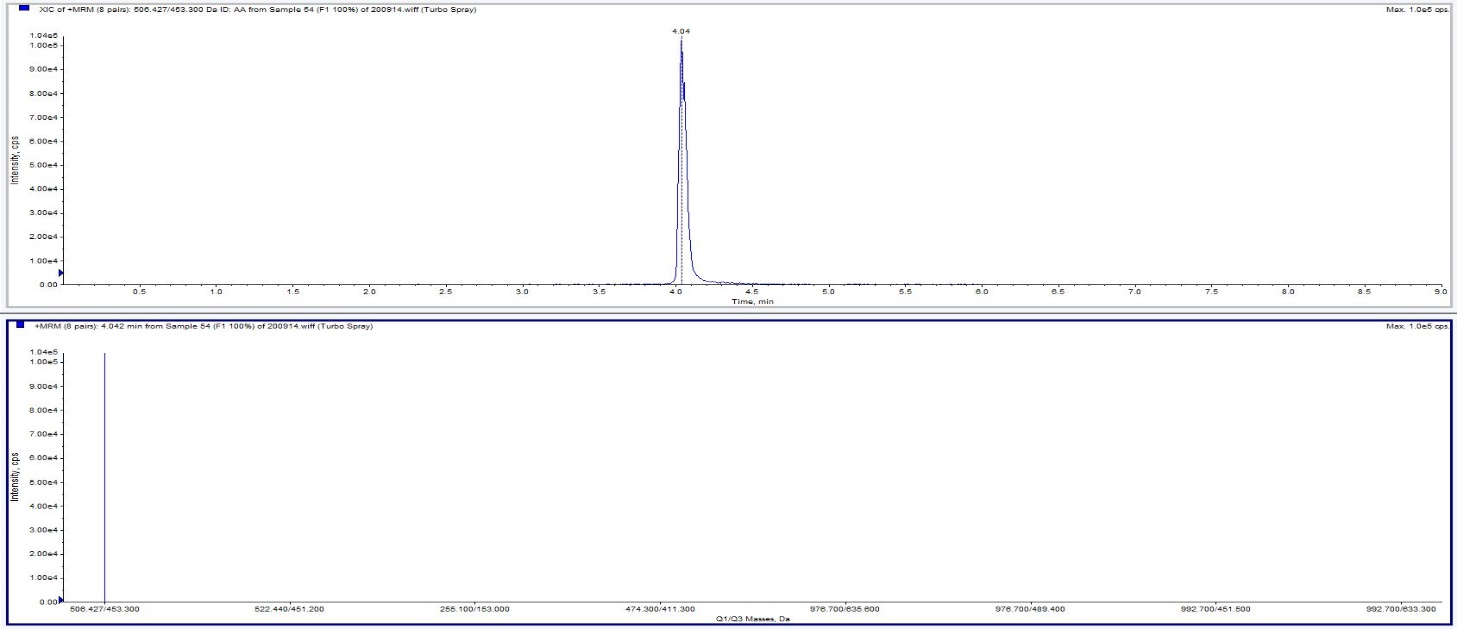


**A**

**A**

**B**

**B**


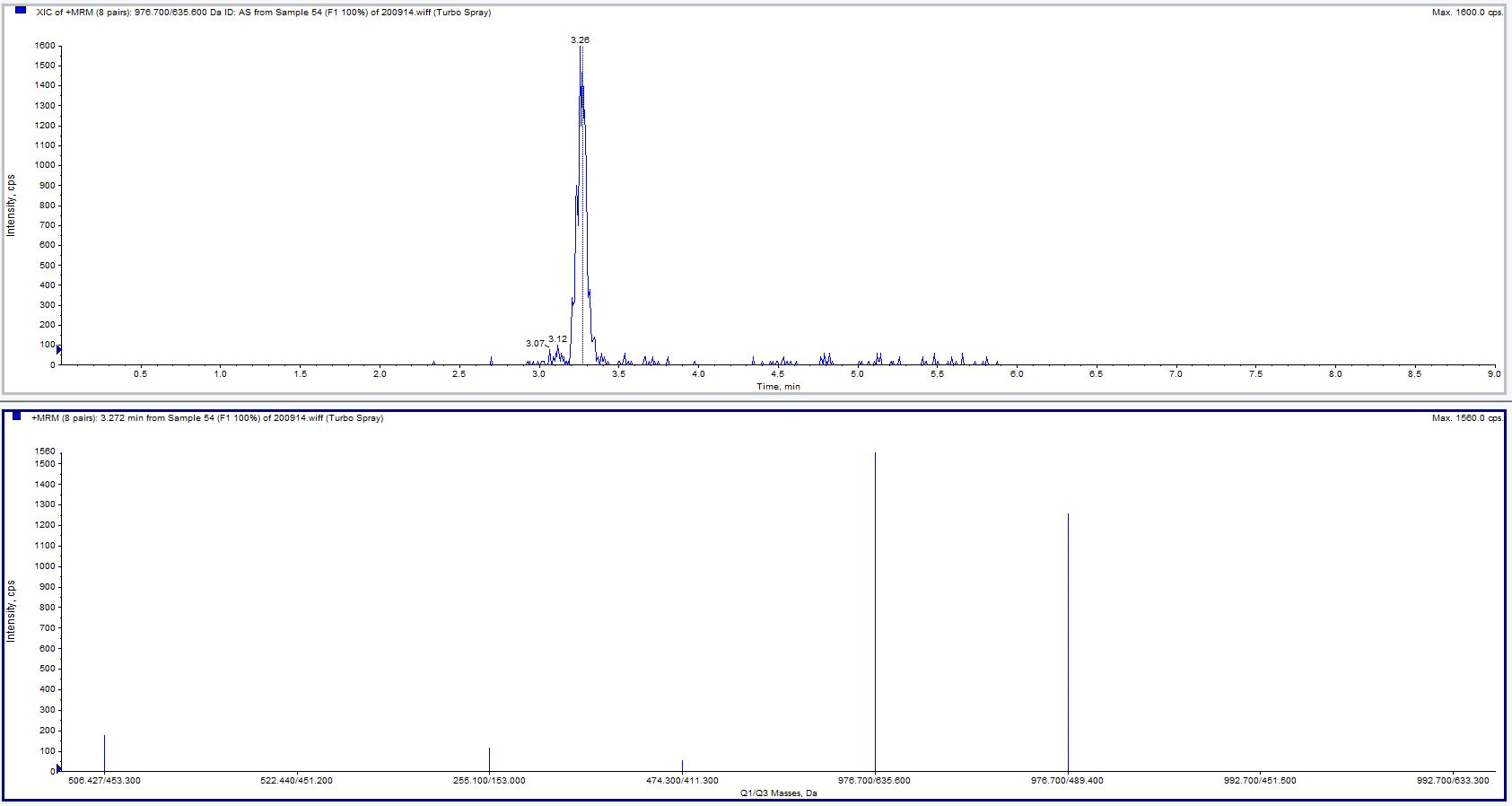


**C**

**C**

**Supplementary Figure S5.** Chromatograms and spectra of triterpenes from *Centella asiatica* in spiked human plasma. The chromatogram and spectra were obtained in positive ion mode electrospray ionization using the following transitions (m/z): Asiatic acid (506/453), madecassic acid (522/451), asiaticoside (976/453; 976/635), and madecassoside (992/487; 992/451). A) Asiatic acid; B) Madecassic acid; C) Asiaticoside; D) Madecassoside.


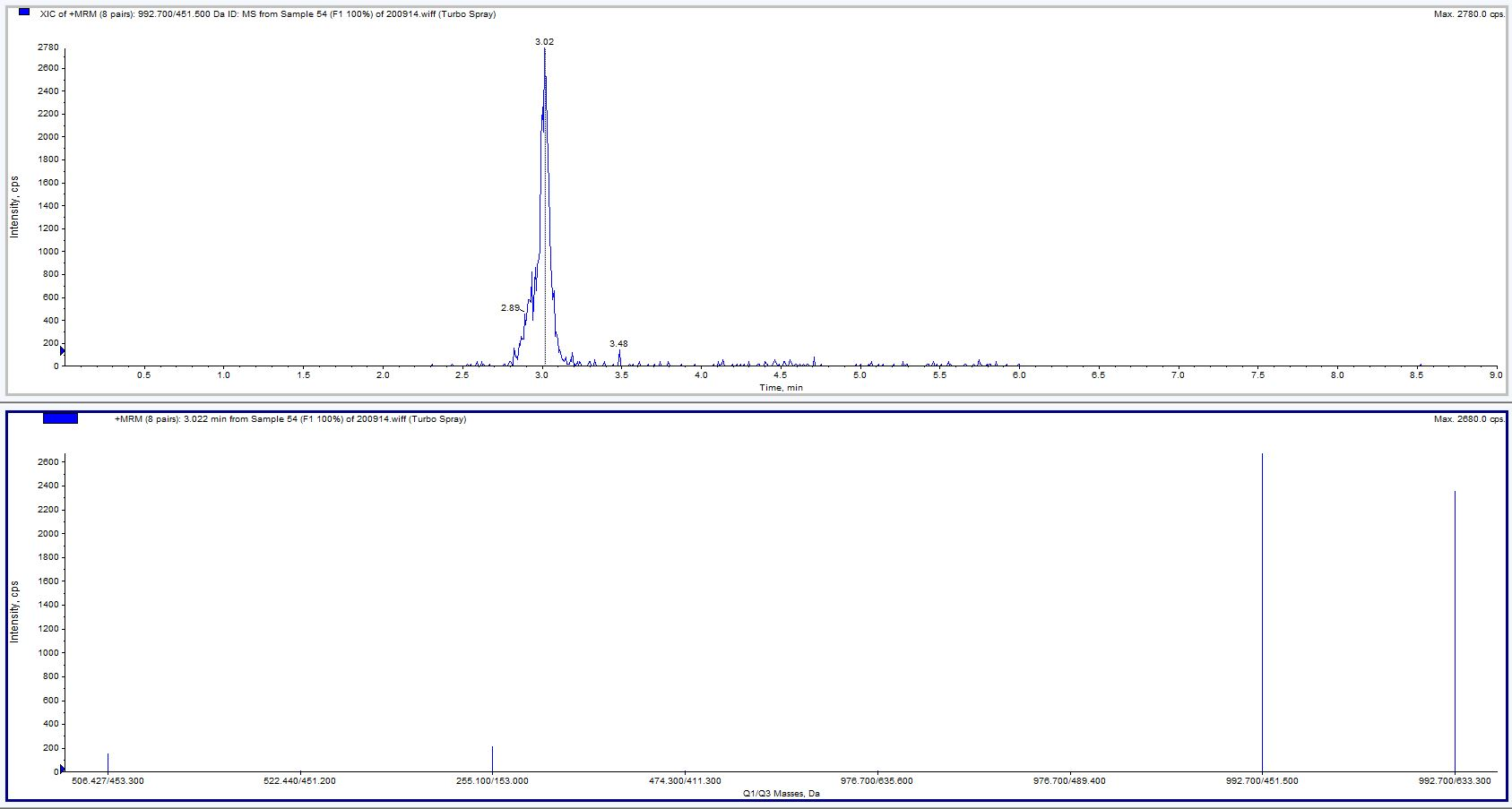


**D**

**D**


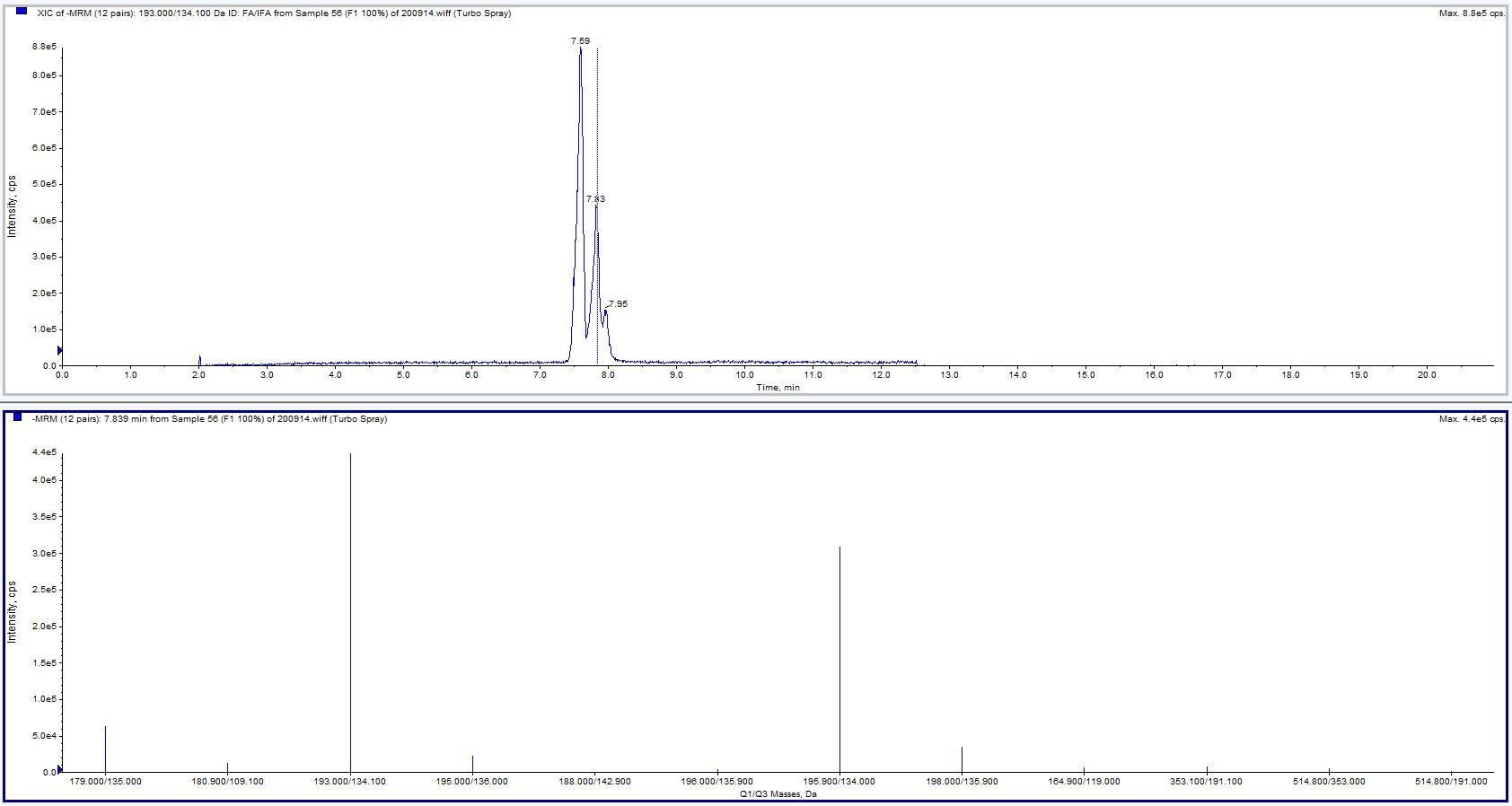

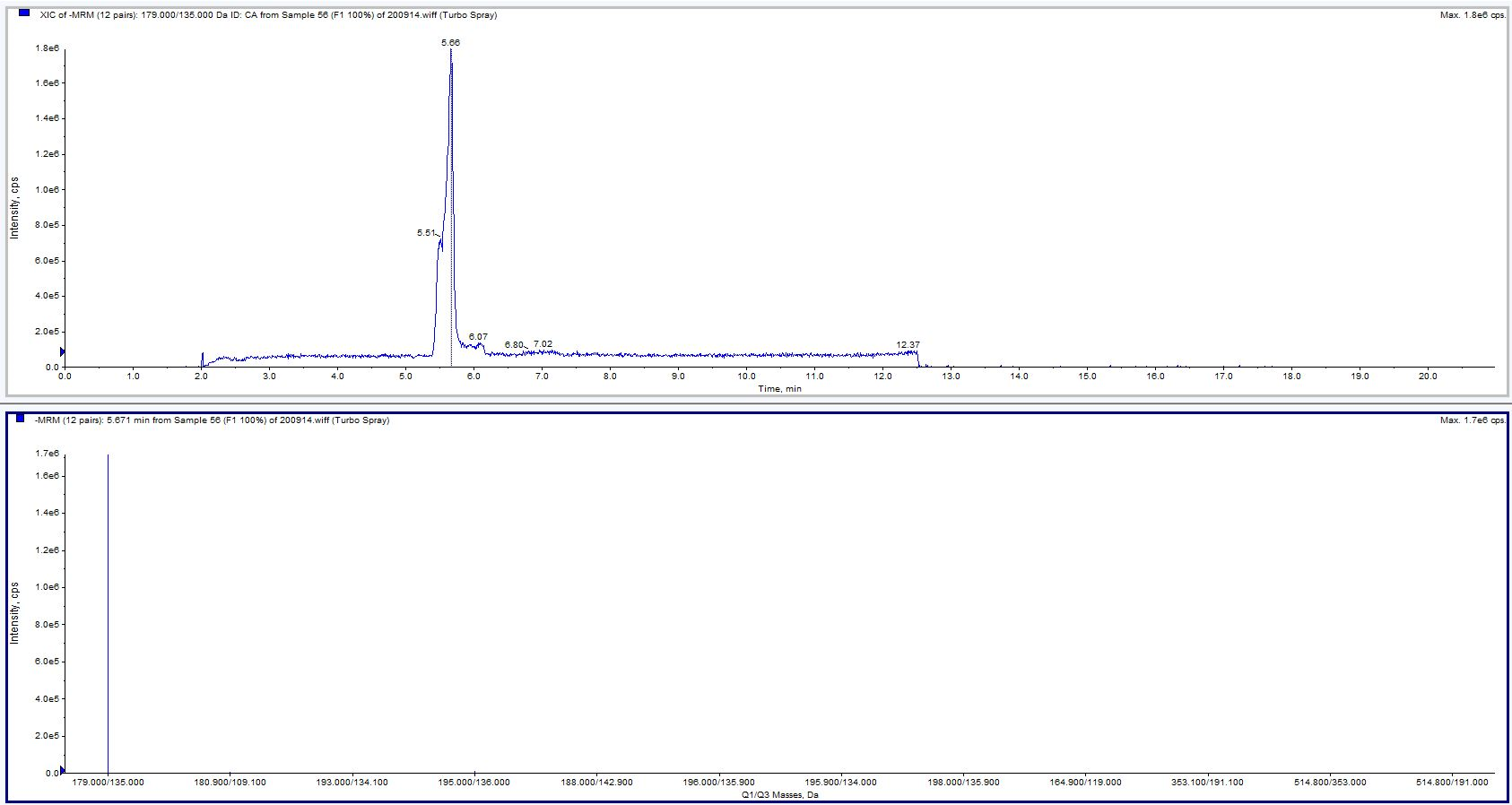

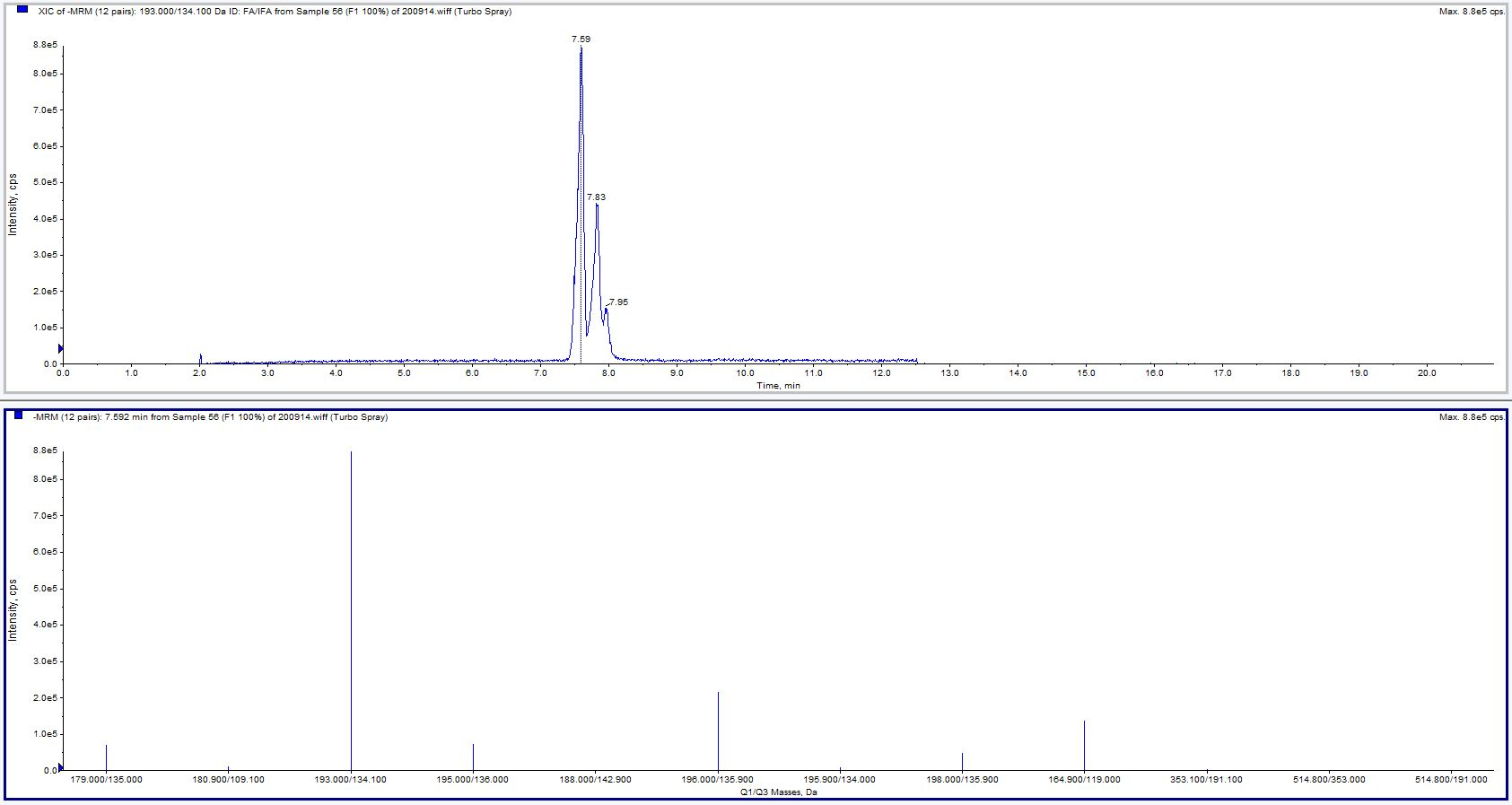


**A**

**A**

**B**

**B**

**C**

**C**


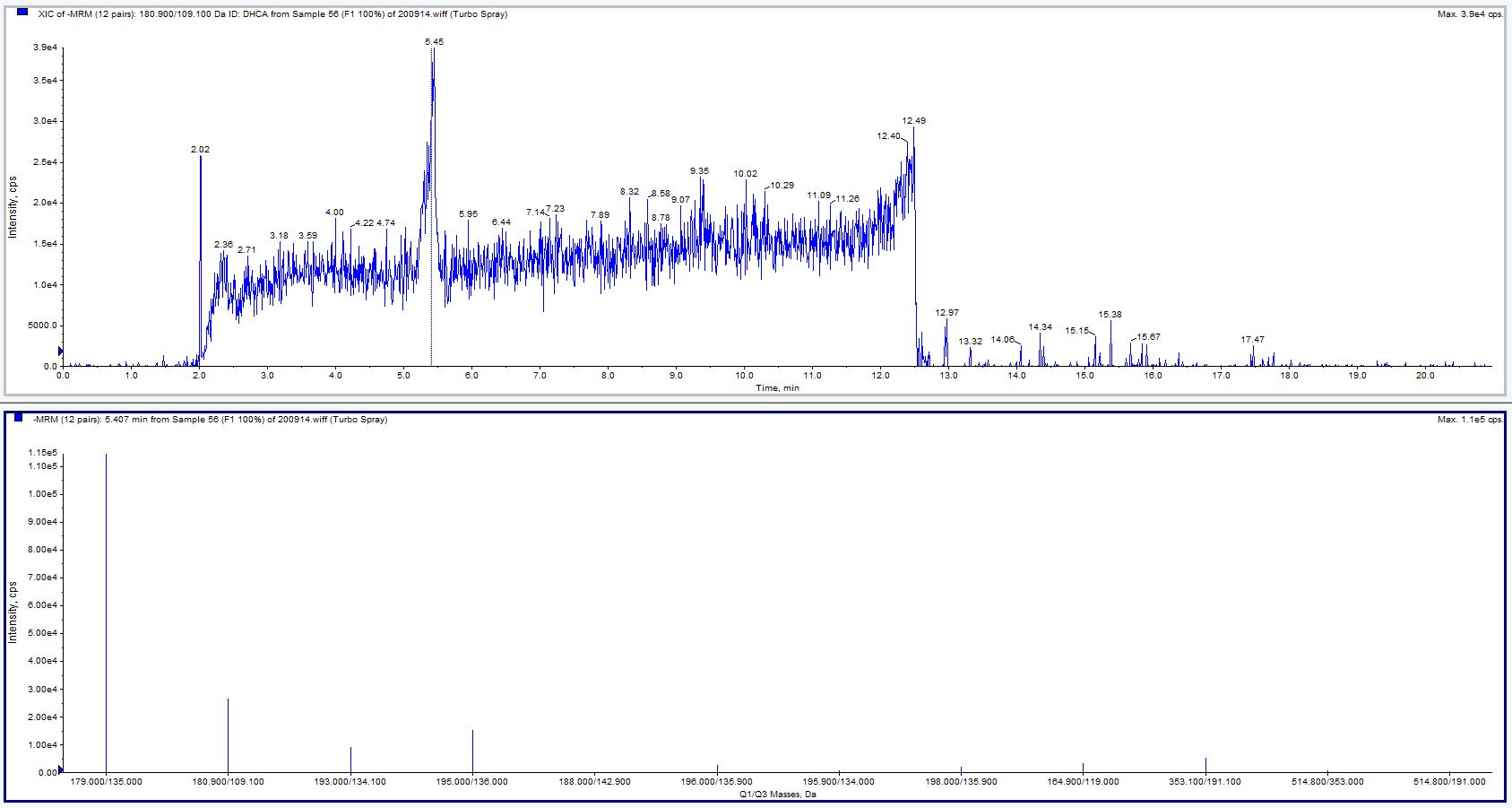


**D**

**D**


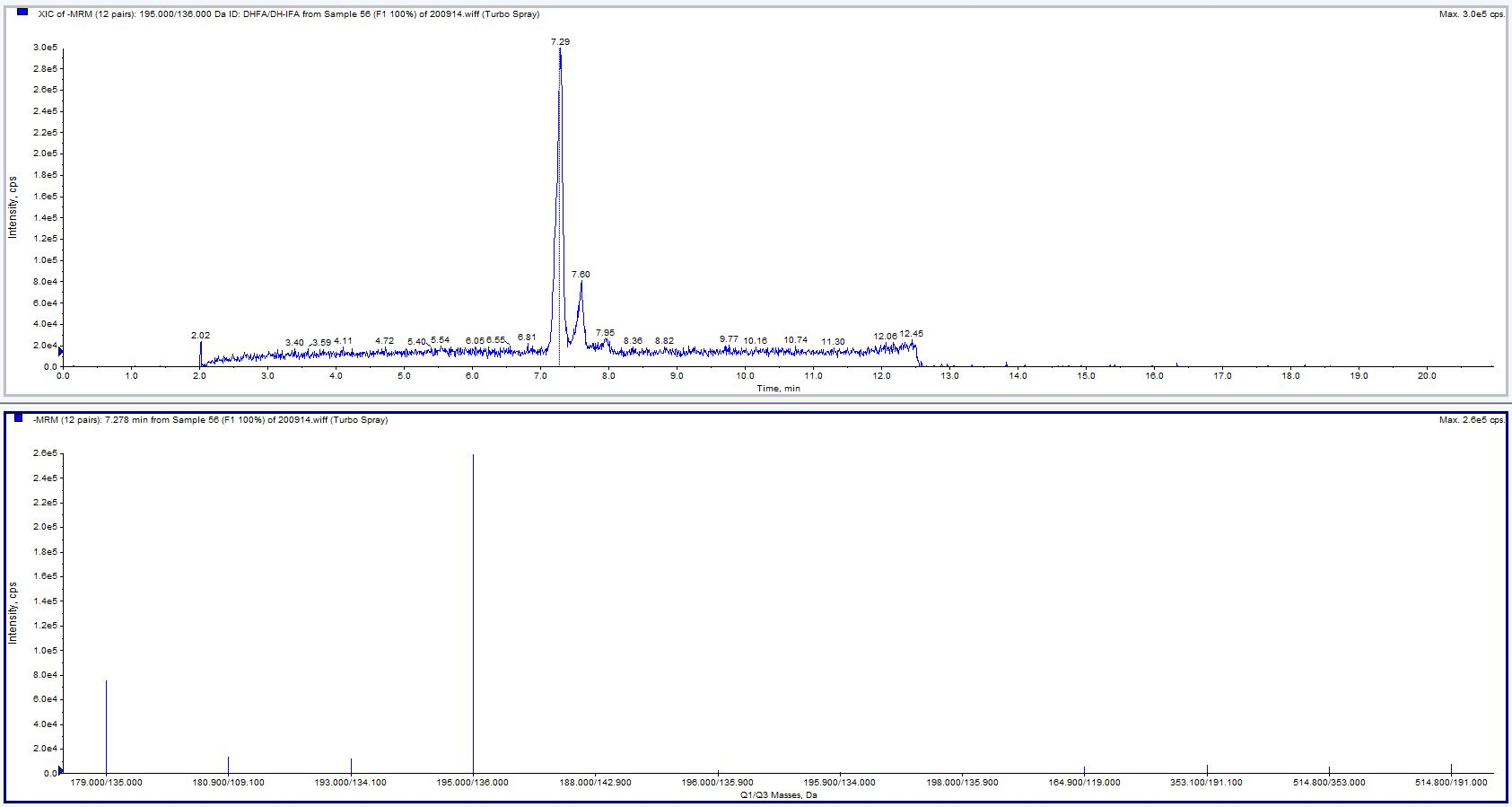

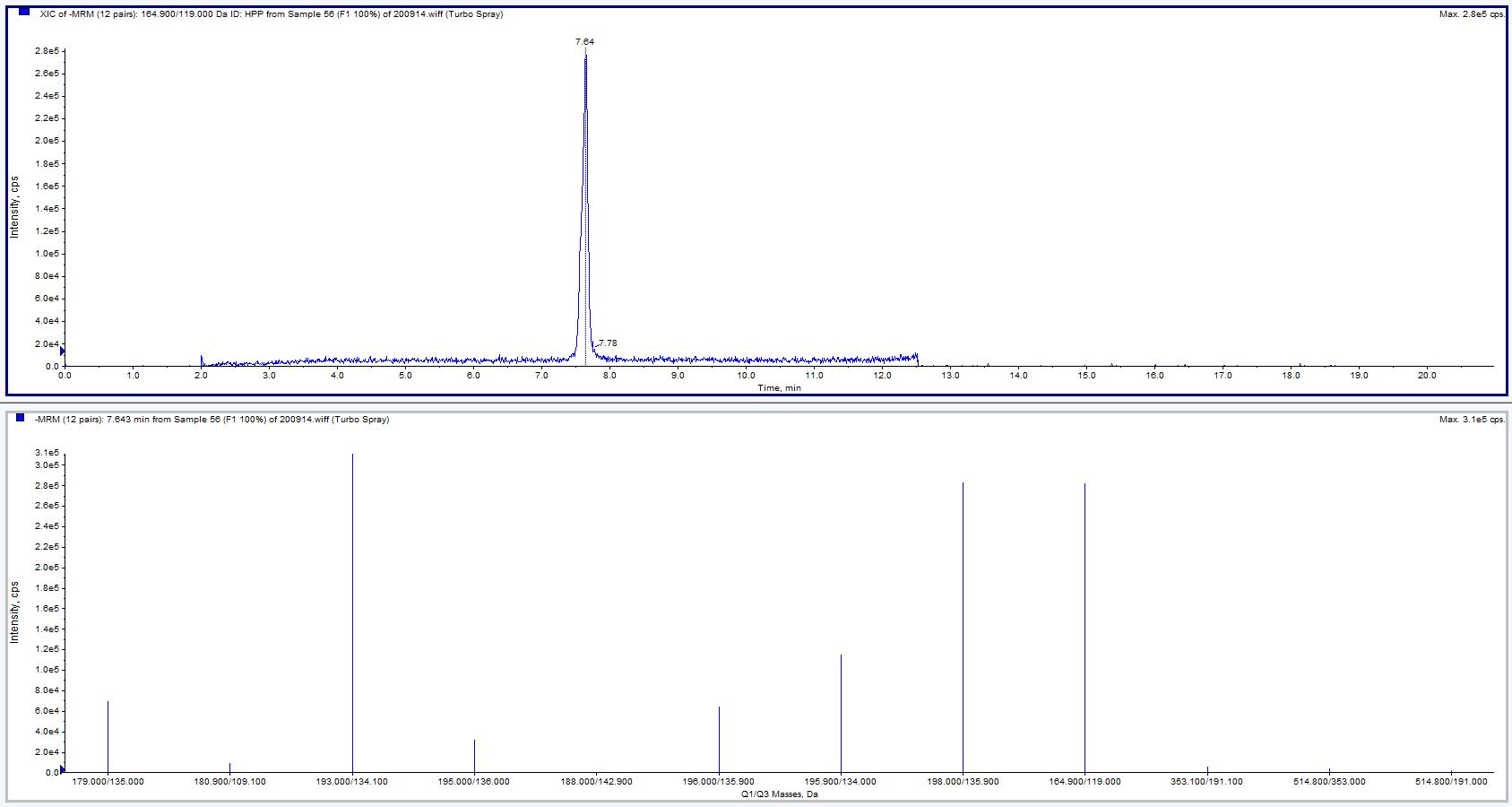


**E**

**E**

**F**

**F**


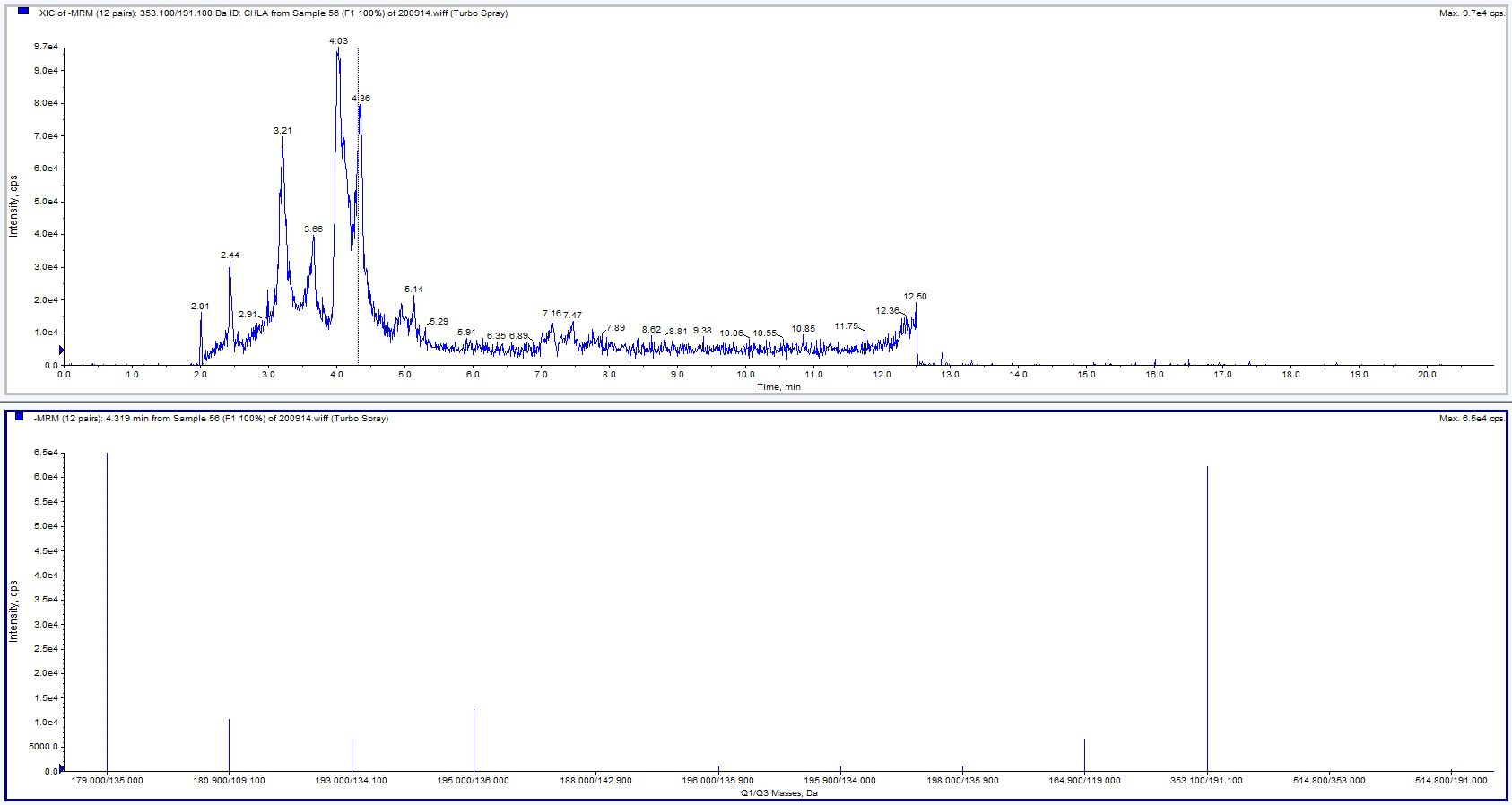


**I**

**I**


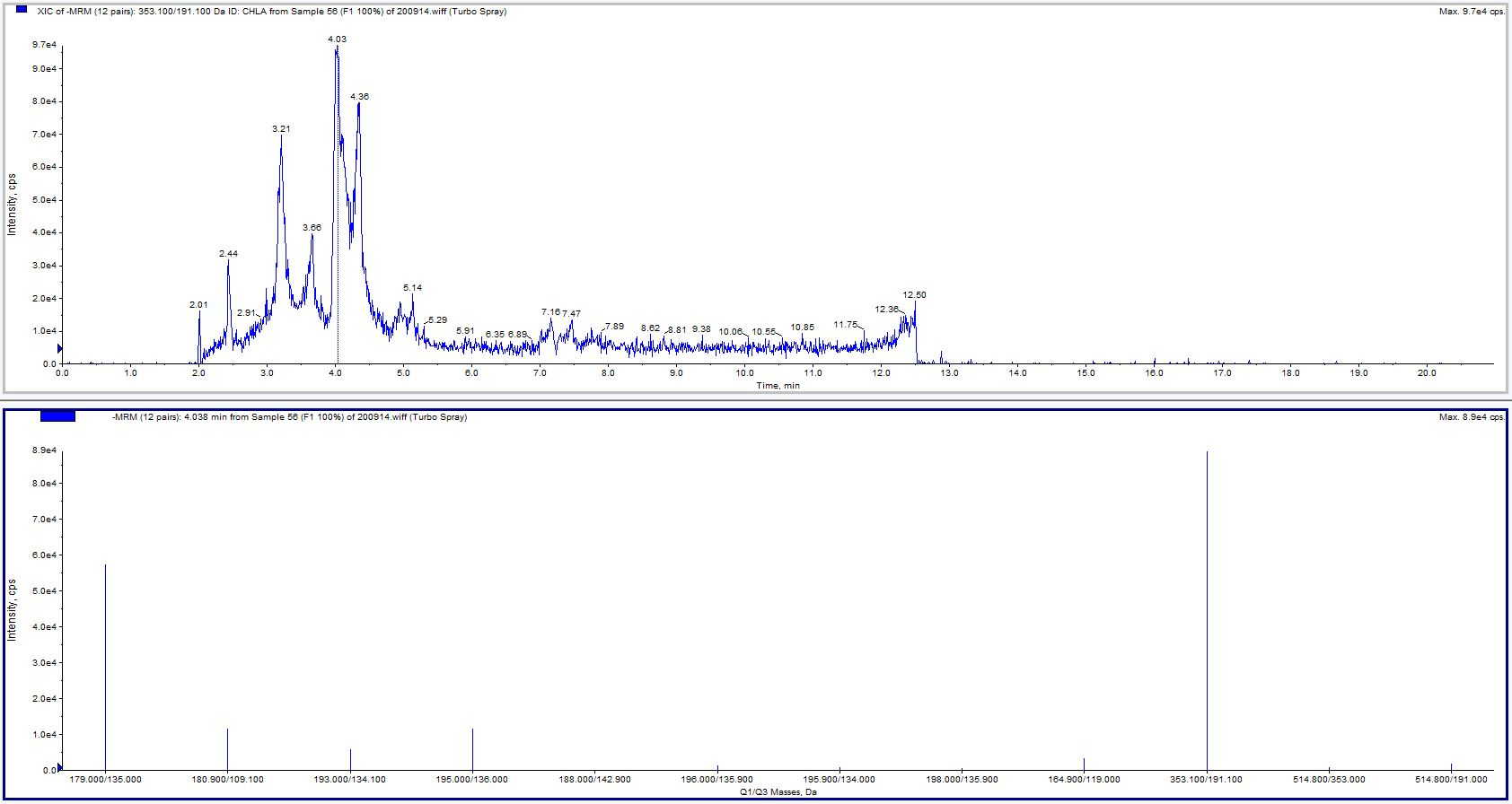

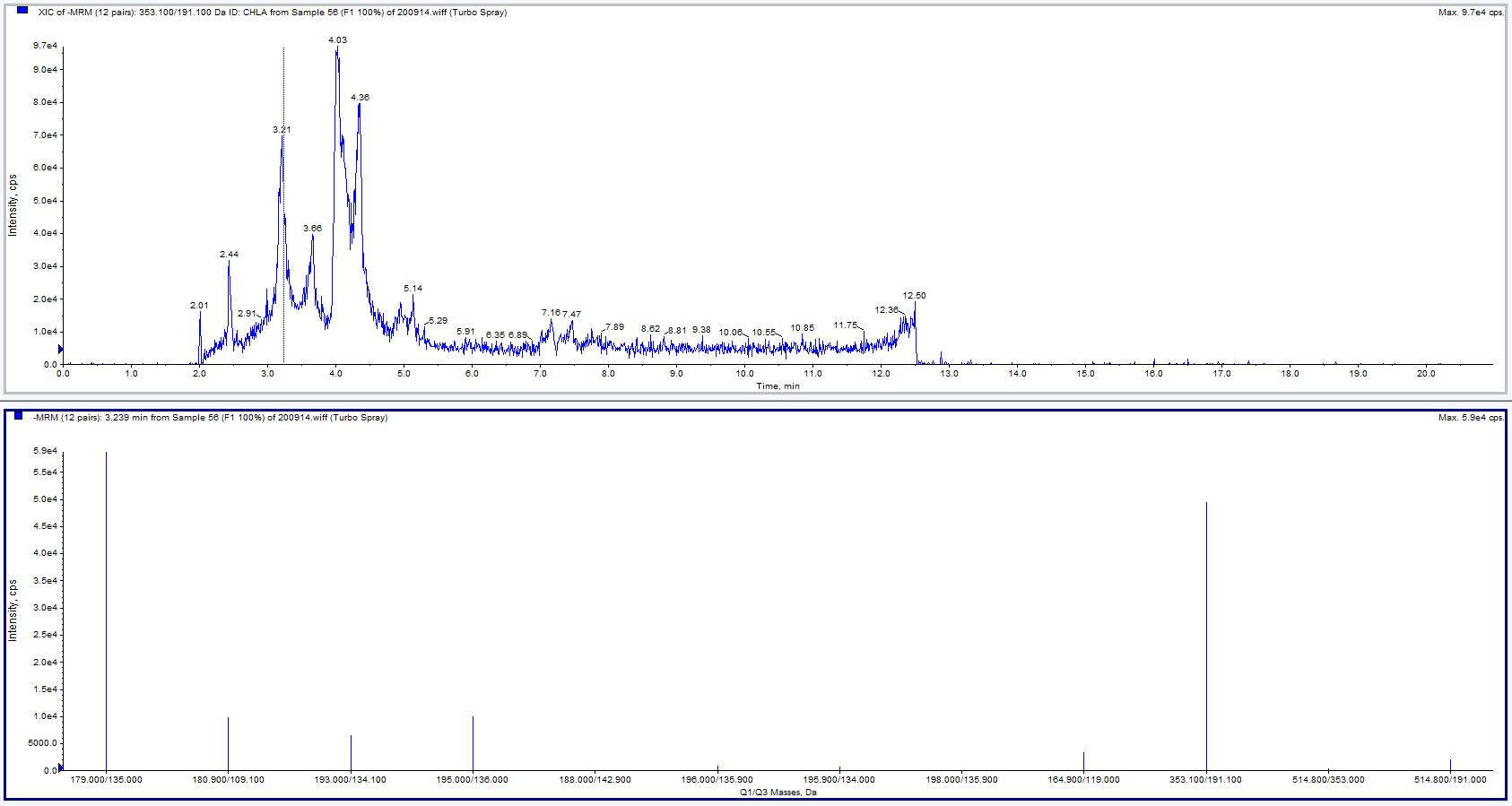


**G**

**G**

**H**

**H**


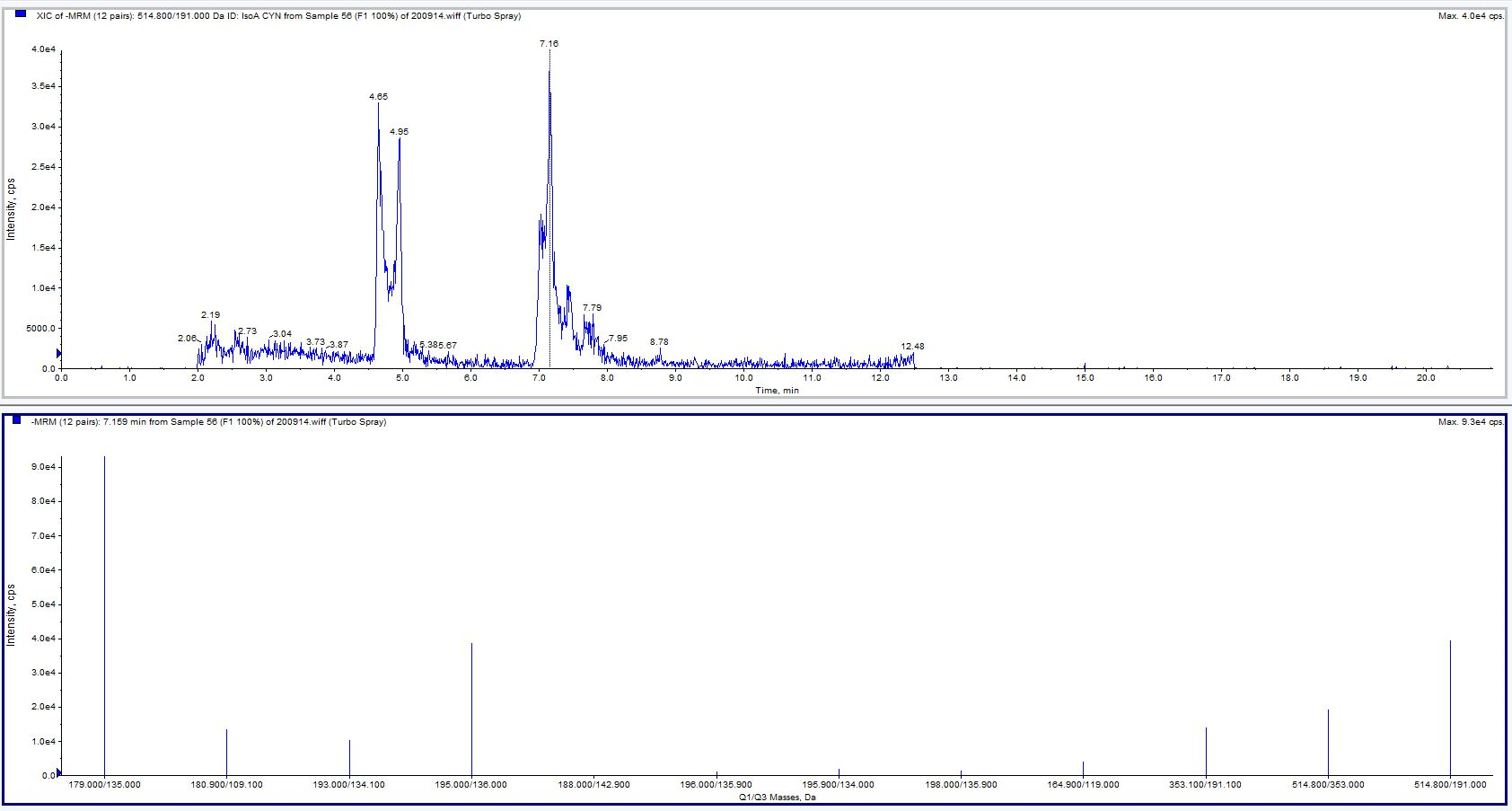

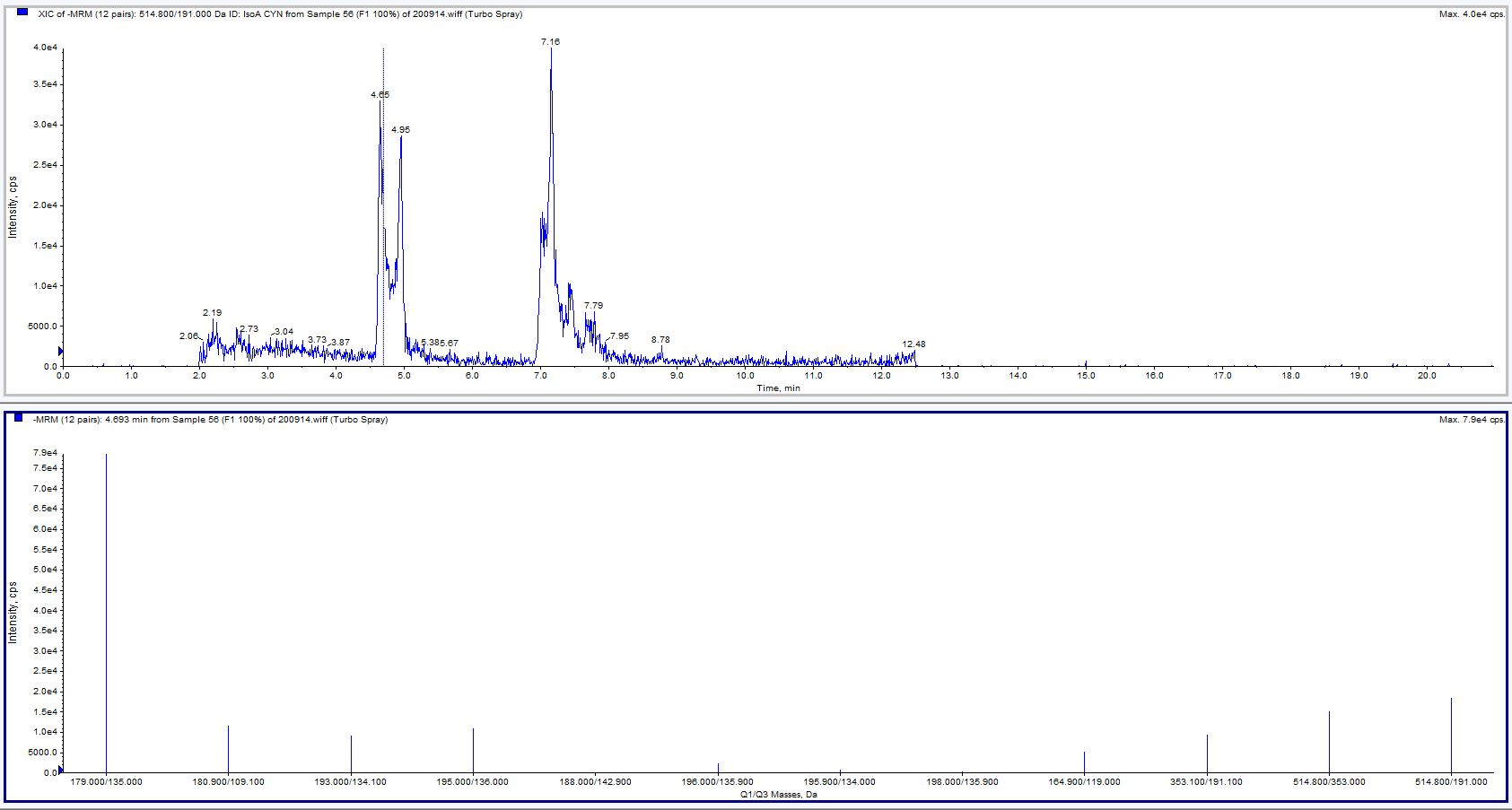

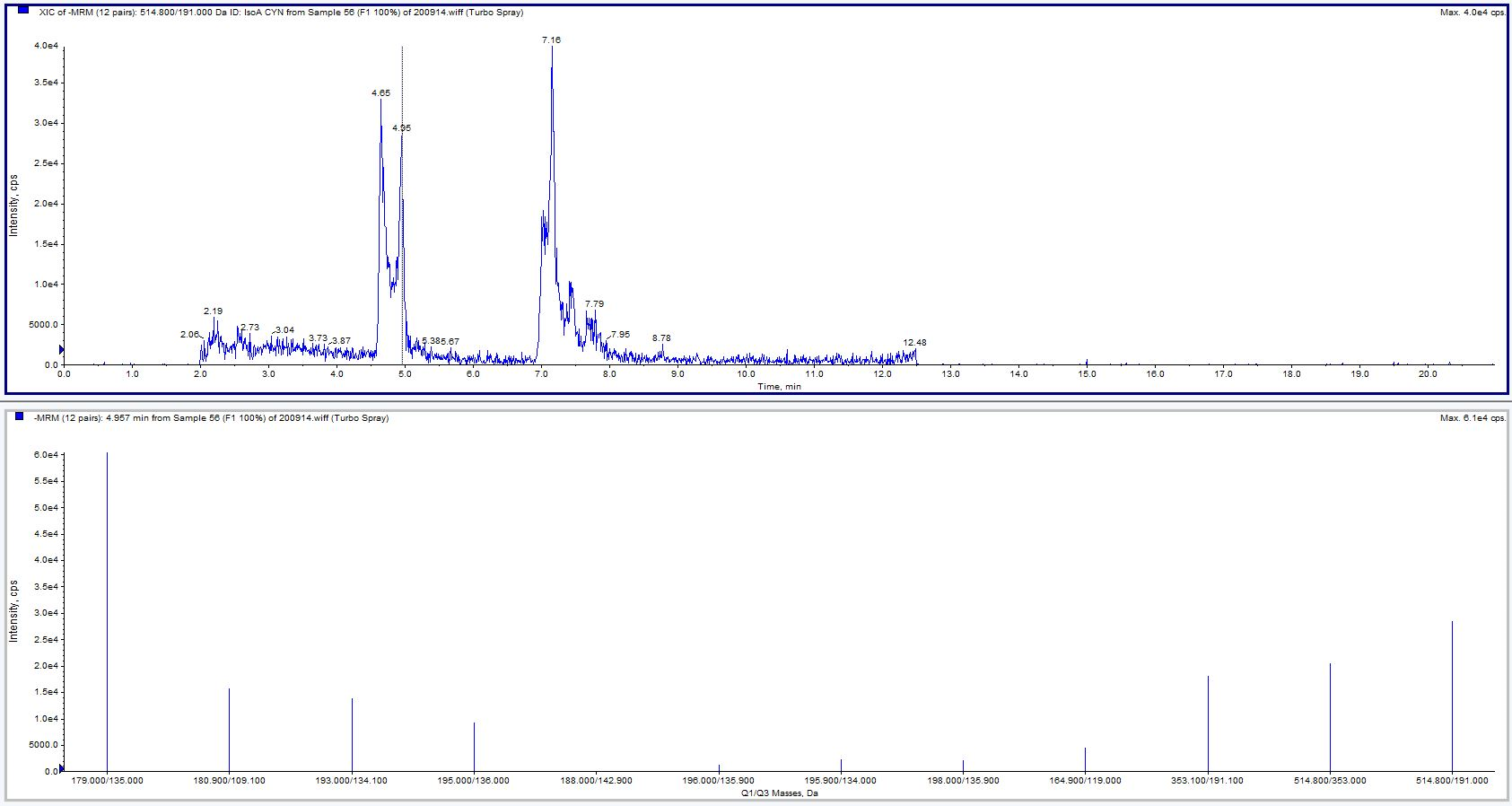


**J**

**J**

**K**

**K**

**L**

**L**


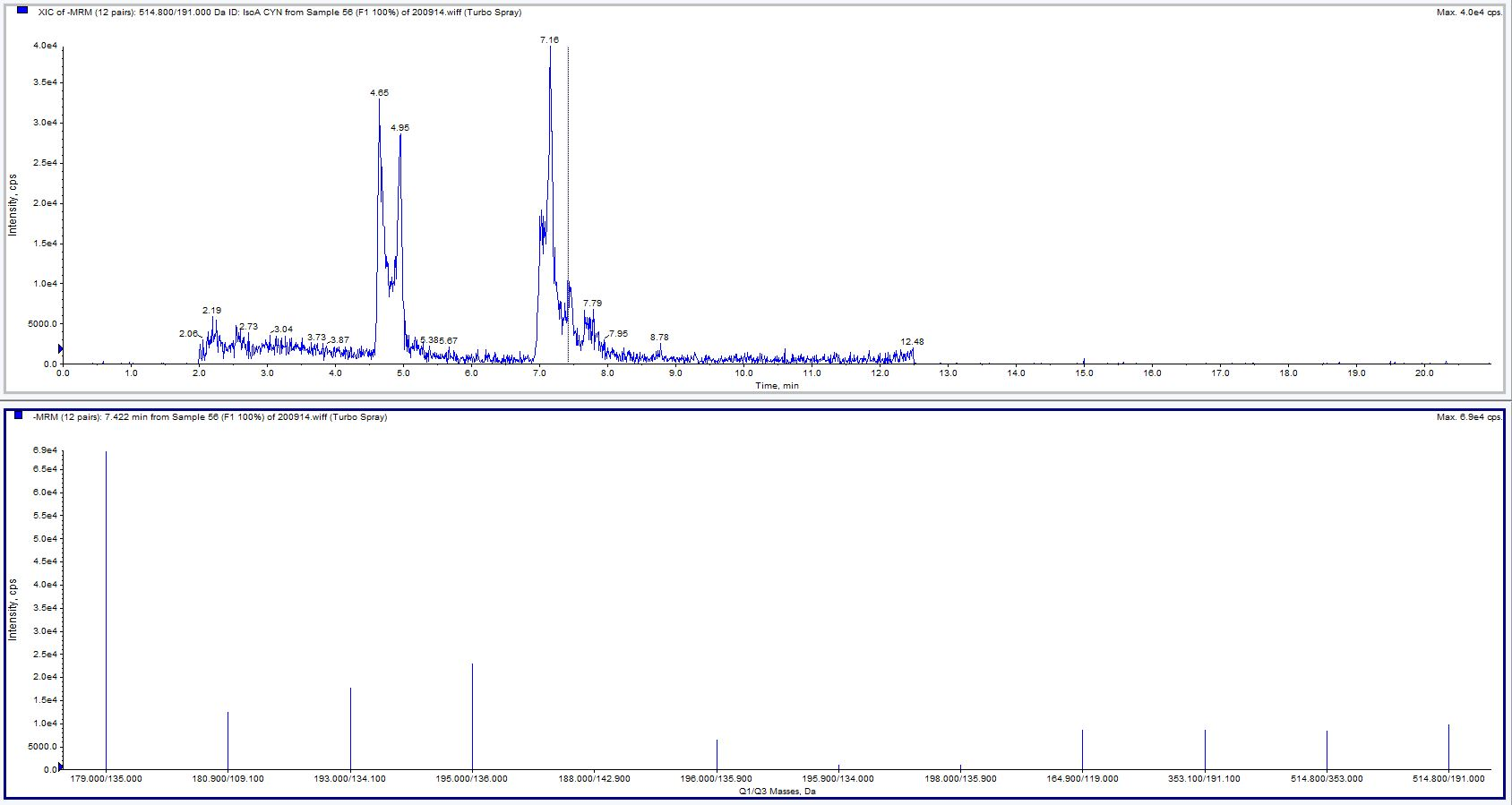

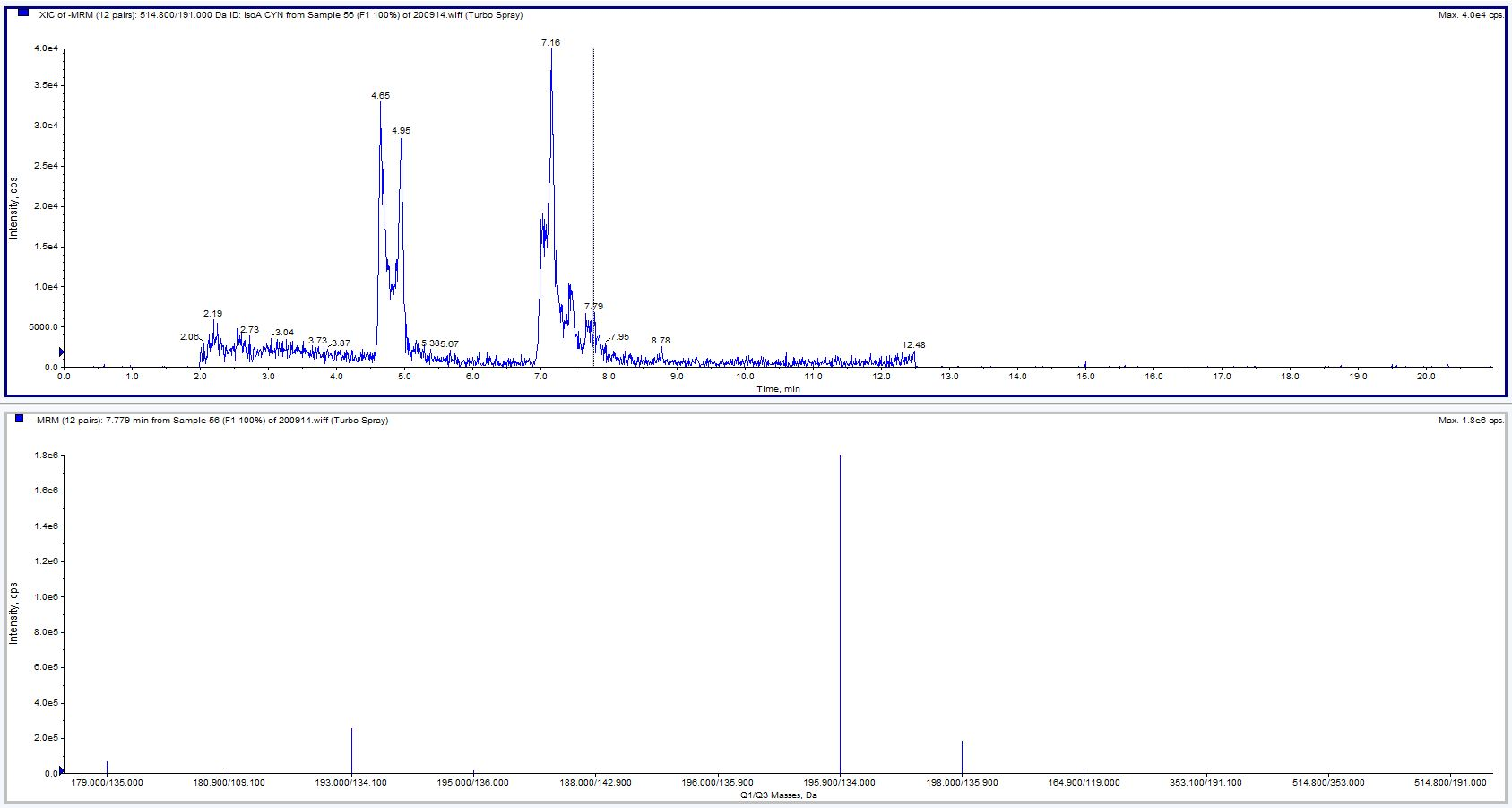


**M**

**M**

**N**

**N**

**Supplementary Figure S6.** Chromatograms and spectra of caffeoylquinic acids and metabolites from *Centella asiatica* in spiked human plasma. The chromatograms and spectra were obtained in negative ion mode using the following MS/MS transitions (m/z): mono-caffeoylquinic acids (353/191); di-caffeoylquinic acids (515/353; 515/191); caffeic acid (179/135); ferulic acid and isoferulic acid (193/134); dihydrocaffeic acid (181/109); dihydroferulic acid (195/136), 3-(3-hydroxyphenyl)propionic acid (165/106). A) Caffeic acid; B) Ferulic acid; C) Isoferulic acid; D) Dihydrocaffeic acid; E) Dihydroferulic acid; F) 3-(3-hydroxyphenyl)propionic acid; G) Neocholorogenic acid; H) Chlorogenic acid; I) Cryptochlorogenic acid; J) 1,3-Di-caffeoylquinic acid/1,4-Di-caffeoylquinic acid; K) 1,5-Dicaffeoylquinic acid; L) Isochlorogenic acid A; M) Isochlorogenic acid B; N) Isochlorogenic acid C

**
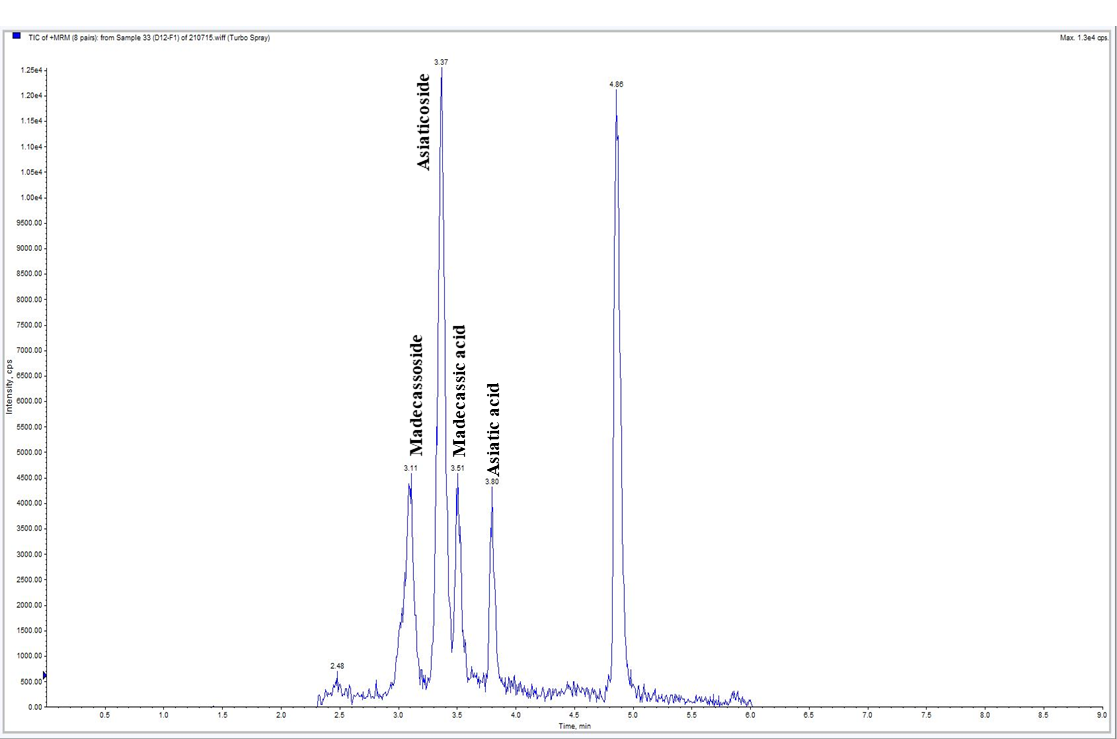
**

1.25e4

**Supplementary Figure S7.** Total ion chromatogram (TIC) of triterpenes from *Centella asiatica* in spiked human urine. The TIC was obtained in positive ion mode using the following transitions (m/z): Asiatic acid (506/453), Madecassic acid (522/451), Asiaticoside (976/453; 976/635), and Madecassoside (992/487; 992/451).

**
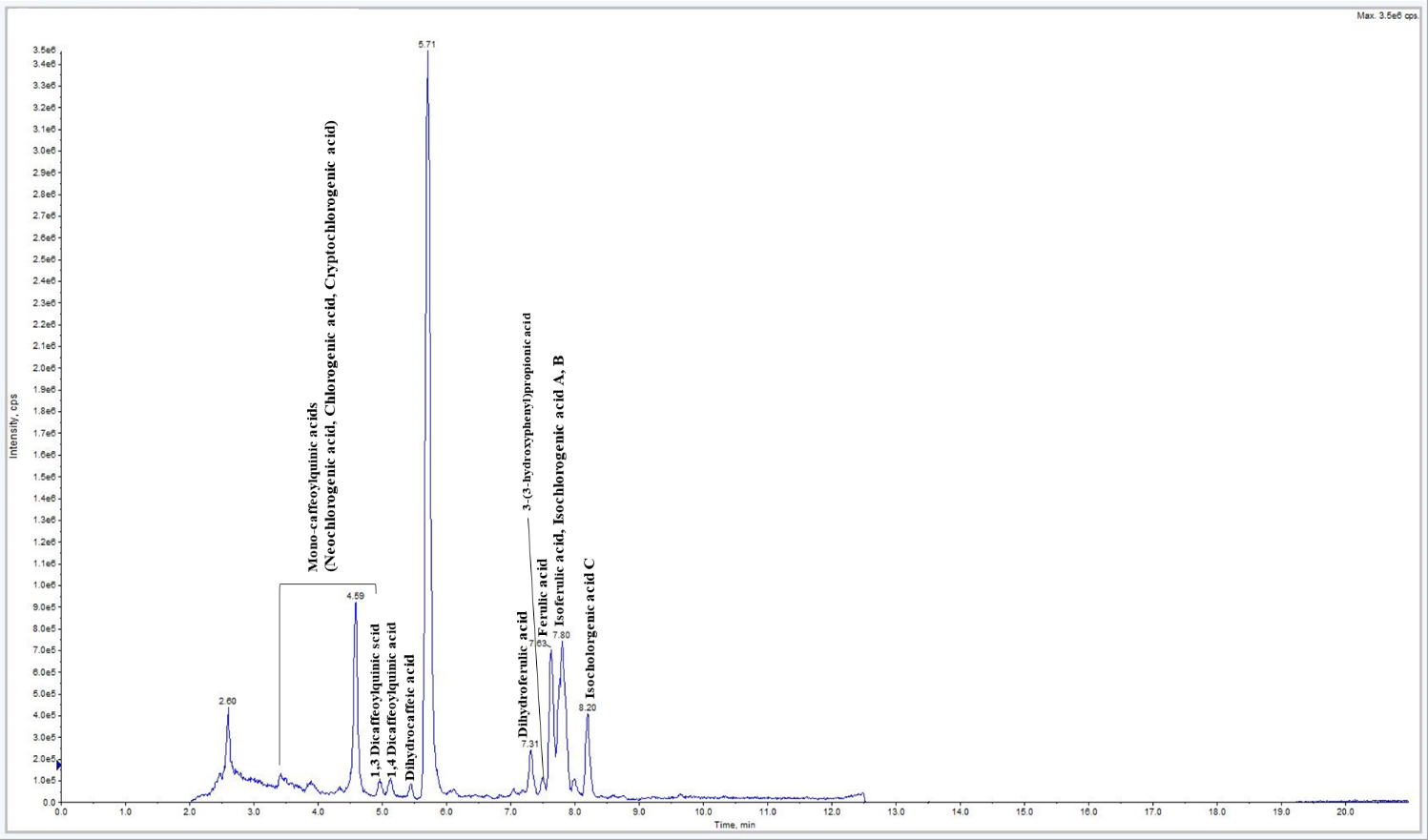
**

3.5e6

**Caffeic acid**

**Supplementary Figure S8.** Total ion chromatogram (TIC) of caffeoylquinic acids from *Centella asiatica* and metabolites in spiked human urine. The TIC was obtained in negative ion mode using the following MS/MS transitions (m/z): mono-caffeoylquinic acids (353/191); di-caffeoylquinic acids (515/353; 515/191); caffeic acid (179/135); ferulic acid and isoferulic acid (193/134); dihydrocaffeic acid (181/109); dihydroferulic acid (195/136), 3-(3-hydroxyphenyl)propionic acid (165/106).


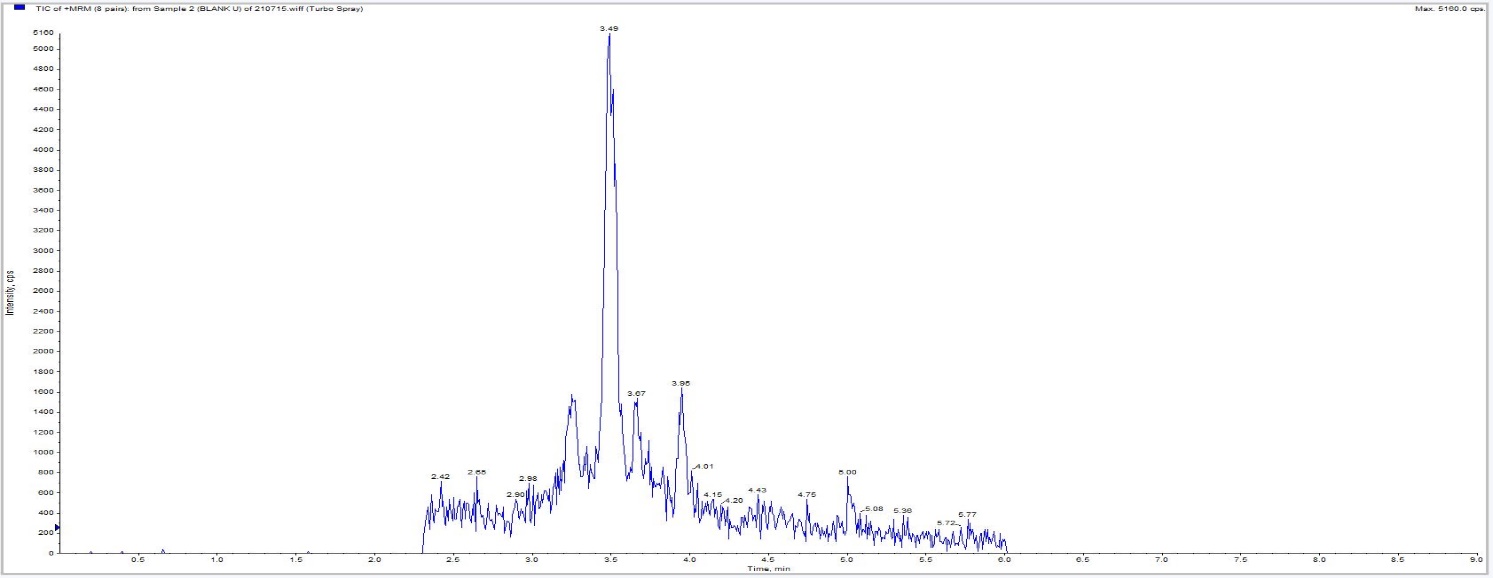


5100

**Supplementary Figure S9.** Total ion chromatogram (TIC) of blank human urine evaluated for triterpenes. The TIC was obtained in positive ion mode.


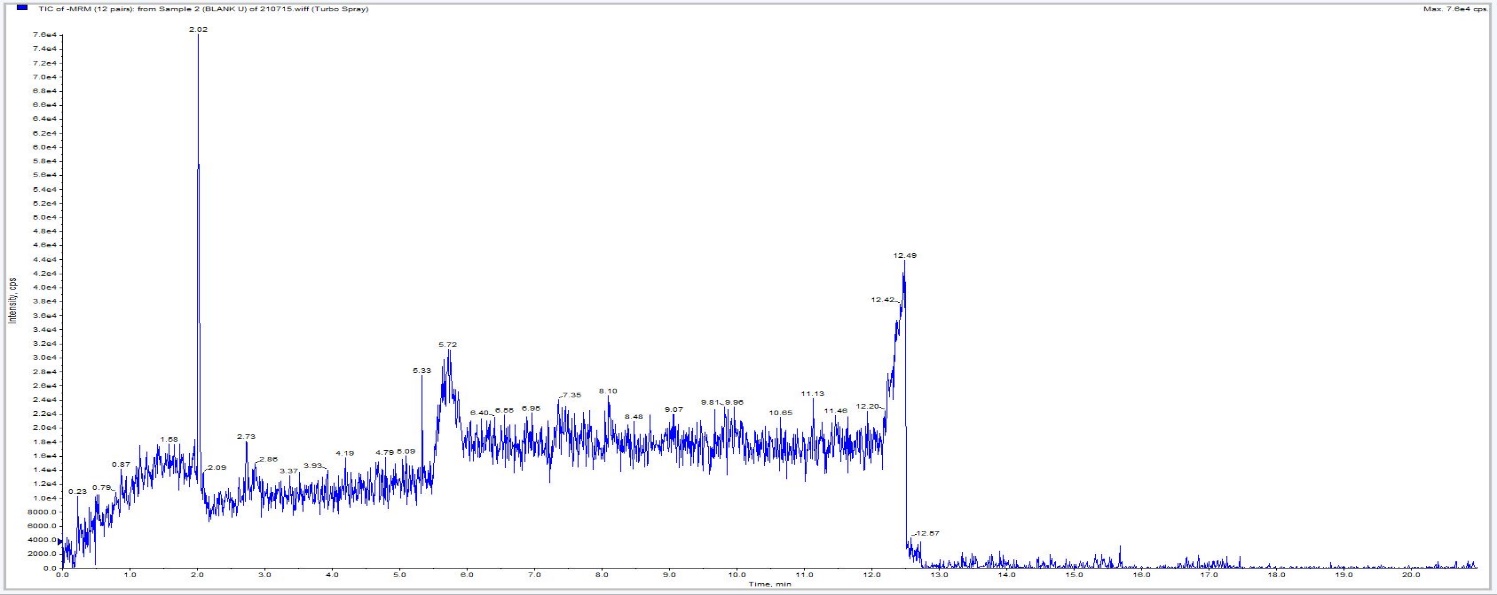


7.6e4

**Supplementary Figure S10.** Total ion chromatogram (TIC) of blank human urine evaluated for caffeoylquinic acids. The TIC was obtained in negative ion mode.

**A**


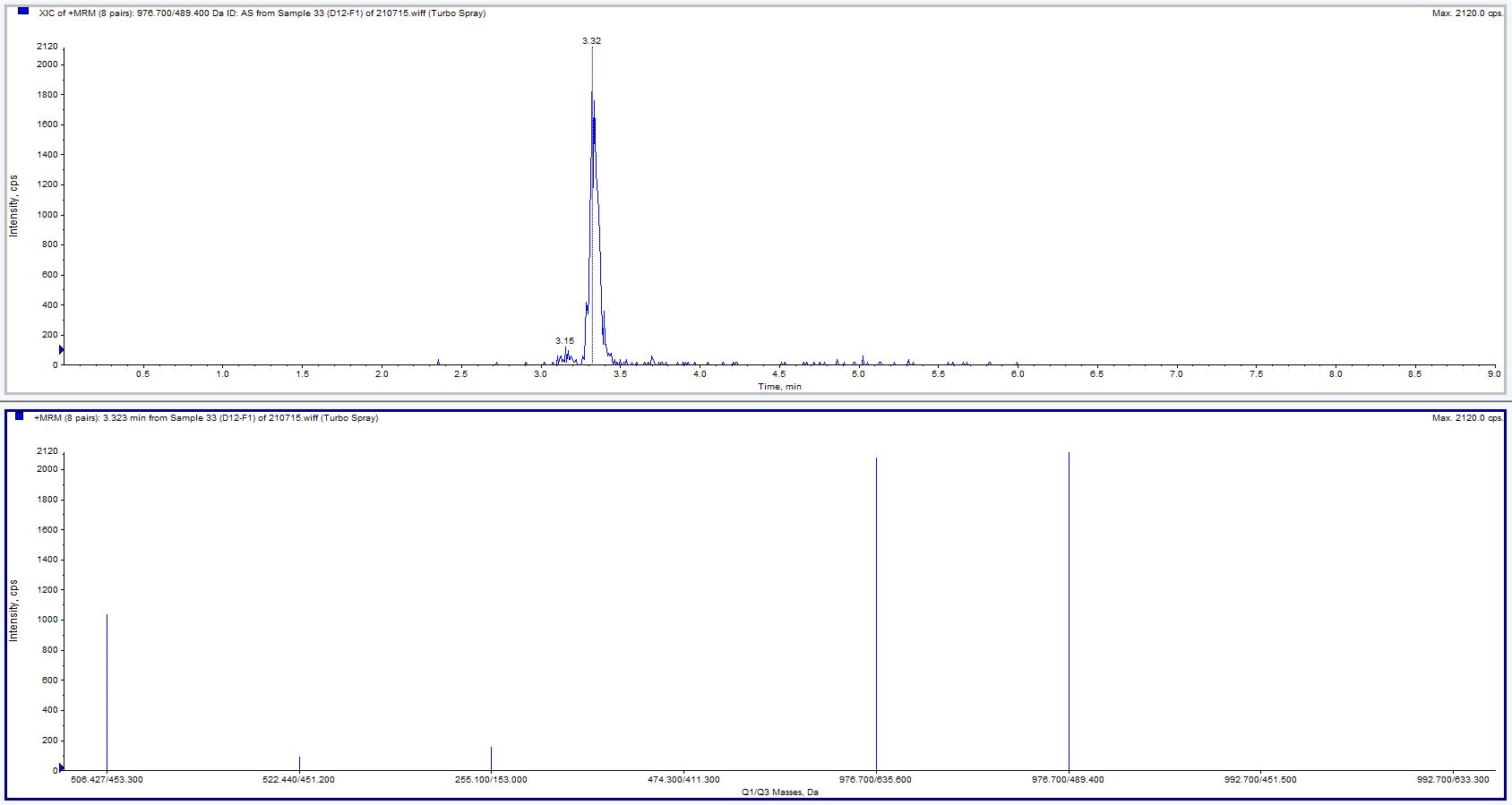

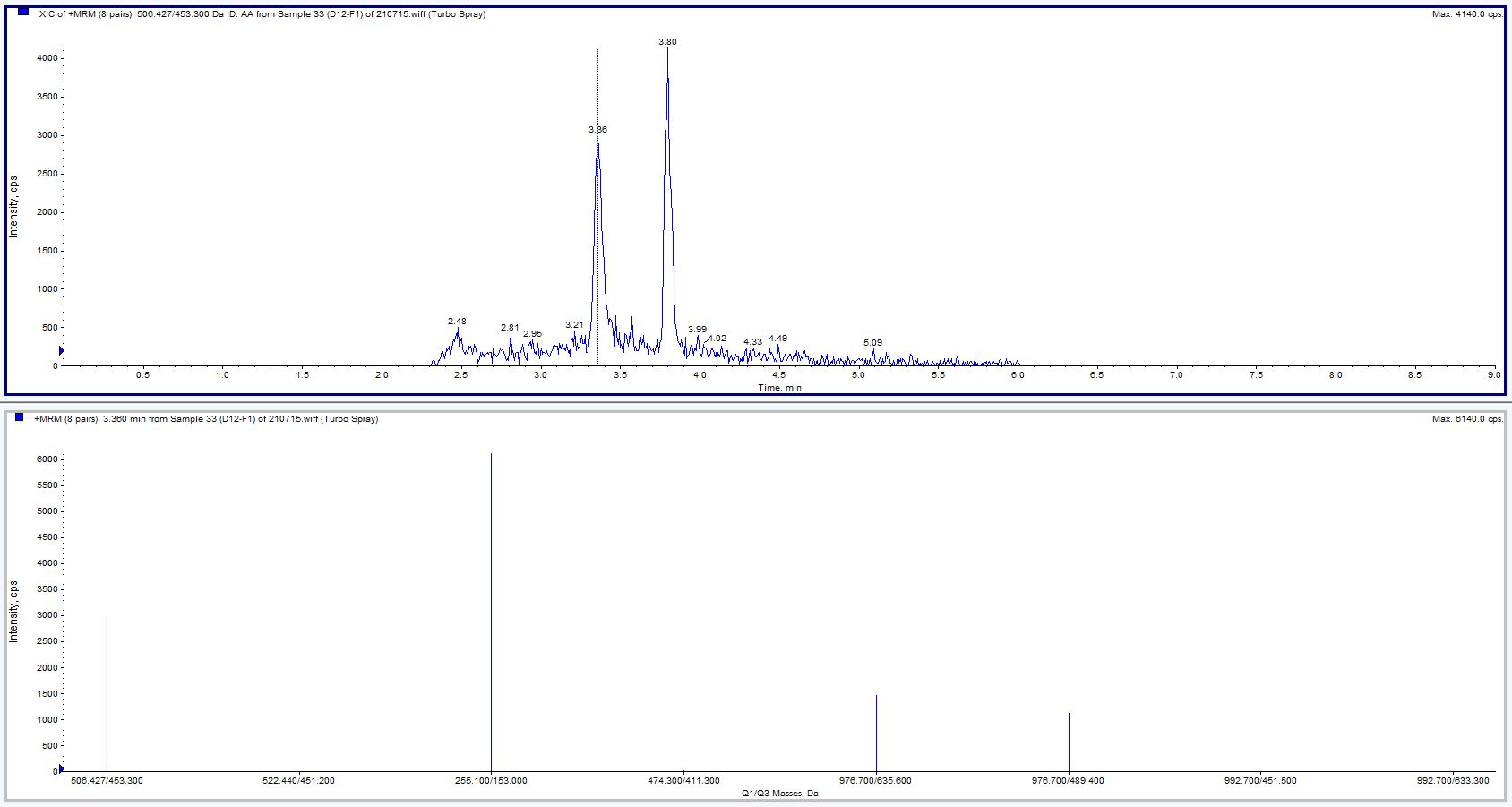

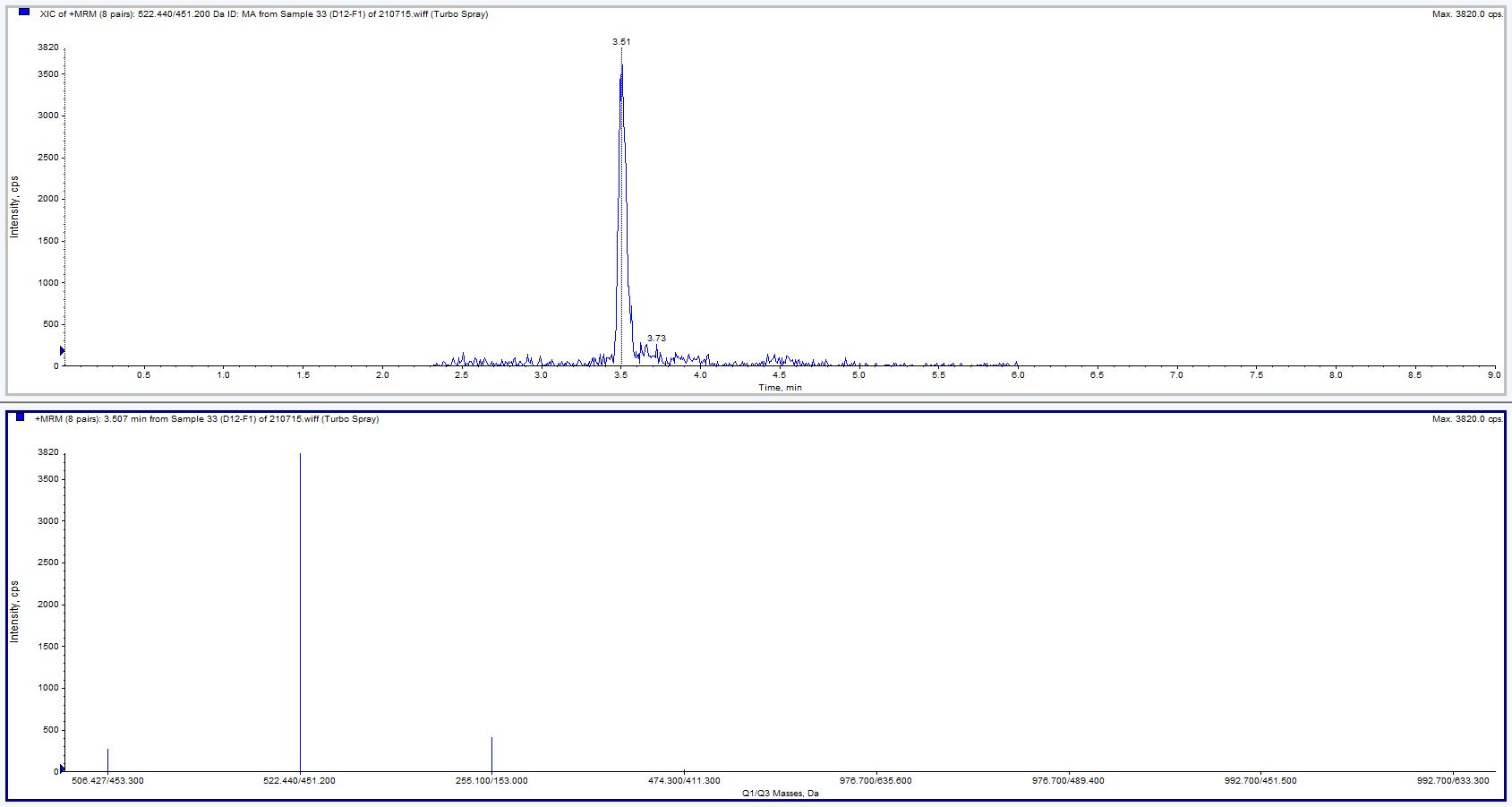


**A**

**B**

**B**

**C**

**C**

**Supplementary Figure S11.** Chromatograms and spectra of triterpenes from *Centella asiatica* in spiked human urine. The chromatogram and spectra were obtained in positive ion mode electrospray ionization using the following transitions (m/z): Asiatic acid (506/453), Madecassic acid (522/451), Asiaticoside (976/453; 976/635), and Madecassoside (992/487; 992/451). A) Asiatic acid; B) Madecassic acid; C) Asiaticoside; D) Madecassoside.


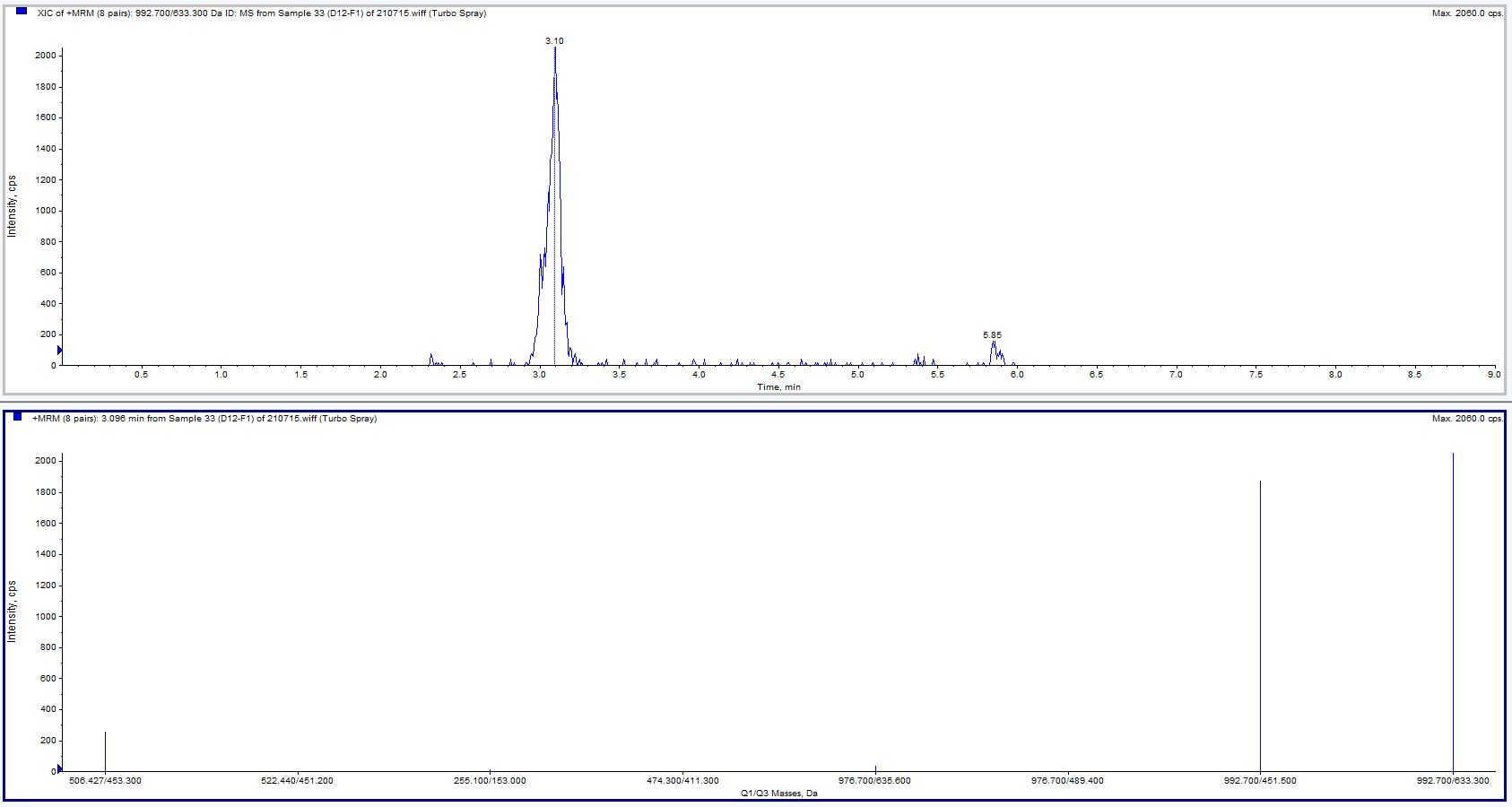


**D**

**D**


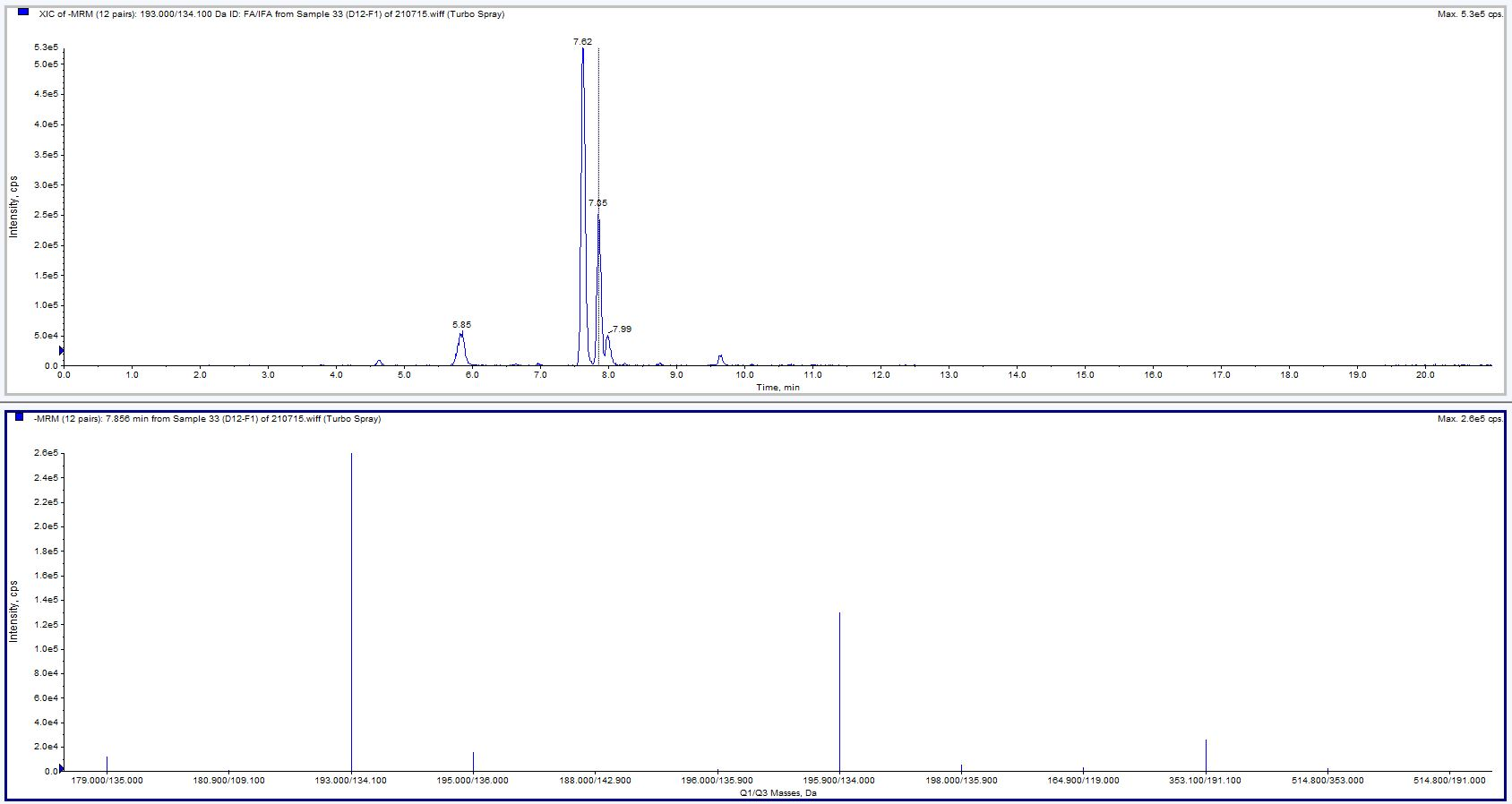

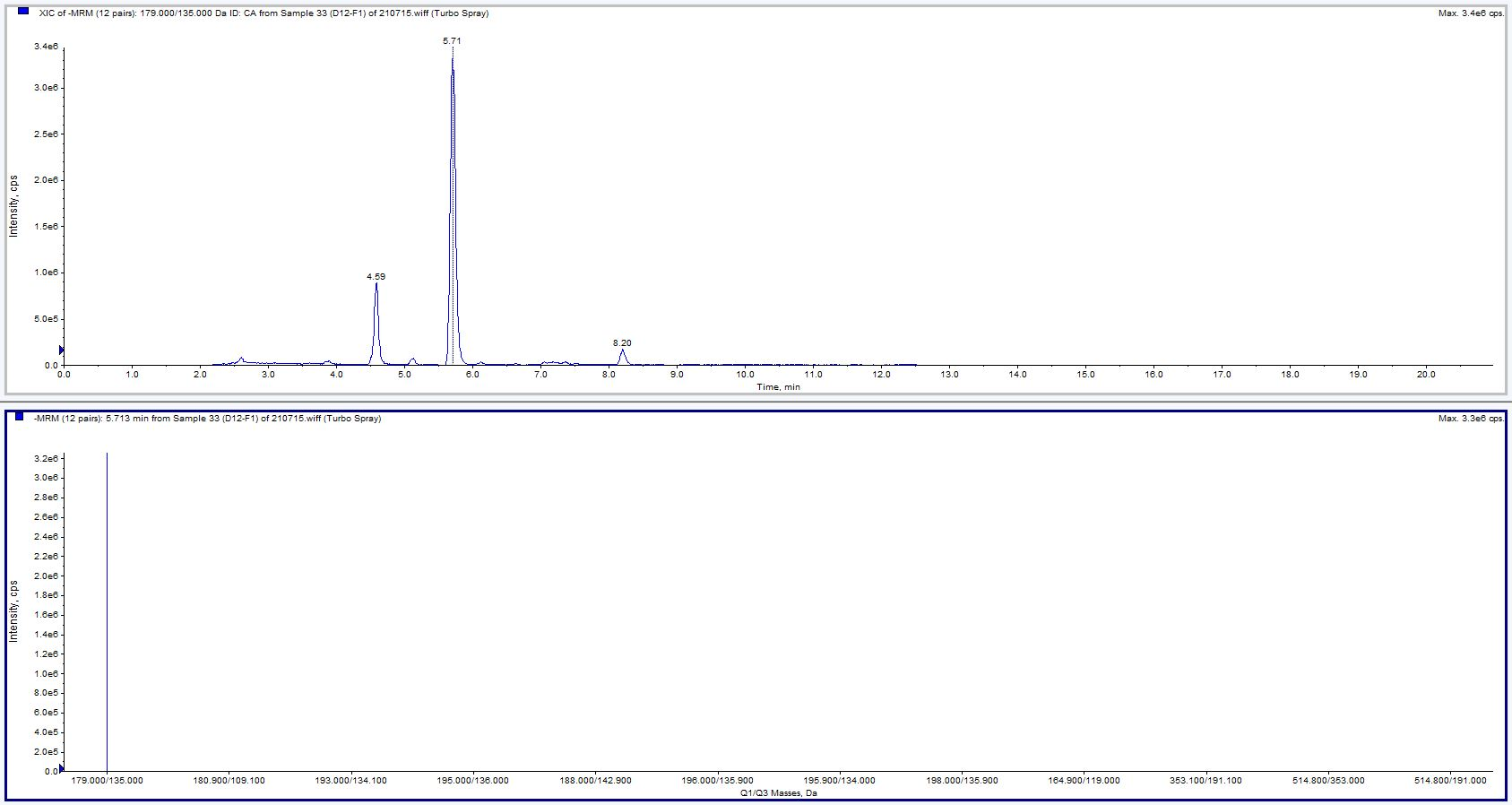

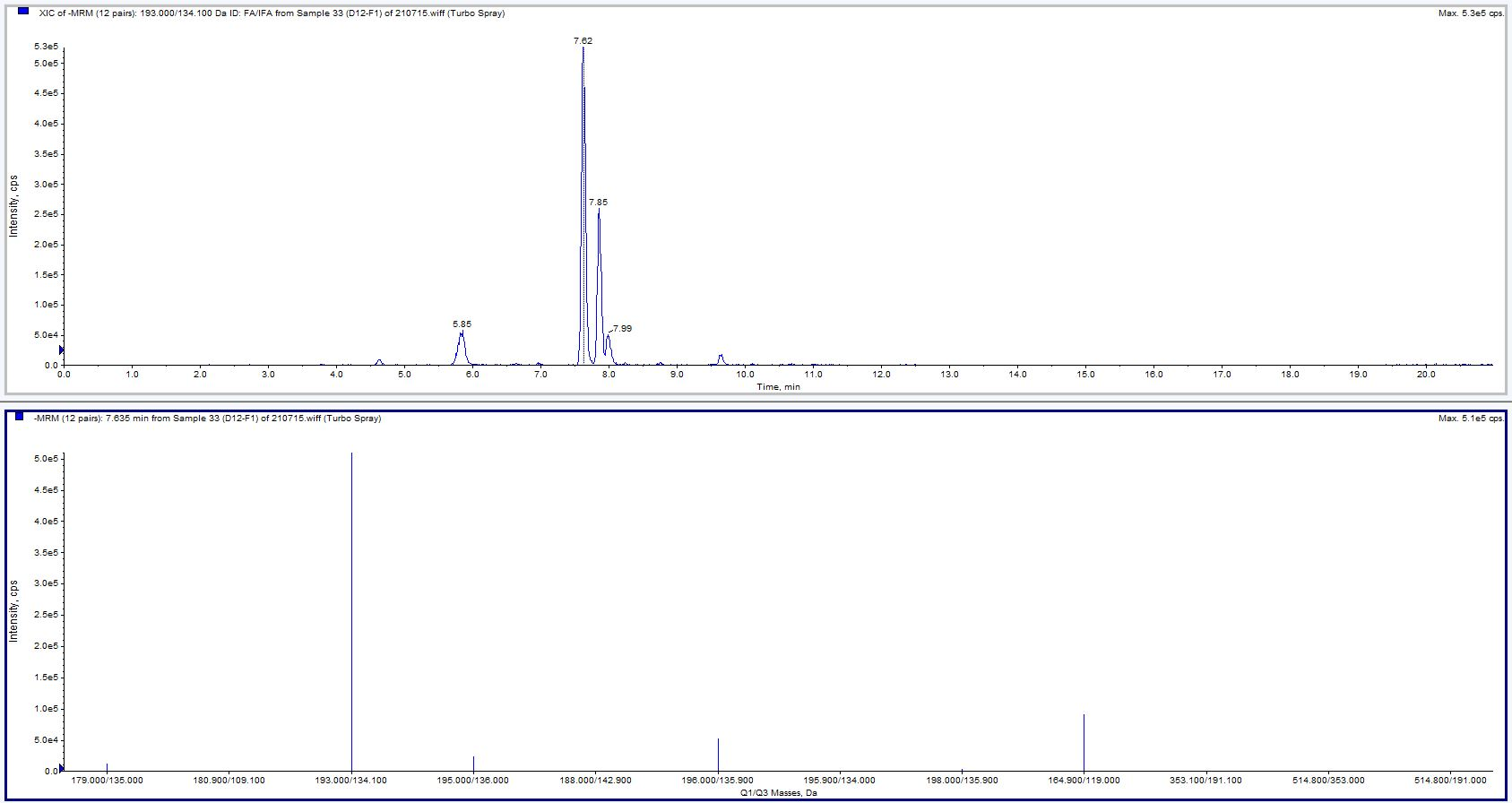


**A**

**A**

**B**

**B**

**C**

**C**


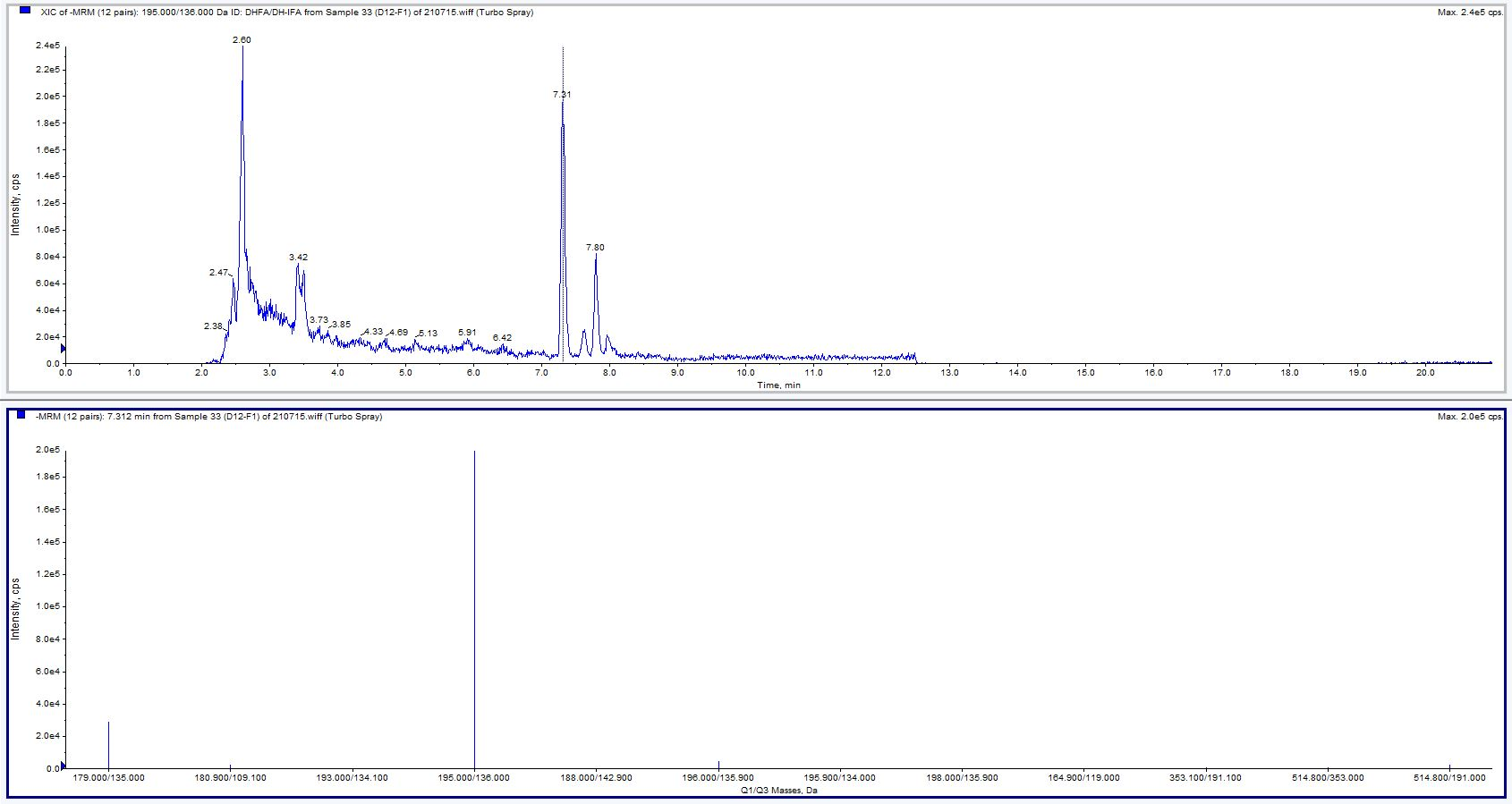

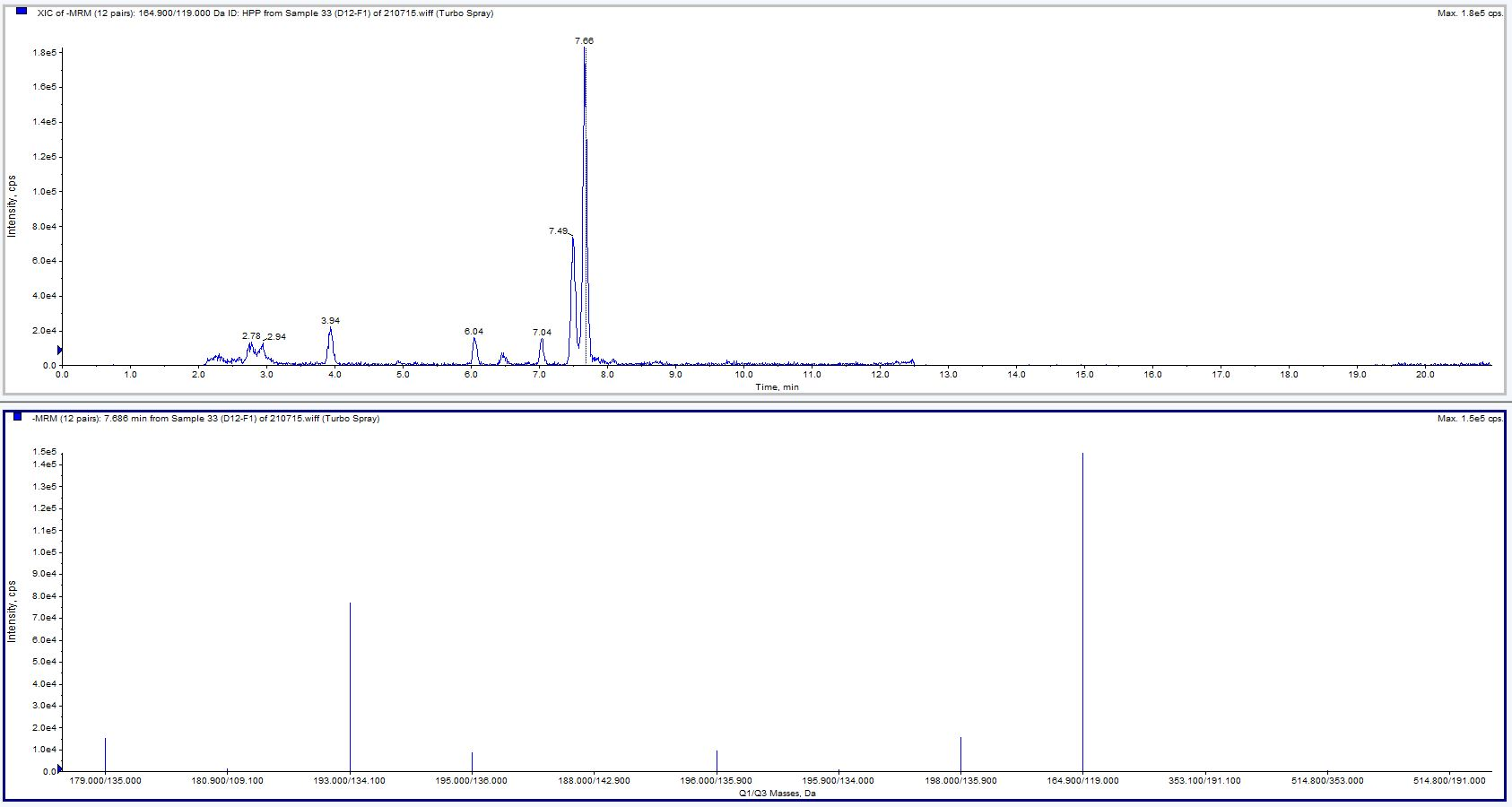


**E**

**E**

**F**

**F**


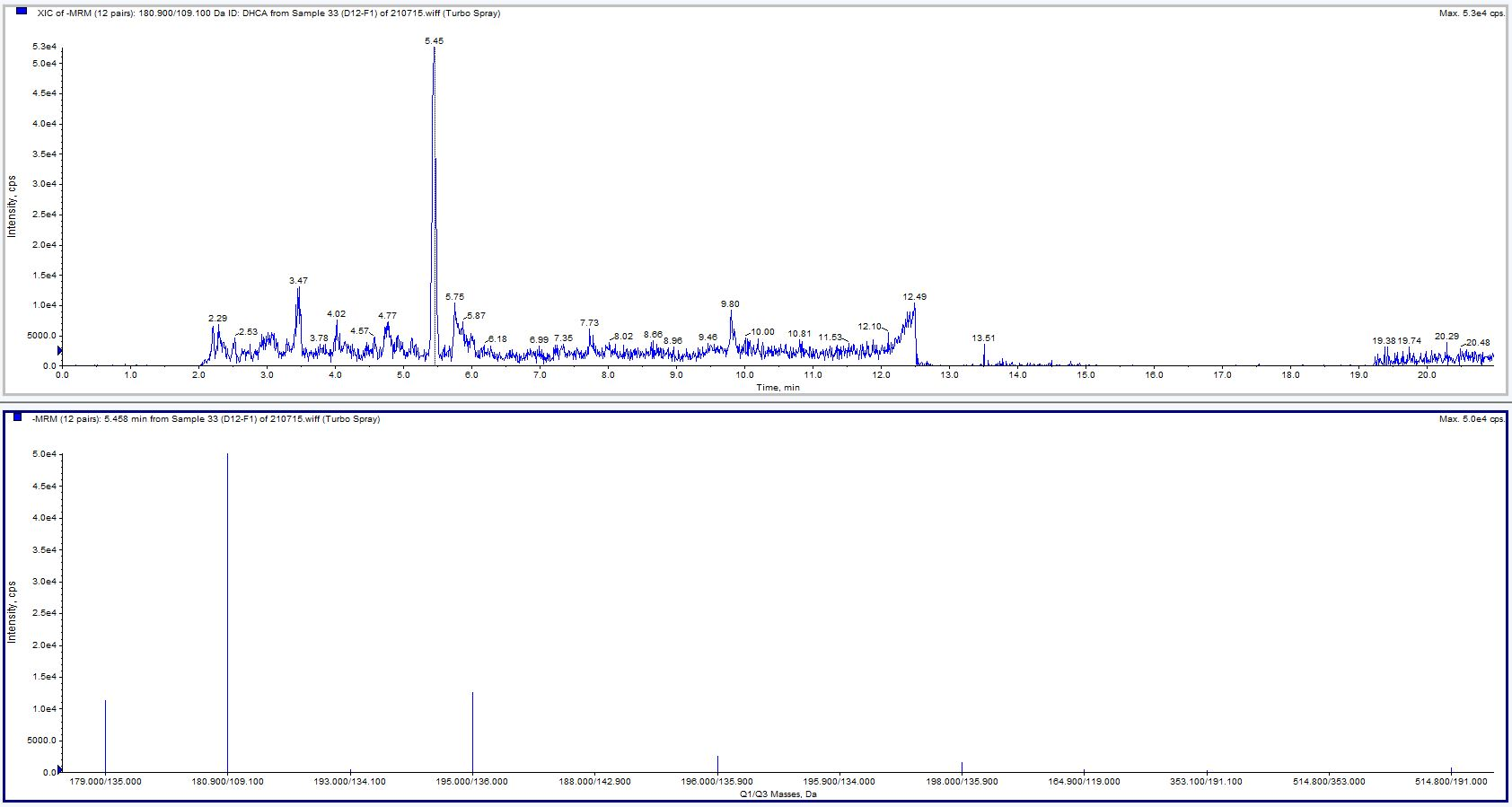


**D**

**D**


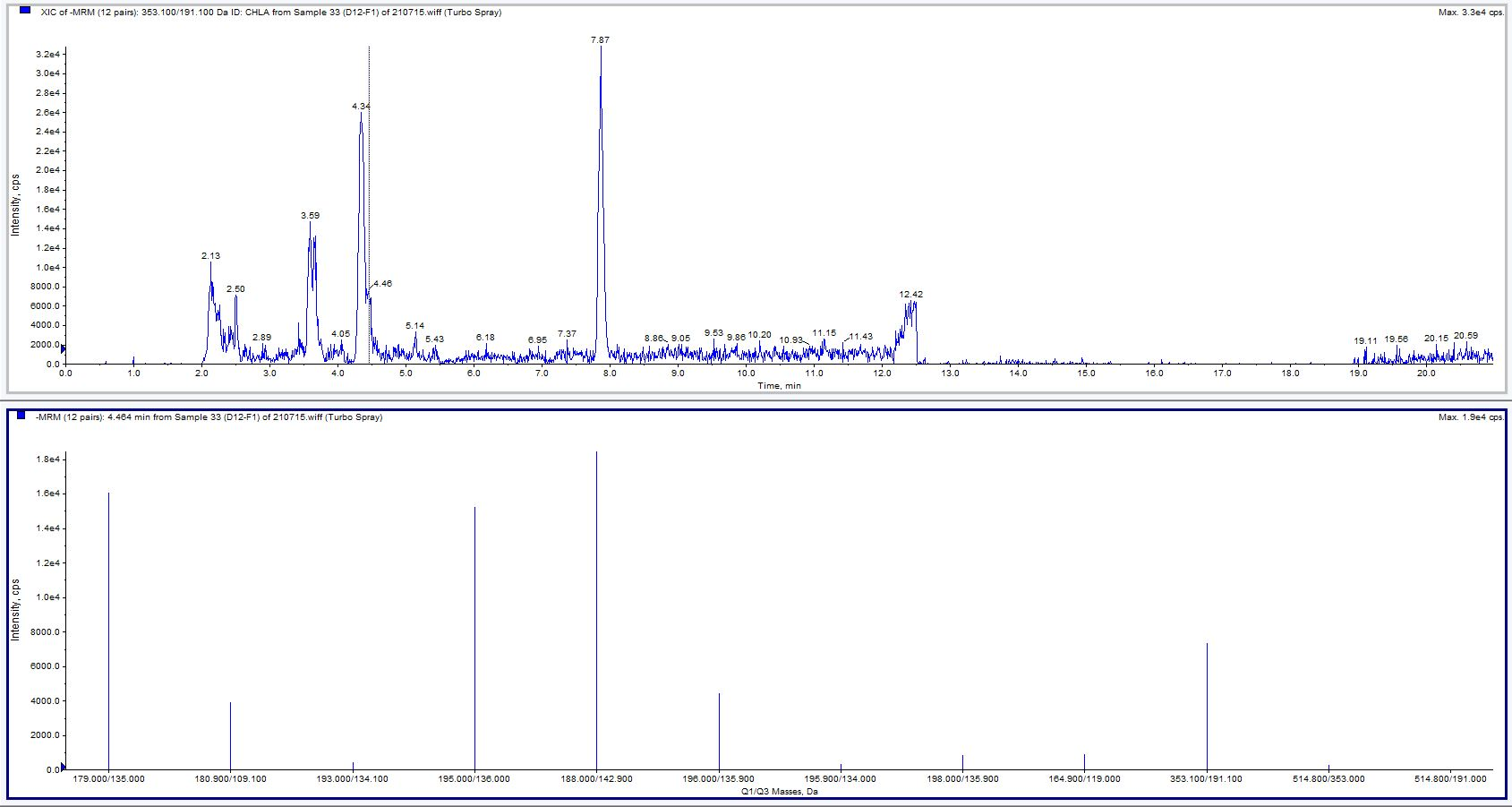


**I**

**I**


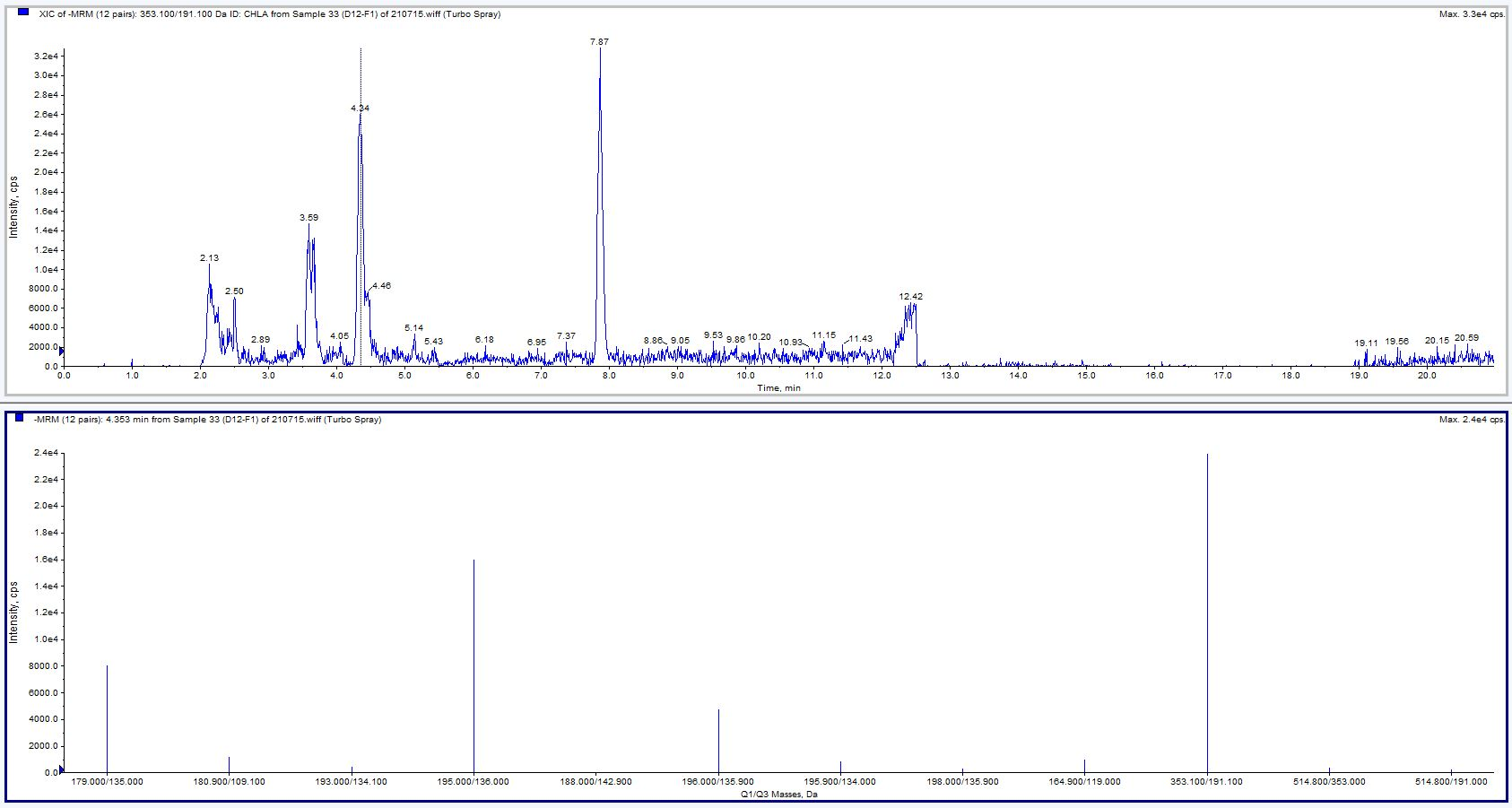

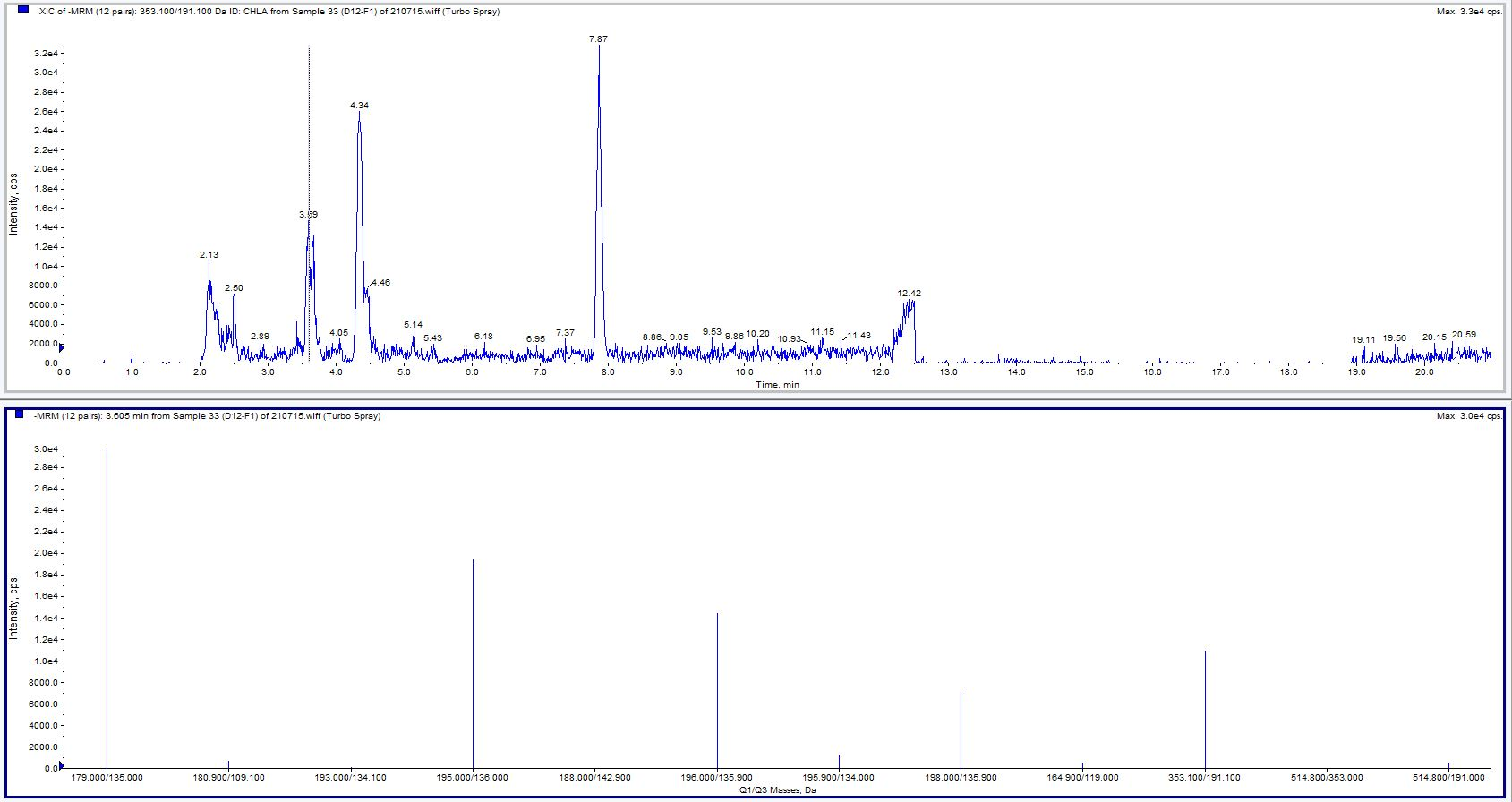


**G**

**H**

**H**

**G**


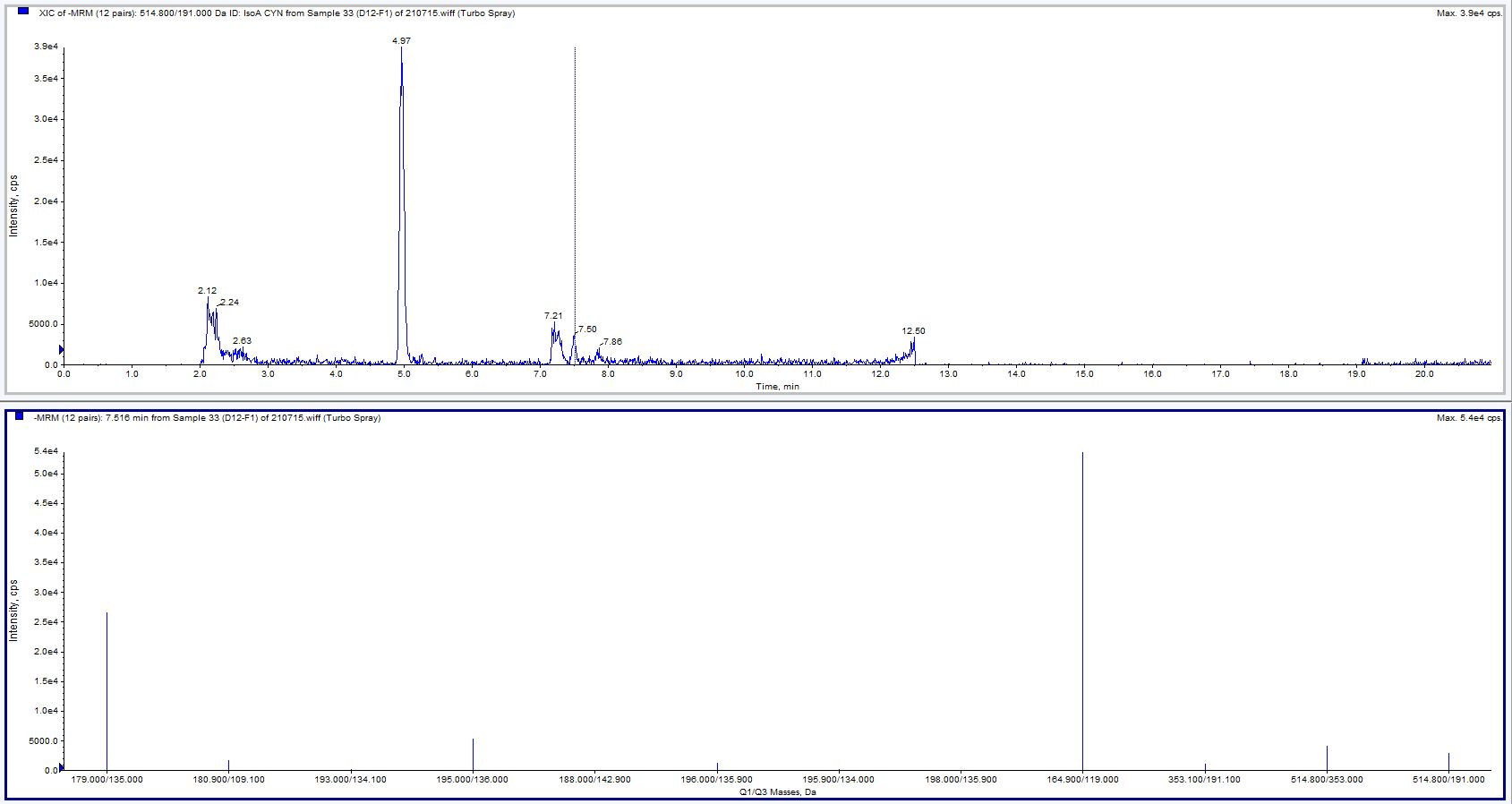

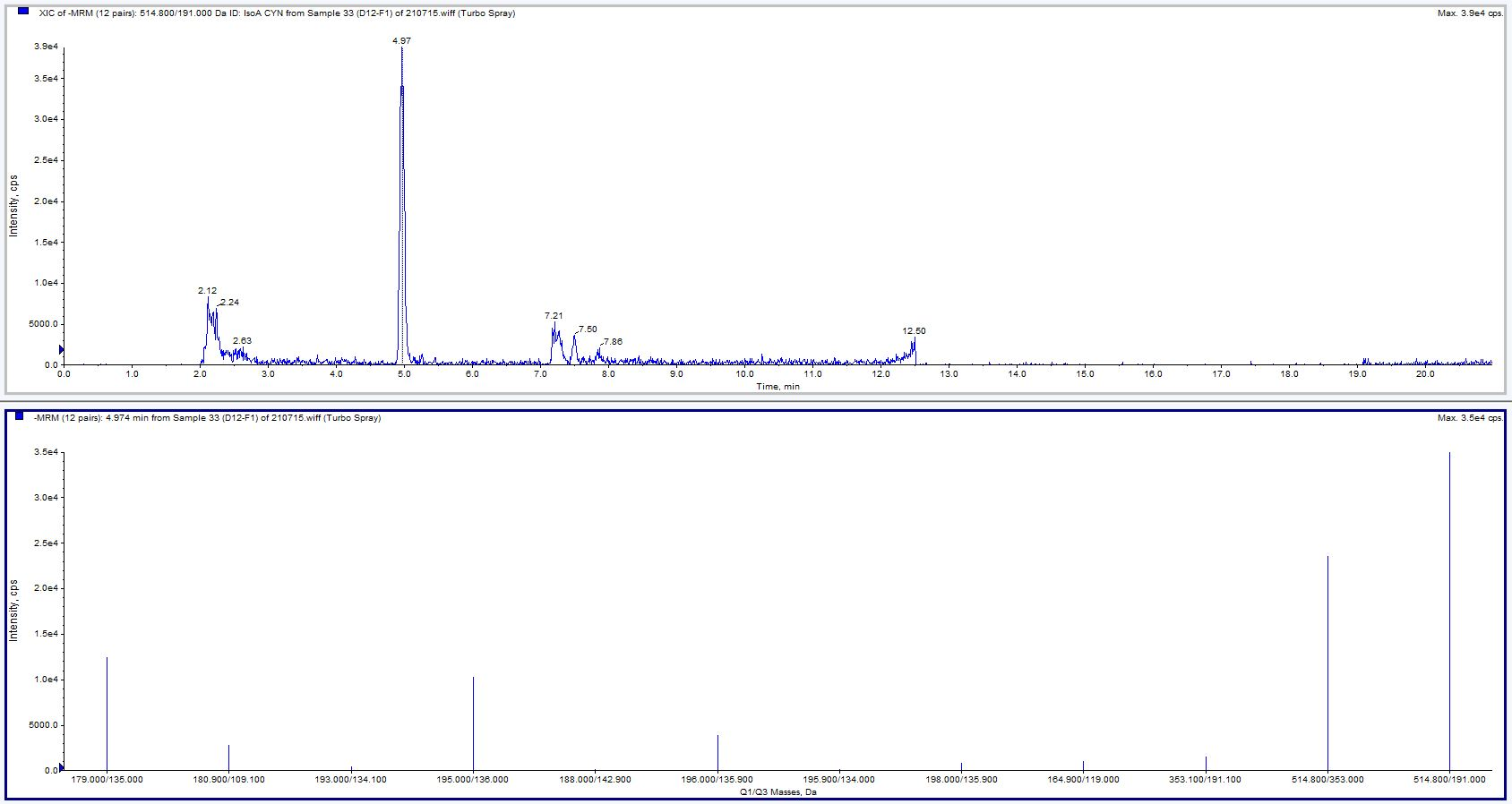

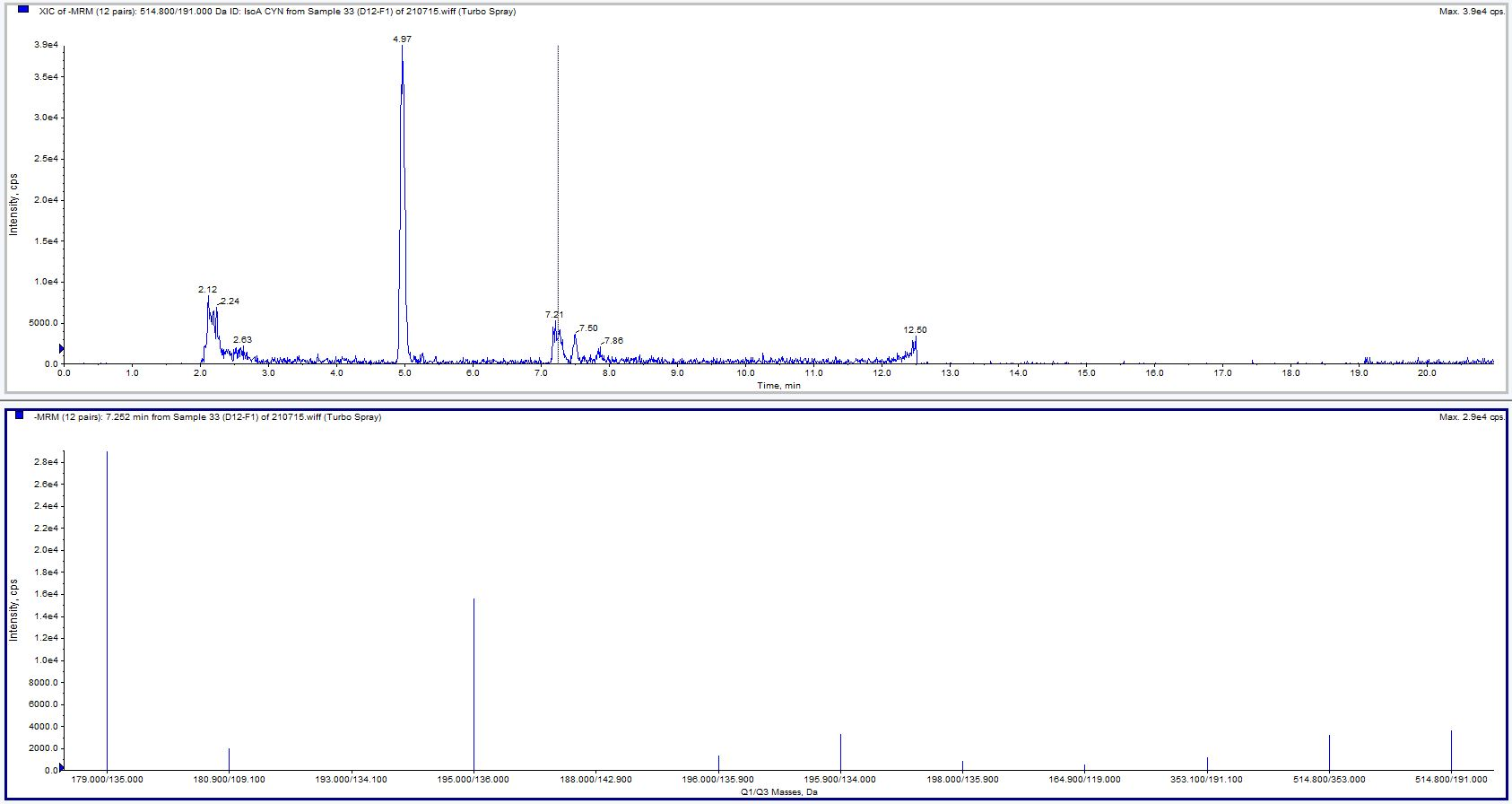


**J**

**J**

**K**

**K**

**L**

**L**

**Supplementary Figure S12.** Chromatograms and spectra of caffeoylquinic acids and metabolites from *Centella asiatica* in spiked human urine. The chromatogram and spectra were obtained in negative ion mode using the following MS/MS transitions (m/z): mono-caffeoylquinic acids (353/191); di-caffeoylquinic acids (515/353; 515/191); caffeic acid (179/135); ferulic acid and isoferulic acid (193/134); dihydrocaffeic acid (181/109); dihydroferulic acid (195/136), 3-(3-hydroxyphenyl)propionic acid (165/106). A) Caffeic acid; B) Ferulic acid; C) isoferulic acid; D) Dihydrocaffeic acid; E) Dihydroferulic acid; F) 3-(3-hydroxyphenyl)propionic acid; G) Neocholorogenic acid; H) Chlorogenic acid; I) Cryptochlorogenic acid; J) 1,3-Di-caffeoylquinic acid/1,4-Di-caffeoylquinic acid/1,5-Dicaffeoylquinic acid; K) Isochlorogenic acid A; L) Isochlorogenic acid B; M) Isochlorogenic acid C


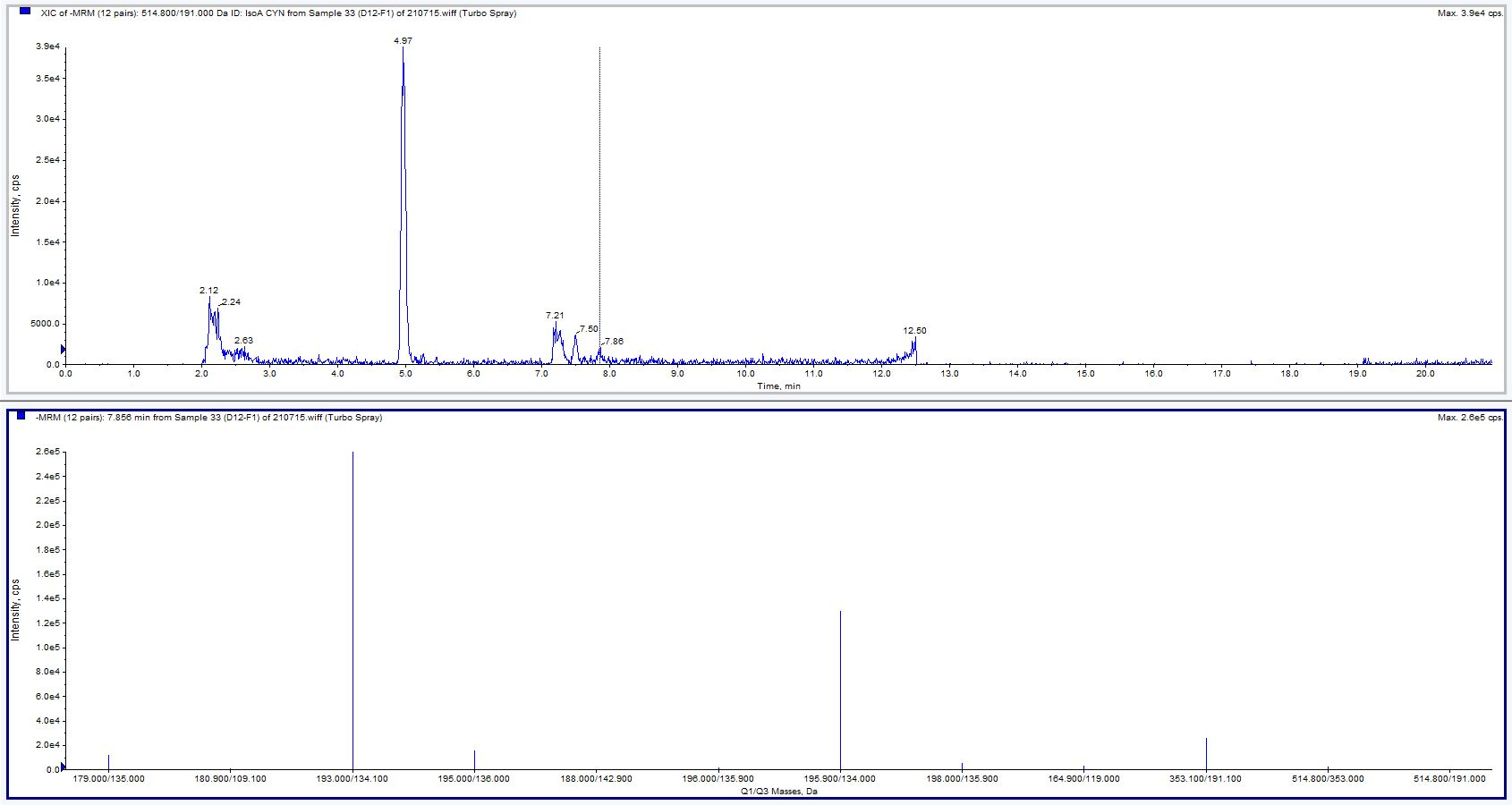


**M**

**M**

# Supplementary Tables

Supplementary Table S1. Intraday precision and accuracy of analytes from *Centella asiatica* in human plasma.

| **Precision (%)** | | | | | | | | | | |
| --- | --- | --- | --- | --- | --- | --- | --- | --- | --- | --- |
|  | **2** | | **3** | | **4** | | **5** | | **6** | |
| **Analyte** | **No IS** | **With IS** | **No IS** | **With IS** | **No IS** | **With IS** | **No IS** | **With IS** | **No IS** | **With IS** |
| **AA** | 4 | 16 | 13 | 11 | 18 | 29 | 27 | 11 | 22 | 8 |
| **AS** | 28 | 48 | 26 | 33 | 13 | 43 | 14 | 25 | 13 | 25 |
| **CA** | 54 | 44 | 6 | 6 | 17 | 6 | 8 | 1 | 10 | 3 |
| **DHCA** | 27 | 20 | 34 | 41 | 50 | 38 | 18 | 23 | 21 | 15 |
| **DHFA** | 24 | 14 | 11 | 6 | 9 | 7 | 9 | 3 | 12 | 2 |
| **Di-CQAs^a^** | 9 | 7 | 2 | 12 | 12 | 7 | 4 | 4 | 6 | 5 |
| **FA** | 16 | 1 | 7 | 5 | 6 | 7 | 2 | 6 | 13 | 3 |
| **HPP** | 15 | 21 | 10 | 6 | 18 | 6 | 7 | 4 | 11 | 2 |
| **IFA** | 18 | 7 | 22 | 12 | 15 | 8 | 14 | 7 | 13 | 4 |
| **MA** | 16 | 1 | 7 | 5 | 6 | 7 | 2 | 6 | 13 | 3 |
| **Mono-CQAs^b^** | 26 | 41 | 22 | 33 | 7 | 6 | 2 | 6 | 5 | 9 |
| **MS** | 27 | 44 | 12 | 36 | 42 | 89 | 42 | 83 | 22 | 54 |
| **Accuracy (% error)** | | | | | | | | | | |
|  | **1** | | **2** | | **3** | | **4** | | **5** | |
| **Analyte** | **No IS** | **With IS** | **No IS** | **With IS** | **No IS** | **With IS** | **No IS** | **With IS** | **No IS** | **With IS** |
| **AA** | 104 | -196 | 107 | 1 | 42 | -8 | 27 | -12 | 13 | -20 |
| **AS** | -399 | -399 | 803 | 471 | -19 | -84 | 6 | -42 | 35 | -13 |
| **CA** | 5 | -24 | 21 | 11 | 10 | 4 | 6 | -5 | 10 | 1 |
| **DHCA** | 149 | 136 | -11 | -14 | -28 | -32 | 3 | -5 | 51 | 39 |
| **DHFA** | 39 | 6 | 18 | 7 | 2 | -3 | 10 | -1 | 13 | 3 |
| **Di-CQAs^a^** | -177 | -225 | 33 | 16 | 2 | -5 | 0 | -12 | 11 | 0 |
| **FA** | 34 | 8 | 11 | 1 | 4 | 0 | 6 | -4 | 10 | 0 |
| **HPP** | 16 | -11 | 30 | 18 | 9 | 2 | 5 | -7 | 10 | 0 |
| **IFA** | 19 | -13 | 10 | -2 | -4 | -8 | 11 | -2 | 8 | -7 |
| **MA** | -53 | -243 | 29 | -44 | 2 | -34 | 30 | -10 | 14 | -20 |
| **Mono-CQAs^b^** | -627 | -716 | 895 | 867 | -40 | -52 | -22 | -37 | 27 | 13 |
| **MS** | -1970 | -6696 | 104 | -1031 | 79 | -241 | 93 | -88 | 290 | 96 |

No IS, results obtained with calibration curves constructed by linear regression analysis of the analyte area vs. the concentration of analytes injected; With IS, results obtained with calibration curves constructed by linear regression analysis of the ratio of the analyte area to the internal standard (IS) area vs. the concentration of analytes injected. Precision is expressed as RSD = (standard deviation/mean concentration measured) x 100. Accuracy was determined as percent error. AA = asiatic acid; AS = asiaticoside; CA = caffeic acid; DHCA = dihydrocaffeic acid; DHFA = dihydroferulic acid; FA = ferulic acid; HPP = 3-(3-hydroxyphenyl)propionic acid; IFA = isoferulic acid; MA = madecassic acid; MS = madecassoside; ^a^ di-caffeoylquinic acids (1,3-dicaffeoylquinic acid (1,3-diCQA), 1,4-dicaffeoylquinic acid (1,4-diCQA), 1,5-dicaffeoylquinic acid (1,5-diCQA), isochlorogenic acid A (IsoA), isochlorogenic acid B (IsoB), isochlorogenic acid C (IsoC)); ^b^ mono-caffeoylquinic acids (chlorogenic acid (CHLA), cryptochlorogenic acid (Crypto), neochlorogenic acid (Neo)).

Supplementary Table S2. Linear equations of calibration curves for asiatic acid and madecassic acid for participants of CAP pharmacokinetic trial.

|  | | | **Area** | | **Area Ratio** | |
| --- | --- | --- | --- | --- | --- | --- |
| **Participant** | **Analyte** | **Visit** | **Linear Equation** | **R^2^** | **Linear Equation** | **R^2^** |
| 1 | AA | 1 | y = 693.59x + 8165.3 | 0.99 | y = 0.011x + 0.6454 | 0.97 |
|  |  | 2 | y = 806.71x - 817.57 | 0.98 | y = 0.0118x - 0.155 | 0.98 |
|  | MA | 1 | y = 405x + 2570.4 | 0.99 | y = 0.0064x + 0.1838 | 0.97 |
|  |  | 2 | y = 456.85x + 82.214 | 0.98 | y = 0.0067x - 0.0374 | 0.99 |
| 2 | AA | 1 | y = 957.73x + 41045 | 0.99 | y = 0.0058x + 0.3544 | 0.98 |
|  |  | 2 | y = 1069.6x + 25541 | 0.97 | y = 0.0066x + 0.0725 | 0.99 |
|  | MA | 1 | y = 537.61x + 3157.6 | 0.99 | y = 0.0033x + 0.0447 | 0.99 |
|  |  | 2 | y = 593.69x + 3678.4 | 0.98 | y = 0.0036x + 0.0014 | 0.99 |
| 3 | AA | 1 | y = 668.67x – 20378 | 0.93 | y = 0.0076x + 0.4832 | 0.95 |
|  |  | 2 | y = 610.24x + 14901 | 0.71 | y = 0.011x + 0.0864 | 0.99 |
|  | MA | 1 | y = 399.04x - 8285.8 | 0.93 | y = 0.0043x + 0.1047 | 0.98 |
|  |  | 2 | y = 338.63x + 3524.7 | 0.66 | y = 0.006x + 0.0042 | 0.99 |
| 4 | AA | 1 | y = 245.36x + 42069 | 0.70 | y = 0.0148x - 0.042 | 0.99 |
|  |  | 2 | y = 246.54x + 41060 | 0.70 | y = 0.0149x - 0.1721 | 0.99 |
|  | MA | 1 | y = 124.6x + 7368.7 | 0.79 | y = 0.0073x - 0.0643 | 0.99 |
|  |  | 2 | y = 124.49x + 7379.2 | 0.78 | y = 0.0074x - 0.0903 | 0.99 |

R^2^ = correlation coefficient. AA = asiatic acid, MA = madecassic acid
